# Supplementary material for: The nucleolar protein NIFK promotes cancer progression via CK1α/β-catenin in metastasis and Ki-67-dependent cell proliferation
Source: eLife. 2016 Mar 17;5:e11288. doi: 10.7554/eLife.11288 (PMC4811767; doi:10.7554/eLife.11288)
Supplement: Supplementary file 1. — (B) Signature alternations after lentiviral-based NIFK overexpression in PC13 cell were listed. DOI: http://dx.doi.org/10.7554/eLife.11288.023 [file elife-11288-supp1.docx]

**Supplementary files**

**Supplementary file 1A**

| **#Fold-Change cut-off : 1.5** | |  |  |
| --- | --- | --- | --- |
| **#Pairing option :**  **All against single sample** | | |  |
| **#Condition pairs :** |  |  |  |
| **[NIFK.CEL] vs [vector.CEL]** | | |  |
| **# Technology : Affymetrix.GeneChip.HG-U133_Plus_2.0** | | | |

| **Probe Set ID** | **Fold change** | **Regulation** | **Gene Symbol** |
| --- | --- | --- | --- |
| 224714_at | 6.738868 | up | MKI67IP |
| 210102_at | 1.826137 | up | VWA5A |
| 219655_at | 1.760785 | up | C7orf10 |
| 233742_at | 1.754861 | up | C16orf68 |
| 231915_at | 1.731185 | up | ZSWIM4 |
| 1569372_at | 1.720069 | up | LOC100507194 |
| 205484_at | 1.71612 | up | SIT1 |
| 224321_at | 1.685396 | up | TMEFF2 |
| 207321_s_at | 1.679766 | up | ABCB9 |
| 243287_s_at | 1.661394 | up | OSTM1 |
| 203872_at | 1.660203 | up | ACTA1 |
| 231544_s_at | 1.655128 | up | POLR3G |
| 207005_s_at | 1.652045 | up | BCL2 |
| 232650_at | 1.643349 | up | C12orf65 |
| 219668_at | 1.639954 | up | GDAP1L1 |
| 238326_at | 1.639954 | up | ODF3B |
| 216866_s_at | 1.633214 | up | COL14A1 |
| 205593_s_at | 1.629496 | up | PDE9A |
| 213559_s_at | 1.629496 | up | ZNF467 |
| 239097_at | 1.625749 | up | C9orf4 |
| 204187_at | 1.62507 | up | GMPR |
| 234031_at | 1.619663 | up | C20orf112 |
| 215719_x_at | 1.619255 | up | FAS |
| 1569323_at | 1.619255 | up | PTPRG |
| 207072_at | 1.610724 | up | IL18RAP |
| 1564372_s_at | 1.610466 | up | CASC2 |
| 1556894_at | 1.604558 | up | NT5DC2 |
| 215470_at | 1.596971 | up | GTF2H2B |
| 232519_at | 1.590626 | up | NSFL1C |
| 1557003_at | 1.587217 | up | TTC23L |
| 207434_s_at | 1.585603 | up | FXYD2 |
| 228524_at | 1.583349 | up | ADCK5 |
| 1553069_at | 1.577002 | up | NCRNA00257 |
| 207865_s_at | 1.57201 | up | BMP8B |
| 223438_s_at | 1.569588 | up | PPARA |
| 206144_at | 1.569588 | up | MAGI1 |
| 233911_s_at | 1.565642 | up | PPM1H |
| 1558189_a_at | 1.561694 | up | LOC284570 |
| 214673_s_at | 1.561369 | up | HUWE1 |
| 206868_at | 1.555221 | up | STARD8 |
| 226940_at | 1.550759 | up | FAM69B |
| 244442_at | 1.548729 | up | HORMAD2 |
| 215668_s_at | 1.548723 | up | PLXNB1 |
| 1552484_at | 1.542357 | up | SMCR8 |
| 234054_at | 1.540031 | up | LOC143188 |
| 209755_at | 1.540031 | up | NMNAT2 |
| 220090_at | 1.540006 | up | CRNN |
| 219759_at | 1.538753 | up | ERAP2 |
| 220119_at | 1.53449 | up | EPB41L4A |
| 226819_at | 1.534115 | up | LSM11 |
| 220471_s_at | 1.532827 | up | MYCT1 |
| 210895_s_at | 1.532827 | up | CD86 |
| 232128_s_at | 1.532827 | up | CLCN5 |
| 224204_x_at | 1.531757 | up | ARNTL2 |
| 209040_s_at | 1.530903 | up | PSMB8 |
| 219407_s_at | 1.528442 | up | LAMC3 |
| 207797_s_at | 1.527656 | up | LRP2BP |
| 219964_at | 1.522959 | up | ST7L |
| 224181_at | 1.522533 | up | C18orf2 |
| 217469_at | 1.522244 | up | IGHA1 |
| 230325_at | 1.522108 | up | LOC100133985 |
| 215819_s_at | 1.521743 | up | RHCE /// RHD |
| 1559705_s_at | 1.520341 | up | PHKA2 |
| 1562563_at | 1.518864 | up | HCN1 |
| 1565537_at | 1.516406 | up | NKX1-1 |
| 234855_at | 1.514865 | up | ORF1 |
| 1558779_at | 1.514127 | up | H2AFY |
| 214825_at | 1.513006 | up | FAM155A |
| 223832_s_at | 1.509825 | up | CAPNS2 |
| 229311_at | 1.504727 | up | GKAP1 |
| 1552315_at | 1.502684 | up | GIMAP1 |
| 215531_s_at | 1.500931 | up | GABRA5 /// LOC100509612 |
| 240475_at | 1.50046 | up | EGOT |
| 207874_s_at | -1.50046 | down | CFHR4 |
| 243664_at | -1.50046 | down | TXNL1 |
| 1554198_at | -1.50389 | down | SH3YL1 |
| 230229_at | -1.50444 | down | DLG1 |
| 216983_s_at | -1.50448 | down | ZNF224 |
| 227510_x_at | -1.50448 | down | MALAT1 |
| 222719_s_at | -1.50473 | down | PDGFC |
| 1557081_at | -1.50664 | down | RBM25 |
| 226675_s_at | -1.50687 | down | MALAT1 |
| 219791_s_at | -1.50702 | down | NBLA00301 |
| 224496_s_at | -1.50982 | down | TMEM107 |
| 1566147_a_at | -1.51228 | down | LOC375010 |
| 236609_at | -1.51301 | down | PMS1 |
| 207343_at | -1.51359 | down | LYZL6 |
| 227074_at | -1.51433 | down | LOC100131564 |
| 1553775_at | -1.51521 | down | FLJ31715 |
| 228381_at | -1.51659 | down | LOC100287628 |
| 239041_at | -1.51802 | down | HIST1H2AK |
| 213672_at | -1.51878 | down | MARS |
| 204270_at | -1.51878 | down | SKI |
| 224565_at | -1.51951 | down | NEAT1 |
| 1562829_at | -1.52018 | down | LOC339568 |
| 209854_s_at | -1.52034 | down | KLK2 |
| 203990_s_at | -1.52217 | down | KDM6A |
| 209242_at | -1.52253 | down | PEG3 |
| 214675_at | -1.52253 | down | NUP188 |
| 224566_at | -1.52346 | down | NEAT1 |
| 216100_s_at | -1.52528 | down | TOR1AIP1 |
| 234539_at | -1.5342 | down | ADARB1 |
| 213856_at | -1.53648 | down | CD47 |
| 239710_at | -1.53648 | down | FIGN |
| 235405_at | -1.53677 | down | GSTA4 |
| 1555659_a_at | -1.53774 | down | TREML1 |
| 224559_at | -1.53849 | down | MALAT1 |
| 235732_at | -1.53875 | down | ZNF704 |
| 205326_at | -1.539 | down | RAMP3 |
| 214048_at | -1.53933 | down | MBD4 |
| 215187_at | -1.54003 | down | FLJ11292 |
| 1556950_s_at | -1.54156 | down | SERPINB6 |
| 1553063_at | -1.54281 | down | GPR78 |
| 210584_s_at | -1.5461 | down | POLDIP3 /// RRP7B |
| 1559139_at | -1.54674 | down | NOC2L |
| 209006_s_at | -1.54709 | down | C1orf63 |
| 1554023_s_at | -1.5476 | down | CCDC13 |
| 221919_at | -1.54916 | down | LOC100506653 |
| 240908_at | -1.55052 | down | LOC100507153 |
| 234297_at | -1.55255 | down | RGS8 /// SDHAP3 |
| 211490_at | -1.55357 | down | ADRA1A |
| 217602_at | -1.55357 | down | PPIA |
| 233122_at | -1.56076 | down | KRTCAP2 |
| 1561501_s_at | -1.56076 | down | CTU2 |
| 236953_s_at | -1.56261 | down | NHLRC3 |
| 209189_at | -1.56287 | down | FOS |
| 233454_at | -1.56294 | down | POLN |
| 217104_at | -1.56421 | down | ST20 |
| 229899_s_at | -1.56421 | down | NCRNA00275 |
| 241433_at | -1.56552 | down | RCOR3 |
| 1569402_at | -1.56729 | down | MEIS3 |
| 223581_at | -1.56824 | down | ZNF577 |
| 242702_at | -1.56953 | down | MMAA |
| 213797_at | -1.56953 | down | RSAD2 |
| 236219_at | -1.57072 | down | TMEM20 |
| 1557227_s_at | -1.57167 | down | TPR |
| 213176_s_at | -1.57552 | down | LTBP4 |
| 1556514_at | -1.57552 | down | C12orf74 |
| 211824_x_at | -1.58122 | down | NLRP1 |
| 223578_x_at | -1.58559 | down | MALAT1 |
| 205522_at | -1.5856 | down | HOXD3 /// HOXD4 /// MIR10B |
| 213517_at | -1.58585 | down | PCBP2 |
| 1560879_a_at | -1.59185 | down | SYT15 |
| 230106_at | -1.59361 | down | ZXDC |
| 231108_at | -1.59384 | down | FUS |
| 1558678_s_at | -1.59424 | down | MALAT1 |
| 1556006_s_at | -1.59514 | down | CSNK1A1 |
| 1552452_at | -1.59844 | down | WDR88 |
| 1559593_a_at | -1.60067 | down | MED26 |
| 223746_at | -1.60213 | down | STK4 |
| 1557569_at | -1.60375 | down | MPDU1 |
| 229110_at | -1.61001 | down | SLC24A2 |
| 1557131_at | -1.61001 | down | LOC254100 |
| 242361_at | -1.61047 | down | IMMT |
| 1556227_at | -1.61688 | down | VCPIP1 |
| 232173_at | -1.61794 | down | CLEC2L |
| 1564467_at | -1.61926 | down | FAM161A |
| 213703_at | -1.61949 | down | LOC150759 |
| 217538_at | -1.6198 | down | SGSM2 |
| 242918_at | -1.62029 | down | NASP |
| 205931_s_at | -1.62134 | down | CREB5 |
| 227383_at | -1.62325 | down | LOC727820 |
| 243683_at | -1.62682 | down | MORF4L2 |
| 214694_at | -1.63548 | down | MPRIP |
| 219423_x_at | -1.63577 | down | TNFRSF25 |
| 224117_at | -1.65108 | down | LOC284912 |
| 237086_at | -1.65506 | down | FOXA1 |
| 243128_at | -1.65513 | down | ZNF175 |
| 241403_at | -1.65593 | down | CLK4 |
| 229193_at | -1.65604 | down | LUC7L3 |
| 1554670_at | -1.65918 | down | GGA1 |
| 1559096_x_at | -1.65976 | down | FBXO9 |
| 1559449_a_at | -1.65992 | down | ZNF254 |
| 242674_at | -1.66076 | down | EIF4E |
| 210057_at | -1.66471 | down | SMG1 |
| 224568_x_at | -1.6696 | down | MALAT1 |
| 223940_x_at | -1.68404 | down | MALAT1 |
| 1559964_at | -1.68634 | down | FLJ38717 |
| 212386_at | -1.69783 | down | TCF4 |
| 243282_at | -1.70299 | down | CCDC93 |
| 223588_at | -1.71459 | down | THAP2 |
| 214291_at | -1.72833 | down | RPL17 |
| 1561973_at | -1.74586 | down | SMARCC2 |
| 222137_at | -1.74678 | down | CC2D1A |
| 1559094_at | -1.78421 | down | FBXO9 |
| 227384_s_at | -1.79185 | down | LOC727820 |
| 211888_x_at | -1.79597 | down | CASP10 |
| 214705_at | -1.81771 | down | INADL |
| 1557267_s_at | -1.8541 | down | LOC284952 |
| 208157_at | -1.85537 | down | SIM2 |
| 238736_at | -1.87385 | down | REV3L |
| 242828_at | -1.88136 | down | FIGN |
| 243296_at | -1.89274 | down | NAMPT |
| 242146_at | -1.96891 | down | SNRPA1 |
| 243857_at | -2.02723 | down | MORF4L2 |

| **Supplementary file 1B**  **#Fold-Change cut-off : 1.3**  **#Pairing option : All against single sample** | | |
| --- | --- | --- |
| **#[PC13-NIFK-MH Affyx 113.CEL] vs [PC13-RFP-MH Affyx 112.CEL]** | | |
| **# Technology : Affymetrix.GeneChip.HG-U133_Plus_2** | | |
|  |  |  |
| **Probe Set ID** | **Fold change** | **Gene Symbol** |
| 202887_s_at | 7.5502925 | DDIT4 |
| 206115_at | 5.9888687 | EGR3 |
| 205249_at | 3.8042028 | EGR2 |
| 204621_s_at | 3.703549 | NR4A2 |
| 210997_at | 3.6993954 | HGF |
| 227337_at | 3.4686024 | ANKRD37 |
| 217028_at | 3.4168336 | CXCR4 |
| 210998_s_at | 3.2052166 | HGF |
| 216248_s_at | 3.1855018 | NR4A2 |
| 204622_x_at | 3.1806362 | NR4A2 |
| 219270_at | 3.164489 | CHAC1 |
| 211708_s_at | 3.162079 | SCD |
| 202708_s_at | 3.116691 | HIST2H2BE |
| 241426_at | 3.104827 | CEP44 |
| 218976_at | 2.9165742 | DNAJC12 |
| 207768_at | 2.8910687 | EGR4 |
| 223196_s_at | 2.8141973 | SESN2 |
| 223195_s_at | 2.5822787 | SESN2 |
| 202672_s_at | 2.5648108 | ATF3 |
| 203439_s_at | 2.5622418 | STC2 |
| 201295_s_at | 2.5479023 | WSB1 |
| 201694_s_at | 2.5227866 | EGR1 |
| 219132_at | 2.4917078 | PELI2 |
| 227404_s_at | 2.4592855 | EGR1 |
| 226452_at | 2.449797 | PDK1 |
| 203543_s_at | 2.447195 | KLF9 |
| 230210_at | 2.444832 | SUN1 |
| 244070_at | 2.4062748 | SYNE1 |
| 210665_at | 2.3782632 | TFPI |
| 221523_s_at | 2.3428411 | RRAGD |
| 239752_at | 2.3104377 |  |
| 230710_at | 2.2916722 | MIR210HG |
| 225798_at | 2.2619822 | JAZF1 |
| 210755_at | 2.2607741 | HGF |
| 211162_x_at | 2.211541 | SCD |
| 228846_at | 2.1904902 | MXD1 |
| 207361_at | 2.1688437 | HBP1 |
| 229554_at | 2.1244376 | LUM |
| 241529_at | 2.1106484 |  |
| 229953_x_at | 2.1045072 | LCA5 |
| 210664_s_at | 2.0977488 | TFPI |
| 205612_at | 2.0949218 | MMRN1 |
| 202770_s_at | 2.0881603 | CCNG2 |
| 201169_s_at | 2.0870447 | BHLHE40 |
| 203788_s_at | 2.0846171 | SEMA3C |
| 206331_at | 2.08398 | CALCRL |
| 201858_s_at | 2.0788915 | SRGN |
| 238695_s_at | 2.0642383 | RAB39B |
| 204348_s_at | 2.0568862 | AK4///LOC100507855 |
| 203542_s_at | 2.052078 | KLF9 |
| 214329_x_at | 2.0480003 | TNFSF10 |
| 1556361_s_at | 2.0473688 | ANKRD13C |
| 209544_at | 2.0440753 | RIPK2 |
| 236193_at | 2.0227478 | HIST1H2BC |
| 206085_s_at | 2.020759 | CTH |
| 1555561_a_at | 2.0206535 | UGGT2 |
| 213258_at | 2.0132937 | TFPI |
| 230075_at | 1.9979584 | RAB39B |
| 223756_at | 1.9959599 | KANSL3 |
| 211990_at | 1.995322 | HLA-DPA1 |
| 235285_at | 1.9872301 |  |
| 202688_at | 1.9774715 | TNFSF10 |
| 201859_at | 1.9711561 | SRGN |
| 221577_x_at | 1.9647512 | GDF15 |
| 244401_at | 1.9606092 | LCA5 |
| 204347_at | 1.9600158 | AK4///LOC100507855 |
| 214683_s_at | 1.9532874 | CLK1 |
| 201461_s_at | 1.9525803 | MAPKAPK2 |
| 207219_at | 1.950632 | ZFP69B |
| 209676_at | 1.9504875 | TFPI |
| 242006_at | 1.9359319 | LCA5 |
| 203434_s_at | 1.9351648 | MME |
| 206953_s_at | 1.9298953 | LPHN2 |
| 244439_at | 1.9278858 | SPRED1 |
| 206343_s_at | 1.9251446 | NRG1 |
| 232352_at | 1.9236124 | ISL2 |
| 1554594_at | 1.9233288 | ARHGAP27 |
| 238946_at | 1.9050006 |  |
| 211896_s_at | 1.9009186 | DCN |
| 210286_s_at | 1.8979046 | SLC4A7 |
| 205822_s_at | 1.8954378 | HMGCS1 |
| 229090_at | 1.8848562 | ZEB1-AS1 |
| 209566_at | 1.884531 | INSIG2 |
| 202855_s_at | 1.8843442 | SLC16A3 |
| 209189_at | 1.8834937 | FOS |
| 225800_at | 1.8824002 | JAZF1 |
| 217562_at | 1.8763766 | FAM5C |
| 216017_s_at | 1.8757814 | NAB2 |
| 205694_at | 1.8754745 | TYRP1 |
| 224454_at | 1.8748492 | ETNK1 |
| 213988_s_at | 1.8742055 | SAT1 |
| 239669_at | 1.871543 |  |
| 200878_at | 1.8707366 | EPAS1 |
| 225342_at | 1.8665171 | AK4///LOC100507855 |
| 209822_s_at | 1.8647189 | VLDLR |
| 202769_at | 1.8628069 | CCNG2 |
| 209394_at | 1.8523786 | ASMTL |
| 221524_s_at | 1.8484724 | RRAGD |
| 201294_s_at | 1.8450786 | WSB1 |
| 238551_at | 1.8440007 | FUT11 |
| 238575_at | 1.8427864 | OSBPL6 |
| 217478_s_at | 1.8409564 | HLA-DMA |
| 1569729_a_at | 1.8408889 | ASZ1 |
| 202687_s_at | 1.839608 | TNFSF10 |
| 1557309_at | 1.8389733 | DENND1B |
| 1563398_at | 1.8347884 |  |
| 234970_at | 1.8342488 | TC2N |
| 226275_at | 1.8316413 | MXD1 |
| 226682_at | 1.830371 | RORA |
| 217678_at | 1.8296973 | SLC7A11 |
| 205873_at | 1.8270907 | PIGL |
| 225130_at | 1.8270358 | ZRANB1 |
| 242963_at | 1.8253603 | SGMS2 |
| 238542_at | 1.8252486 | ULBP2 |
| 242776_at | 1.8192406 | ZCCHC6 |
| 232636_at | 1.8183488 | SLITRK4 |
| 204749_at | 1.8179978 | NAP1L3 |
| 217924_at | 1.8175304 | C6orf106 |
| 223551_at | 1.8155767 | PKIB |
| 220394_at | 1.8152635 | FGF20 |
| 206103_at | 1.8129526 | RAC3 |
| 237559_at | 1.8125991 | GPR55 |
| 205123_s_at | 1.8103126 | MSANTD3-TMEFF1///TMEFF1 |
| 210592_s_at | 1.8071471 | SAT1 |
| 209921_at | 1.8070277 | SLC7A11 |
| 220717_at | 1.8048687 | ADAMTS20 |
| 227443_at | 1.8025752 | LURAP1L |
| 234996_at | 1.8002973 | CALCRL |
| 243746_at | 1.7973685 | IGHMBP2 |
| 1555279_at | 1.7950644 | ARMC8 |
| 204415_at | 1.7933547 | IFI6 |
| 217168_s_at | 1.7916603 | HERPUD1 |
| 204285_s_at | 1.7912151 | PMAIP1 |
| 203373_at | 1.7908907 | SOCS2 |
| 210941_at | 1.7906462 | PCDH7 |
| 233819_s_at | 1.7898006 | LTN1 |
| 227166_at | 1.7894759 | DNAJC18 |
| 208180_s_at | 1.7878011 | HIST1H4A///HIST1H4B///HIST1H4C///HIST1H4D///HIST1H4E///  HIST1H4F///HIST1H4H///HIST1H4I///HIST1H4J///HIST1H4K///  HIST1H4L///HIST2H4A///HIST2H4B///HIST4H4 |
| 242470_at | 1.7877504 | EID2B |
| 211559_s_at | 1.7854934 | CCNG2 |
| 223930_at | 1.7820562 |  |
| 220195_at | 1.7818854 | MBD5 |
| 1554114_s_at | 1.7790421 | SSH2 |
| 215531_s_at | 1.7720677 | GABRA5 |
| 219736_at | 1.7720674 | TRIM36 |
| 206110_at | 1.7705193 | HIST1H3H |
| 219622_at | 1.7700646 | RAB20 |
| 1555281_x_at | 1.7695802 | ARMC8 |
| 207978_s_at | 1.7691826 | NR4A3 |
| 208546_x_at | 1.7689312 | HIST1H2BH |
| 215723_s_at | 1.7660044 | PLD1 |
| 223713_at | 1.765869 | RSPH3 |
| 230280_at | 1.7633145 | TRIM9 |
| 221291_at | 1.7629234 | ULBP2 |
| 209990_s_at | 1.762568 | GABBR2 |
| 222912_at | 1.7610534 | ARRB1 |
| 206310_at | 1.7605337 | SPINK2 |
| 207046_at | 1.7605098 | HIST1H4A///HIST1H4B///HIST1H4C///HIST1H4D///HIST1H4E///  HIST1H4F///HIST1H4H///HIST1H4I///HIST1H4J///HIST1H4K///  HIST1H4L///HIST2H4A///HIST2H4B///HIST4H4 |
| 201693_s_at | 1.7597722 | EGR1 |
| 230356_at | 1.7595128 | OTTHUMG00000184008///RP13-238F13.5 |
| 244360_at | 1.757844 | FBXL17 |
| 220241_at | 1.7569884 | TMCO3 |
| 36554_at | 1.756986 | ASMTL |
| 235939_at | 1.7552686 | OTTHUMG00000175830///RP1-228H13.5 |
| 212492_s_at | 1.7548735 | KDM4B |
| 214502_at | 1.7539382 | HIST1H2BJ |
| 1552562_at | 1.7521298 | ZNF570 |
| 206907_at | 1.7521298 | TNFSF9 |
| 1570253_a_at | 1.7519011 | RHEBL1 |
| 242617_at | 1.750652 | TMED8 |
| 205279_s_at | 1.7496889 | GLRB |
| 1554640_at | 1.7495049 | PALM2 |
| 36711_at | 1.7494876 | MAFF |
| 243631_at | 1.7473217 |  |
| 203787_at | 1.7451458 | SSBP2 |
| 235457_at | 1.7449647 | MAML2 |
| 204226_at | 1.7443817 | STAU2 |
| 218832_x_at | 1.7442936 | ARRB1 |
| 206931_at | 1.742657 | ZNF141 |
| 243010_at | 1.7415174 | MSI2 |
| 201548_s_at | 1.7406054 | KDM5B |
| 1555324_at | 1.7398933 | PTK7 |
| 1559954_s_at | 1.7395 | DDX42 |
| 237789_at | 1.738246 | OTTHUMG00000009988///RP11-67L3.4 |
| 228603_at | 1.7369739 | ACTR3 |
| 203140_at | 1.7367529 | BCL6 |
| 204753_s_at | 1.7362845 | HLF |
| 209383_at | 1.7360848 | DDIT3 |
| 205493_s_at | 1.7360733 | DPYSL4 |
| 236261_at | 1.7359345 | OSBPL6 |
| 214975_s_at | 1.7322416 | MTMR1 |
| 223805_at | 1.7293779 | OSBPL6 |
| 236042_at | 1.7287859 | LOC100130219 |
| 219312_s_at | 1.7252593 | ZBTB10 |
| 205637_s_at | 1.7218493 | SH3GL3 |
| 202552_s_at | 1.7205153 | CRIM1 |
| 214540_at | 1.7200605 | HIST1H2BO |
| 231898_x_at | 1.7196455 | SOX2-OT |
| 218309_at | 1.7182138 | CAMK2N1 |
| 218319_at | 1.7175478 | PELI1 |
| 210426_x_at | 1.7166346 | RORA |
| 209840_s_at | 1.7165736 | LRRN3 |
| 203455_s_at | 1.7163963 | SAT1 |
| 1555606_a_at | 1.7157356 | GDPD1 |
| 224218_s_at | 1.7107983 | TRPS1 |
| 1556309_s_at | 1.7088317 | C1orf86 |
| 1562988_at | 1.7088317 | ZSCAN30 |
| 235556_at | 1.7043381 | CREBRF |
| 236543_at | 1.7024326 |  |
| 225956_at | 1.7013599 | CREBRF |
| 230201_at | 1.7005863 | FXR1 |
| 241703_at | 1.7001749 | RUNDC3B |
| 215286_s_at | 1.7001468 | PHTF2 |
| 1554103_at | 1.697229 |  |
| 228698_at | 1.6968302 | SOX7 |
| 229248_at | 1.6962509 | UCHL5 |
| 226234_at | 1.696219 | GDF11 |
| 244321_at | 1.6954645 | PGAP1 |
| 243179_at | 1.6944897 |  |
| 212102_s_at | 1.693469 | KPNA6 |
| 223853_at | 1.6925061 | BVES |
| 214455_at | 1.6924278 | HIST1H2BC///HIST1H2BE///HIST1H2BF///HIST1H2BG///  HIST1H2BI |
| 222763_s_at | 1.6909069 | SFT2D3///WDR33 |
| 218280_x_at | 1.6901537 | HIST2H2AA3///HIST2H2AA4 |
| 235889_at | 1.6879653 |  |
| 232023_at | 1.6872692 | TMEM67 |
| 232263_at | 1.6872268 | SLC6A15 |
| 241708_at | 1.6867228 | DOCK1 |
| 220123_at | 1.6860824 | SLC35F5 |
| 235980_at | 1.6860824 | PIK3CA |
| 205376_at | 1.6841218 | INPP4B |
| 220014_at | 1.6836126 | PRR16 |
| 219094_at | 1.6828115 | ARMC8 |
| 221031_s_at | 1.6825312 | APOLD1 |
| 214977_at | 1.6820241 |  |
| 205282_at | 1.6818857 | LRP8 |
| 228030_at | 1.679632 | RBM6 |
| 230874_at | 1.6793754 | SLC36A4 |
| 221478_at | 1.6788071 | BNIP3L |
| 233066_at | 1.6781628 | AP000560.3///OTTHUMG00000165536 |
| 206708_at | 1.6774715 | FOXN2 |
| 212486_s_at | 1.6773086 | FYN |
| 233899_x_at | 1.6767094 | ZBTB10 |
| 222088_s_at | 1.6737897 | SLC2A14///SLC2A3 |
| 1555106_a_at | 1.6729546 | CTDSPL2 |
| 206307_s_at | 1.6725515 | FOXD1 |
| 208763_s_at | 1.6716557 | TSC22D3 |
| 228766_at | 1.6710944 | CD36 |
| 211083_s_at | 1.6702341 | MAP3K13 |
| 230650_at | 1.6702341 | OTTHUMG00000173252///RP11-102F4.3 |
| 1557166_at | 1.6702338 | PDCD4 |
| 213537_at | 1.6702338 | HLA-DPA1 |
| 217127_at | 1.6694868 | CTH |
| 219828_at | 1.6690211 | RABL6 |
| 215629_s_at | 1.6689329 | DLEU2///DLEU2L |
| 225685_at | 1.6673472 | CDC42EP3 |
| 223982_s_at | 1.666832 | PNPLA8 |
| 219763_at | 1.6658114 | DENND1A |
| 219738_s_at | 1.6624398 | PCDH9 |
| 234131_at | 1.6623101 |  |
| 205193_at | 1.6616175 | MAFF |
| 209545_s_at | 1.6587306 | RIPK2 |
| 207767_s_at | 1.6579096 | EGR4 |
| 244514_at | 1.657909 |  |
| 228716_at | 1.6573229 | THRB |
| 200831_s_at | 1.656526 | SCD |
| 203650_at | 1.6561078 | PROCR |
| 231894_at | 1.655462 | SARS |
| 242228_at | 1.653923 |  |
| 1555912_at | 1.6473728 | ST7-AS1 |
| 1555168_a_at | 1.647191 | CALN1 |
| 209447_at | 1.6468943 | SYNE1 |
| 239596_at | 1.6460193 | SLC30A7 |
| 1557349_at | 1.6443144 | RERE |
| 65133_i_at | 1.6438723 | INO80B///INO80B-WBP1 |
| 210572_at | 1.6424024 | PCDHA2 |
| 36553_at | 1.6416115 | ASMTL |
| 230508_at | 1.6414905 | DKK3 |
| 236033_at | 1.6398723 | ASB12 |
| 204899_s_at | 1.6394773 | SAP30 |
| 218854_at | 1.6378876 | DSE |
| 231861_at | 1.6370308 | LRP10 |
| 227038_at | 1.6336807 | SGMS2 |
| 229657_at | 1.633549 | THRB |
| 216607_s_at | 1.6327177 | CYP51A1///LRRD1 |
| 237914_s_at | 1.6325763 |  |
| 229428_at | 1.6310393 | TIMM23 |
| 1564790_at | 1.6294955 | ST7-AS2 |
| 211087_x_at | 1.6285871 | MAPK14 |
| 244879_at | 1.6278789 |  |
| 221367_at | 1.6275061 | MOS |
| 235587_at | 1.6268995 | LOC202781 |
| 232777_s_at | 1.6264088 | C6orf118 |
| 201893_x_at | 1.6264075 | DCN |
| 219569_s_at | 1.6256927 | SLC35G2 |
| 235174_s_at | 1.6250073 | LOC100128822 |
| 240206_at | 1.6248204 | TARS |
| 238622_at | 1.6232862 | RAP2B |
| 218943_s_at | 1.6227927 | DDX58 |
| 216236_s_at | 1.6215402 | SLC2A14///SLC2A3 |
| 235046_at | 1.6213965 | INPP4B |
| 227837_at | 1.6200826 |  |
| 210874_s_at | 1.6199331 | NAT6 |
| 209838_at | 1.6175454 | COPS2 |
| 228915_at | 1.6158082 | DACH1 |
| 1557754_at | 1.6158029 | LOC401068 |
| 202082_s_at | 1.6156597 | SEC14L1 |
| 210256_s_at | 1.6153148 | PIP5K1A |
| 1554472_a_at | 1.6146332 | PHF20L1 |
| 205503_at | 1.6143082 | PTPN14 |
| 230560_at | 1.6141247 | STXBP6 |
| 210298_x_at | 1.6138804 | FHL1 |
| 215253_s_at | 1.6138388 | RCAN1 |
| 223302_s_at | 1.6133796 | ZNF655 |
| 201744_s_at | 1.6121072 | LUM |
| 238396_at | 1.6111654 | RPS23P6///RPS23P6 |
| 230764_at | 1.6102258 |  |
| 202498_s_at | 1.6095431 | SLC2A3 |
| 228561_at | 1.6092538 | CDC37L1 |
| 235740_at | 1.6087114 | MCTP1 |
| 214874_at | 1.608623 | PKP4 |
| 211919_s_at | 1.6073475 | CXCR4 |
| 205560_at | 1.6069471 | PCSK5 |
| 231929_at | 1.6063831 | IKZF2 |
| 237127_at | 1.605848 |  |
| 204864_s_at | 1.6046447 | IL6ST |
| 220399_at | 1.6046256 | LINC00115 |
| 231804_at | 1.6036023 | RXFP1 |
| 201971_s_at | 1.6034809 | ATP6V1A |
| 241404_at | 1.6024219 |  |
| 208547_at | 1.602049 | HIST1H2BB |
| 222234_s_at | 1.6013491 | DBNDD1 |
| 213424_at | 1.6006079 | KIAA0895 |
| 1552822_at | 1.6004248 | TMX3 |
| 203640_at | 1.6000847 | MBNL2 |
| 206245_s_at | 1.597525 | IVNS1ABP |
| 225567_at | 1.5974394 | LOC100505573 |
| 224525_s_at | 1.5957334 | OLA1 |
| 230465_at | 1.5957191 | HS2ST1 |
| 235512_at | 1.5952508 | CDKL1 |
| 210631_at | 1.5951723 | NF1 |
| 235704_at | 1.5943253 |  |
| 231131_at | 1.5941814 | FAM133A |
| 204472_at | 1.5916705 | GEM |
| 225438_at | 1.589772 | NUDCD1 |
| 212249_at | 1.5889497 | PIK3R1 |
| 226145_s_at | 1.5886554 | FRAS1 |
| 236423_at | 1.5880238 |  |
| 215440_s_at | 1.5879713 | BEX4 |
| 227448_at | 1.5862738 | ARGLU1 |
| 208527_x_at | 1.5861815 | HIST1H2BC///HIST1H2BE///HIST1H2BF///HIST1H2BG///HIST1H2BI |
| 222511_x_at | 1.5859529 | FAF1 |
| 228803_at | 1.5855373 | PMS1 |
| 1553743_at | 1.5847325 | METTL21A |
| 210479_s_at | 1.5846754 | RORA |
| 242762_s_at | 1.5846754 | FAM171B |
| 1561300_at | 1.5845524 |  |
| 1569788_at | 1.5845519 | ST8SIA1 |
| 236769_at | 1.5845519 | LOC158402 |
| 242292_at | 1.5836283 | FAM226A///FAM226B |
| 205945_at | 1.5819952 | IL6R |
| 238554_at | 1.5803713 | CYB5B |
| 203438_at | 1.5800385 | STC2 |
| 208579_x_at | 1.5799989 | H2BFS |
| 223310_x_at | 1.5793797 | PNPLA8 |
| 222880_at | 1.579223 | AKT3 |
| 201362_at | 1.5769275 | IVNS1ABP |
| 222457_s_at | 1.5762944 | LIMA1 |
| 219693_at | 1.5753601 | AGPAT4 |
| 205299_s_at | 1.5740963 | BTN2A2 |
| 229506_at | 1.5740963 | PPM1L |
| 242636_at | 1.5740829 | PRCP |
| 213940_s_at | 1.5740438 | FNBP1 |
| 214021_x_at | 1.5738602 | ITGB5 |
| 230630_at | 1.5738529 | AK4///LOC100507855 |
| 1563839_at | 1.5734739 | TBC1D7 |
| 230348_at | 1.5732985 | LATS2 |
| 234995_at | 1.5719101 | SPICE1 |
| 228640_at | 1.5716239 | PCDH7 |
| 1564378_a_at | 1.5712595 |  |
| 235207_at | 1.571088 | OTTHUMG00000179915///RP11-1094M14.11 |
| 1555972_s_at | 1.5710703 | FBXO28 |
| 243613_at | 1.5710113 | DENND5B |
| 232293_at | 1.5705501 | LCORL |
| 203716_s_at | 1.5703413 | DPP4 |
| 203372_s_at | 1.5698806 | SOCS2 |
| 208490_x_at | 1.5698277 | HIST1H2BC///HIST1H2BE///HIST1H2BF///HIST1H2BG///HIST1H2BI |
| 210273_at | 1.5665622 | PCDH7 |
| 226267_at | 1.5657837 | JDP2 |
| 243231_at | 1.5627158 | SLC38A11 |
| 1559510_at | 1.5620395 | LINC00630 |
| 227692_at | 1.5609988 | GNAI1 |
| 204210_s_at | 1.5602436 | PCYT1A |
| 203285_s_at | 1.5602028 | HS2ST1 |
| 217644_s_at | 1.5596322 | SOS2 |
| 1555448_at | 1.5596207 | AP5M1 |
| 1558164_s_at | 1.5595522 | PEX13 |
| 214331_at | 1.5588374 | TSFM |
| 201464_x_at | 1.5573131 | JUN |
| 239481_at | 1.5564698 | FAM133A |
| 207968_s_at | 1.5545269 | MEF2C |
| 1554882_at | 1.5542741 | ERCC8 |
| 241433_at | 1.553969 | RCOR3 |
| 236026_at | 1.5537641 | GPATCH2 |
| 205876_at | 1.5536773 | LIFR |
| 228852_at | 1.5535232 | ENSA |
| 1562309_s_at | 1.5534201 | LOC100506695///PHF21B |
| 209960_at | 1.5533015 | HGF |
| 243371_at | 1.5518934 |  |
| 223143_s_at | 1.5517876 | AKIRIN2 |
| 235115_at | 1.5512236 | PDE8B |
| 244856_at | 1.5510954 |  |
| 238536_at | 1.5507653 |  |
| 203821_at | 1.5504394 | HBEGF |
| 231431_s_at | 1.549574 | LOC100505573 |
| 235502_at | 1.547958 | PPP2CA |
| 205457_at | 1.5472404 | C6orf106 |
| 206035_at | 1.5467318 | REL |
| 1555388_s_at | 1.546637 | SNX25 |
| 217892_s_at | 1.5465194 | LIMA1 |
| 208523_x_at | 1.5464433 | HIST1H2BC///HIST1H2BE///HIST1H2BF///HIST1H2BG///HIST1H2BI |
| 237654_at | 1.5464433 | PPP1R36 |
| 1555193_a_at | 1.5463042 | ZNF277 |
| 237301_at | 1.5458643 |  |
| 227408_s_at | 1.5457509 | SNX25 |
| 211685_s_at | 1.5452533 | NCALD |
| 1555978_s_at | 1.5451272 | MYL12A |
| 235191_at | 1.5450455 | LINC00662 |
| 214290_s_at | 1.5445942 | HIST2H2AA3///HIST2H2AA4 |
| 210512_s_at | 1.5443491 | VEGFA |
| 1555865_at | 1.5434933 | TOLLIP-AS1 |
| 239702_x_at | 1.543145 | OTTHUMG00000013314///RP11-12A20.2 |
| 242577_at | 1.5431447 | LOC389834 |
| 226756_at | 1.5431031 | CCDC71L |
| 202497_x_at | 1.5428879 | SLC2A3 |
| 205515_at | 1.5427201 | PRSS12 |
| 207053_at | 1.5425987 | SLC8A1 |
| 204783_at | 1.5425677 | MLF1 |
| 221479_s_at | 1.5419763 | BNIP3L |
| 236235_at | 1.5418732 | ITCH |
| 222576_s_at | 1.5418724 | AGO1 |
| 215071_s_at | 1.5416992 | HIST1H2AC |
| 211020_at | 1.5414872 | GCNT2 |
| 209288_s_at | 1.5410796 | CDC42EP3 |
| 1564190_x_at | 1.5400103 | ZNF519 |
| 213913_s_at | 1.5394913 | TBC1D30 |
| 203320_at | 1.5392948 | SH2B3 |
| 1554614_a_at | 1.5386295 | PTBP2 |
| 232184_at | 1.5379884 | ALS2 |
| 210447_at | 1.5378618 | GLUD2 |
| 237553_at | 1.5367653 | LZTFL1 |
| 223791_at | 1.5367644 | FAM27A///FAM27B///FAM27C |
| 238360_s_at | 1.5367135 | LINC00672 |
| 237145_at | 1.5366409 | EIF2AK4 |
| 204093_at | 1.5358584 | CCNH |
| 232810_at | 1.5357397 | AIG1 |
| 237622_at | 1.5357392 |  |
| 228987_at | 1.5356452 | FAM49B |
| 1552485_at | 1.5354859 | LACTB |
| 217451_at | 1.5343024 |  |
| 1555598_a_at | 1.534025 | DUSP19 |
| 1555976_s_at | 1.5340112 | MYL12A |
| 235635_at | 1.5328699 | ARHGAP5 |
| 38037_at | 1.5323497 | HBEGF |
| 240801_at | 1.5320003 | C21orf37 |
| 220122_at | 1.5319405 | MCTP1 |
| 242826_at | 1.5303018 |  |
| 235226_at | 1.5285013 | CDK19 |
| 202768_at | 1.5284038 | FOSB |
| 236982_at | 1.527264 |  |
| 212839_s_at | 1.5272638 | TROVE2 |
| 206319_s_at | 1.5272633 | EPPIN///EPPIN-WFDC6 |
| 212425_at | 1.5269865 | SCAMP1 |
| 201363_s_at | 1.5269768 | IVNS1ABP |
| 219941_at | 1.526551 | TMEM19 |
| 213452_at | 1.525933 | ZNF184 |
| 210875_s_at | 1.525697 | LOC100996668///ZEB1 |
| 227020_at | 1.5256597 | YPEL2 |
| 205280_at | 1.5242015 | GLRB |
| 208115_x_at | 1.5239346 | C10orf137 |
| 241612_at | 1.523926 | FOXD3 |
| 216048_s_at | 1.5236632 | RHOBTB3 |
| 221582_at | 1.5236268 | HIST3H2A |
| 1557051_s_at | 1.523509 | HOTAIRM1 |
| 200779_at | 1.5234617 | ATF4 |
| 1555888_at | 1.523281 | KB-431C1.4///OTTHUMG00000164878 |
| 225224_at | 1.5230634 | C20orf112 |
| 215012_at | 1.5225214 | ZNF451 |
| 238003_at | 1.5217487 | HEPACAM///HEPN1 |
| 1555928_at | 1.5217481 |  |
| 241420_at | 1.521208 |  |
| 228193_s_at | 1.5207415 | RGCC |
| 202856_s_at | 1.5197883 | SLC16A3 |
| 205759_s_at | 1.5193654 | SULT2B1 |
| 219033_at | 1.519256 | PARP8 |
| 219320_at | 1.5184909 | MYO19 |
| 223682_s_at | 1.517819 | EIF1AD |
| 213949_s_at | 1.5174308 | DOHH |
| 211813_x_at | 1.5173997 | DCN |
| 224839_s_at | 1.5172547 | GPT2 |
| 232576_at | 1.5171574 |  |
| 228919_at | 1.5165964 |  |
| 50374_at | 1.5163723 | OXLD1 |
| 214231_s_at | 1.5161885 | VWA8 |
| 239081_at | 1.5161879 |  |
| 214319_at | 1.515577 | FRY |
| 1559116_s_at | 1.5154294 |  |
| 224566_at | 1.514403 | MIR612///NEAT1 |
| 203729_at | 1.5138966 | EMP3 |
| 236439_at | 1.5136074 |  |
| 228499_at | 1.5134717 | PFKFB4 |
| 208944_at | 1.5133452 | TGFBR2 |
| 214691_x_at | 1.5124786 | FAM63B |
| 238787_at | 1.5123087 | DENND1B |
| 217280_x_at | 1.5118784 | GABRA5 |
| 200697_at | 1.5113767 | HK1 |
| 214139_at | 1.5108172 | ARID4B |
| 217234_s_at | 1.5107695 | EZR |
| 210299_s_at | 1.5106986 | FHL1 |
| 1554655_a_at | 1.5105727 | RPRML |
| 223431_at | 1.5105478 | BLOC1S4 |
| 206721_at | 1.5102162 | CCDC181 |
| 242656_at | 1.5098869 | GTF2H1 |
| 1554493_s_at | 1.5091724 | THADA |
| 226660_at | 1.5090407 | RPS6KB1 |
| 1557609_s_at | 1.5090128 | TBC1D12 |
| 227440_at | 1.5088388 | ANKS1B |
| 216049_at | 1.5084388 | RHOBTB3 |
| 235012_at | 1.5083776 | LRCH1 |
| 244660_at | 1.5082693 | ELAVL1 |
| 200920_s_at | 1.5081013 | BTG1 |
| 227014_at | 1.5078058 | ASPHD2 |
| 239140_at | 1.5072811 |  |
| 230954_at | 1.5072607 | C20orf112 |
| 220361_at | 1.5064628 | IQCH |
| 1560220_a_at | 1.5063518 | CBY1 |
| 238438_at | 1.5063508 | CNOT6L |
| 1560739_a_at | 1.5060703 |  |
| 1570263_at | 1.50607 |  |
| 235394_at | 1.5055106 | PLAA |
| 227339_at | 1.504759 | RGMB |
| 219050_s_at | 1.5047038 | ZNHIT2 |
| 214522_x_at | 1.5041085 | HIST1H2AD///HIST1H3A///HIST1H3B///HIST1H3C///HIST1H3D  ///HIST1H3E///HIST1H3F///HIST1H3G///HIST1H3H///HIST1H3I///  HIST1H3J |
| 210636_at | 1.5037233 | PPARD |
| 214472_at | 1.503604 | HIST1H2AD///HIST1H3A///HIST1H3B///HIST1H3C///HIST1H3D  ///HIST1H3E///HIST1H3F///HIST1H3G///HIST1H3H///HIST1H3I///  HIST1H3J |
| 244030_at | 1.5035851 | STYX |
| 204769_s_at | 1.5035205 | TAP2 |
| 1553685_s_at | 1.5034072 | SP1 |
| 244049_at | 1.5032026 |  |
| 202140_s_at | 1.5024873 | CLK3 |
| 204286_s_at | 1.502441 | PMAIP1 |
| 228081_at | 1.5023303 | CCNG2 |
| 229568_at | 1.5014517 | MOB3B |
| 229829_at | 1.5014135 | LINC00526 |
| 209598_at | 1.501287 | PNMA2 |
| 201925_s_at | 1.5010935 | CD55 |
| 225282_at | 1.5009446 | SMAP2 |
| 1559258_a_at | 1.4994521 | CXorf61 |
| 220462_at | 1.4993728 | CSRNP3 |
| 219713_at | 1.4992826 | SHPK |
| 240355_at | 1.499255 |  |
| 210021_s_at | 1.4992485 | CCNO |
| 201962_s_at | 1.4987901 | RNF41 |
| 226012_at | 1.4987853 | ANKRD11 |
| 225799_at | 1.4985534 | LINC00152///LOC541471 |
| 1555908_at | 1.4984053 | FAM120A |
| 219638_at | 1.4983518 | FBXO22 |
| 234397_at | 1.4983518 | ZNF861P///ZNF861P |
| 229141_at | 1.4983104 | SFT2D3///WDR33 |
| 215990_s_at | 1.4982959 | BCL6 |
| 214169_at | 1.4982344 | SUN1 |
| 230847_at | 1.4978853 | WRNIP1 |
| 224503_s_at | 1.4977572 | ZCCHC2 |
| 203294_s_at | 1.4975551 | LMAN1 |
| 203946_s_at | 1.4971305 | ARG2 |
| 228457_at | 1.4968456 | PPM1L |
| 224147_at | 1.4959434 | PARPBP |
| 216202_s_at | 1.4958446 | SPTLC2 |
| 222671_s_at | 1.495756 | JMJD4 |
| 240027_at | 1.4954456 | LIN7A |
| 221448_s_at | 1.494989 | TEX15 |
| 207723_s_at | 1.4945177 | KLRC3 |
| 224898_at | 1.494289 | WDR26 |
| 209339_at | 1.494128 | SIAH2 |
| 221220_s_at | 1.4939749 | SCYL2 |
| 220133_at | 1.4939744 | ODAM |
| 209841_s_at | 1.493974 | LRRN3 |
| 209198_s_at | 1.4938357 | SYT11 |
| 213035_at | 1.4936895 | ANKRD28 |
| 202551_s_at | 1.4936289 | CRIM1 |
| 214247_s_at | 1.493571 | DKK3 |
| 1552314_a_at | 1.4930972 | EYA3 |
| 230795_at | 1.4927365 |  |
| 238917_s_at | 1.4919542 | DENND5B |
| 225582_at | 1.491829 | ITPRIP |
| 209890_at | 1.4915311 | TSPAN5 |
| 222612_at | 1.4913986 | PSPC1 |
| 210493_s_at | 1.4912813 | MFAP3L |
| 222347_at | 1.4912173 |  |
| 238139_at | 1.4910021 | OTTHUMG00000175777///RP5-968P14.2 |
| 221530_s_at | 1.490687 | BHLHE41 |
| 218704_at | 1.4905766 | RNF43 |
| 243376_at | 1.4903241 | TANK |
| 215766_at | 1.4900899 | GSTA1 |
| 1560434_x_at | 1.4874308 | CLTA |
| 211205_x_at | 1.4872662 | PIP5K1A |
| 238427_at | 1.4869903 | GRPEL2 |
| 223666_at | 1.4863752 | SNX5 |
| 235599_at | 1.4858725 | LOC339535 |
| 225919_s_at | 1.4857612 | C9orf72 |
| 213664_at | 1.4851396 | SLC1A1 |
| 203217_s_at | 1.4850812 | ST3GAL5 |
| 236202_at | 1.4849669 |  |
| 218651_s_at | 1.4838786 | LARP6 |
| 206125_s_at | 1.4837364 | KLK8 |
| 213107_at | 1.4832015 | TNIK |
| 221016_s_at | 1.4831407 | TCF7L1 |
| 204900_x_at | 1.4822503 | SAP30 |
| 205574_x_at | 1.4820739 | BMP1 |
| 230291_s_at | 1.4820739 |  |
| 224508_at | 1.4820054 | MGC12916 |
| 226652_at | 1.4816825 | USP3 |
| 215285_s_at | 1.4805998 | PHTF1 |
| 1565602_at | 1.4805444 |  |
| 230887_at | 1.480379 | CDC14B |
| 217862_at | 1.4797025 | PIAS1 |
| 206879_s_at | 1.4793372 | NRG2 |
| 206748_s_at | 1.478408 | SPAG9 |
| 202259_s_at | 1.4782087 | N4BP2L2 |
| 238681_at | 1.4770155 | GDPD1 |
| 217551_at | 1.4768249 | OR7E14P |
| 1564287_at | 1.476791 | LINC00410 |
| 239536_at | 1.4766997 |  |
| 209905_at | 1.4765797 | HOXA10-HOXA9///HOXA9///MIR196B |
| 220773_s_at | 1.4765592 | GPHN |
| 216856_s_at | 1.476417 |  |
| 233050_at | 1.4762864 | KIAA0408///SOGA3 |
| 244806_at | 1.4762825 |  |
| 202034_x_at | 1.4759985 | RB1CC1 |
| 1562544_at | 1.4757819 |  |
| 204525_at | 1.4757819 | PHF14 |
| 222755_s_at | 1.4757819 | CHD7 |
| 211032_at | 1.475781 | COBLL1 |
| 237850_at | 1.475781 |  |
| 228964_at | 1.4752314 | PRDM1 |
| 235061_at | 1.4751918 | PPM1K |
| 203641_s_at | 1.4751694 | COBLL1 |
| 242450_at | 1.4749588 | RGMB |
| 1568627_at | 1.4749042 | SMEK2 |
| 1569909_at | 1.4748696 | KRT79 |
| 236207_at | 1.4748694 | SSFA2 |
| 202873_at | 1.4748691 | ATP6V1C1 |
| 224241_s_at | 1.4748126 |  |
| 1568733_at | 1.4744773 | C10orf76 |
| 228036_s_at | 1.4738594 | FBXO2 |
| 225206_s_at | 1.4736607 | MTRF1L |
| 201540_at | 1.47354 | FHL1 |
| 236462_at | 1.4734987 |  |
| 1568648_a_at | 1.473498 | LOC100505851 |
| 236083_at | 1.473498 | BCL2L15 |
| 215779_s_at | 1.4733305 | HIST1H2BC///HIST1H2BE///HIST1H2BF///HIST1H2BG///  HIST1H2BI///NCALD |
| 1564232_at | 1.4729857 | LOC441528 |
| 237817_at | 1.4729804 | SSR3 |
| 231822_at | 1.472903 | CTTNBP2NL |
| 222946_s_at | 1.472775 | AUNIP |
| 214613_at | 1.4726197 | GPR3 |
| 1559922_at | 1.4724591 |  |
| 220031_at | 1.4724504 | OTUD7B |
| 237675_at | 1.472172 | OTTHUMG00000035535///RP11-547I7.2 |
| 221904_at | 1.4717774 | FAM131A |
| 218793_s_at | 1.4714618 | SCML1 |
| 210138_at | 1.4714394 | RGS20 |
| 1564166_s_at | 1.470889 | LOC100630923 |
| 223309_x_at | 1.470555 | PNPLA8 |
| 228658_at | 1.4700431 | MIAT |
| 221546_at | 1.4700423 | PRPF18 |
| 213929_at | 1.4694433 | EXPH5 |
| 244148_at | 1.468733 |  |
| 213562_s_at | 1.4676125 | SQLE |
| 1559827_at | 1.4671415 | LINC00960 |
| 202284_s_at | 1.466727 | CDKN1A |
| 1558660_at | 1.4664406 | LINC00703 |
| 243389_at | 1.4661759 | PRH1///PRH1-PRR4///PRR4 |
| 242141_at | 1.4661052 | HDAC2 |
| 202949_s_at | 1.4657767 | FHL2 |
| 1554780_a_at | 1.4656652 | PHTF2 |
| 243679_at | 1.4656405 | JPH3 |
| 235278_at | 1.4655015 | MACROD2 |
| 236937_at | 1.465224 | LOC100505729///VPS8 |
| 210232_at | 1.4651285 | CDC42 |
| 210788_s_at | 1.4649949 | DHRS7 |
| 1557354_at | 1.464779 | SOS1 |
| 219345_at | 1.4640404 | BOLA1 |
| 224920_x_at | 1.4640268 | MYADM |
| 1570243_at | 1.4636244 |  |
| 224407_s_at | 1.4633844 | MST4 |
| 1558779_at | 1.4631658 | CTC-203F4.2///OTTHUMG00000171136 |
| 228169_s_at | 1.4631658 |  |
| 242778_at | 1.4631194 | LPXN |
| 214942_at | 1.4625084 | RBM34 |
| 224483_s_at | 1.4624386 | MFSD9 |
| 1565406_a_at | 1.4624261 | LHX9 |
| 220669_at | 1.4624261 | OTUD4 |
| 241437_s_at | 1.4619089 | EP400NL |
| 201549_x_at | 1.4612191 | KDM5B |
| 1556301_at | 1.4608995 | LOC100287015 |
| 228778_at | 1.4597859 | MCPH1 |
| 229114_at | 1.4597826 | GAB1 |
| 210971_s_at | 1.459516 | ARNTL |
| 235407_at | 1.4595003 |  |
| 210567_s_at | 1.4594694 | SKP2 |
| 209599_s_at | 1.4593995 | PRUNE |
| 205748_s_at | 1.4589119 | RNF126 |
| 244881_at | 1.4587234 | LMLN |
| 222222_s_at | 1.458639 | HOMER3 |
| 209008_x_at | 1.4585662 | KRT8 |
| 228562_at | 1.4584472 | ZBTB10 |
| 204137_at | 1.4579136 | GPR137B |
| 221950_at | 1.4573952 | EMX2 |
| 1560981_a_at | 1.4573687 | PPARA |
| 204754_at | 1.4573683 | HLF |
| 224976_at | 1.4572902 | NFIA |
| 1560007_at | 1.4572821 | LOC645984 |
| 209102_s_at | 1.4571525 | HBP1 |
| 212501_at | 1.456642 | CEBPB |
| 239894_at | 1.4556335 | NEBL-AS1 |
| 209448_at | 1.4554889 | HTATIP2 |
| 1559534_at | 1.4549373 | CTC-241N9.1///OTTHUMG00000163262 |
| 233545_at | 1.4549373 |  |
| 229160_at | 1.4549369 | MUM1L1 |
| 240382_at | 1.454209 | OTTHUMG00000175897///RP3-512B11.3 |
| 219168_s_at | 1.4539763 | PRR5 |
| 242488_at | 1.4534853 | CHRM3 |
| 208848_at | 1.4534603 | ADH5 |
| 225660_at | 1.4529108 | SEMA6A |
| 216899_s_at | 1.4528455 | SKAP2 |
| 219696_at | 1.452688 | DENND1B |
| 211021_s_at | 1.4526266 | RGS14 |
| 223651_x_at | 1.4522276 | CDC23 |
| 244841_at | 1.4521167 | SEC24A |
| 224476_s_at | 1.4519469 | MESP1 |
| 221511_x_at | 1.4519258 | CCPG1///DYX1C1-CCPG1 |
| 212840_at | 1.4511601 | UBXN7 |
| 236781_at | 1.4507649 |  |
| 244680_at | 1.4507647 | GLRB |
| 1554757_a_at | 1.4496855 | INPP5A |
| 1558163_at | 1.4496855 | PEX13 |
| 207543_s_at | 1.4492984 | P4HA1 |
| 202363_at | 1.4490435 | SPOCK1 |
| 1555559_s_at | 1.4490215 | USP25 |
| 222747_s_at | 1.4488567 | SCML1 |
| 229417_at | 1.4484326 |  |
| 244758_at | 1.4483578 | SCAND3 |
| 207083_s_at | 1.4482381 | CC2D1A |
| 238206_at | 1.4482381 | RXFP1 |
| 221338_at | 1.4479312 | LY6G6E |
| 236996_at | 1.4479307 |  |
| 206440_at | 1.4477777 | LIN7A |
| 1569495_at | 1.4473075 | SCLT1 |
| 232099_at | 1.4473075 | PCDHB16 |
| 238065_at | 1.4468995 | TPM3 |
| 1554415_at | 1.4466202 | TAF5L |
| 214827_at | 1.4465375 | PARD6B |
| 228441_s_at | 1.4465103 | OTTHUMG00000161676///RP11-164P12.5 |
| 1554980_a_at | 1.4460657 | ATF3 |
| 218603_at | 1.4459089 | HECA |
| 235861_at | 1.4456433 |  |
| 1556227_at | 1.4449191 | VCPIP1 |
| 1554575_a_at | 1.4449186 | BPNT1 |
| 205686_s_at | 1.444773 | CD86 |
| 231028_at | 1.4447443 | LOC100506082 |
| 239295_at | 1.4446459 | SRSF12 |
| 200921_s_at | 1.4444625 | BTG1 |
| 209398_at | 1.4433258 | HIST1H1C |
| 242433_at | 1.4432714 | ZBTB11 |
| 241652_x_at | 1.4432461 | LIN7A |
| 214326_x_at | 1.4429603 | JUND |
| 230302_at | 1.4429603 | OTTHUMG00000174444///RP11-48B3.4 |
| 232103_at | 1.4426914 | BPNT1 |
| 229329_s_at | 1.4422891 |  |
| 226881_at | 1.4422567 | GRPEL2 |
| 242263_at | 1.4421837 | TMED5 |
| 220156_at | 1.4418156 | EFCAB1 |
| 221456_at | 1.4418154 | TAS2R3 |
| 1553521_at | 1.4418151 | DEFB104A///DEFB104B |
| 213926_s_at | 1.441742 | AGFG1 |
| 243067_at | 1.4417019 |  |
| 202681_at | 1.4416057 | C3orf62///MIR4271///USP4 |
| 213056_at | 1.4415809 | FRMD4B |
| 226615_at | 1.4415213 | XPR1 |
| 220444_at | 1.4412303 | ZNF557 |
| 1556064_at | 1.4411879 | LOC284926 |
| 214446_at | 1.4411874 | ELL2 |
| 237939_at | 1.4411874 | EPHA5 |
| 223167_s_at | 1.4411836 | USP25 |
| 221731_x_at | 1.4407321 | VCAN |
| 242462_at | 1.4405054 | LINC00665 |
| 1557329_at | 1.4402591 |  |
| 1570168_at | 1.4402591 |  |
| 1563494_at | 1.4402589 |  |
| 1554237_at | 1.4402584 | SDCCAG8 |
| 233781_s_at | 1.4402579 | RIF1 |
| 235179_at | 1.439641 | ZNF641 |
| 235009_at | 1.439465 | BOD1L1 |
| 216247_at | 1.4394641 | RPS20///SNORD54 |
| 235184_at | 1.4391558 | AEBP2 |
| 225955_at | 1.4387097 | METRNL |
| 216870_x_at | 1.4384472 | DLEU2 |
| 1554043_a_at | 1.4381558 |  |
| 1553105_s_at | 1.4381487 | DSG2 |
| 242224_at | 1.4379234 | GPATCH2 |
| 214023_x_at | 1.4377486 | TUBB2B |
| 222756_s_at | 1.4375993 | ARRB1 |
| 226543_at | 1.4370663 | BLOC1S5///EEF1E1-MUTED |
| 236298_at | 1.4370549 | PDSS1 |
| 1554140_at | 1.4370544 | WDR78 |
| 214188_at | 1.4370468 |  |
| 1558512_at | 1.4370008 | OTTHUMG00000172986///RP11-819C21.1 |
| 1557050_at | 1.4369808 | HOTAIRM1 |
| 220240_s_at | 1.4365842 | TMCO3 |
| 222655_s_at | 1.4362291 | IMPAD1 |
| 225215_s_at | 1.4360027 | MTRF1L |
| 1556429_a_at | 1.4359405 | WDR67 |
| 241432_at | 1.4358084 | SLIT2-IT1 |
| 243141_at | 1.4358082 | SGMS2 |
| 57703_at | 1.4357375 | SENP5 |
| 220468_at | 1.4344828 | ARL14 |
| 1564630_at | 1.4341453 | EDN1 |
| 208570_at | 1.4341453 | WNT1 |
| 215182_x_at | 1.4341453 |  |
| 228148_at | 1.4340941 | ZNF584 |
| 202157_s_at | 1.4335917 | CELF2 |
| 228153_at | 1.4333028 | RNF144B |
| 207031_at | 1.4332099 | NKX3-2 |
| 236935_at | 1.4328973 | PTPN4 |
| 226172_at | 1.432416 | USP42 |
| 228493_at | 1.4323432 | OTTHUMG00000022286///RP3-327A19.5 |
| 1562572_at | 1.4320801 |  |
| 1557405_at | 1.4320327 | LOC100130111 |
| 1558984_at | 1.4320327 | MAP3K11 |
| 241250_at | 1.4320325 |  |
| 235216_at | 1.4318823 | ESCO1 |
| 206667_s_at | 1.4318008 | SCAMP1 |
| 235152_at | 1.4317223 | AP001258.4///OTTHUMG00000167435 |
| 222509_s_at | 1.4310524 | ZNF672 |
| 215185_at | 1.43104 | LINC00963 |
| 203377_s_at | 1.4309791 | CDC40 |
| 230643_at | 1.4307611 | WNT9A |
| 239950_at | 1.4307213 | HOXA11-AS |
| 229810_at | 1.4306036 |  |
| 218145_at | 1.4304603 | TRIB3 |
| 233230_s_at | 1.4299432 | SLAIN2 |
| 227688_at | 1.4269339 | LRCH2 |
| 205732_s_at | 1.4265057 | NCOA2 |
| 231966_at | 1.4264666 | PPP1R9A |
| 1558254_s_at | 1.4264567 | SRPK2 |
| 231955_s_at | 1.4262794 | HIBADH |
| 204529_s_at | 1.4259475 | TOX |
| 215229_at | 1.4259118 | LOC100129973 |
| 241866_at | 1.4259118 | SLC16A7 |
| 216903_s_at | 1.4254273 | MICU1 |
| 242938_s_at | 1.424718 | FOXK2 |
| 201192_s_at | 1.4244063 | PITPNA |
| 210690_at | 1.4241596 | KLRC4 |
| 224100_s_at | 1.4238 | DPYSL5 |
| 212930_at | 1.4237347 | ATP2B1 |
| 239343_at | 1.4237342 | LOC728705 |
| 213960_at | 1.4233618 | NTRK3 |
| 213986_s_at | 1.4233615 | TMEM259 |
| 239439_at | 1.4233154 | AFF4 |
| 220334_at | 1.4231771 | RGS17 |
| 1554154_at | 1.4231677 | GDAP2 |
| 209199_s_at | 1.4227765 | MEF2C |
| 227295_at | 1.4224317 | IKBIP |
| 205726_at | 1.4223777 | DIAPH2 |
| 204530_s_at | 1.4222611 | TOX |
| 215019_x_at | 1.4222611 | ZNF528 |
| 1560431_at | 1.4222606 | OTTHUMG00000066821///RP11-87H9.3 |
| 1555932_at | 1.4222604 | TSGA10 |
| 239015_at | 1.422121 | THAP7-AS1 |
| 1569355_at | 1.4220744 | KCNIP3 |
| 226559_at | 1.4218451 | IER5L |
| 1557617_at | 1.4218061 | DCTN1-AS1 |
| 236302_at | 1.4214997 | PPM1E |
| 1554264_at | 1.4213213 | CKAP2 |
| 201279_s_at | 1.4212339 | DAB2 |
| 223484_at | 1.4210047 | C15orf48 |
| 221797_at | 1.4201943 | OXLD1 |
| 227708_at | 1.4200553 | EEF1A1 |
| 206042_x_at | 1.4197887 | PAR-SN///SNORD107///SNRPN///SNURF |
| 208886_at | 1.4196593 | H1F0 |
| 235952_at | 1.4193228 |  |
| 225864_at | 1.4193102 | FAM84B |
| 1554307_at | 1.4185867 | LOC644852 |
| 224496_s_at | 1.4185302 | TMEM107 |
| 231967_at | 1.4182386 | PHF20L1 |
| 206315_at | 1.4179438 | CRLF1 |
| 221676_s_at | 1.4175643 | CORO1C |
| 33323_r_at | 1.4175189 | SFN |
| 229830_at | 1.4172654 |  |
| 234946_at | 1.4170429 | ENTPD6 |
| 239229_at | 1.4170423 | PHEX |
| 1555112_a_at | 1.4166734 | CCDC181 |
| 240028_at | 1.4164271 | FSIP2 |
| 226865_at | 1.4161285 | LOC100509635 |
| 1560069_at | 1.4159667 | PLEKHM3 |
| 219499_at | 1.4157485 | SEC61A2 |
| 200760_s_at | 1.4152591 | ARL6IP5 |
| 219858_s_at | 1.414924 | MFSD6 |
| 220513_at | 1.414924 | KHDC1L |
| 202686_s_at | 1.4145758 | AXL |
| 1552329_at | 1.4141201 | RBBP6 |
| 220860_at | 1.4140733 | PURG |
| 1557388_at | 1.4139453 | RTTN |
| 1557402_at | 1.413624 | OTTHUMG00000175907///RP1-265C24.8 |
| 215393_s_at | 1.4135388 | COBLL1 |
| 1561449_at | 1.4131837 |  |
| 235270_at | 1.413179 | ZNF397 |
| 210950_s_at | 1.4128867 | FDFT1 |
| 207050_at | 1.4125692 | CACNA2D1 |
| 210458_s_at | 1.4123815 | TANK |
| 202712_s_at | 1.4123143 | CKMT1A///CKMT1B |
| 243927_x_at | 1.4120702 | KIAA1429 |
| 220220_at | 1.4112933 | LRRC37A4P |
| 243622_at | 1.4112933 | LOC145694 |
| 1556809_a_at | 1.411293 | RAP2A |
| 207156_at | 1.4111245 | HIST1H2AG///HIST1H2AH///HIST1H2AI///HIST1H2AK///  HIST1H2AL///HIST1H2AM |
| 226974_at | 1.4110265 | NEDD4L |
| 211651_s_at | 1.4109794 | LAMB1 |
| 237694_at | 1.4108691 |  |
| 241840_at | 1.4107691 |  |
| 203330_s_at | 1.4107682 | STX5 |
| 236788_at | 1.4107682 | OTTHUMG00000018082///OTTHUMG00000174847///RP11-67C2.2 |
| 1555318_at | 1.4107677 | HIF3A |
| 229603_at | 1.4107672 | BBS12 |
| 1569283_at | 1.4101682 | ZNF891 |
| 228702_at | 1.4097912 | FLJ43663 |
| 215342_s_at | 1.4095433 | RABGAP1L |
| 206796_at | 1.409543 | WISP1 |
| 231740_at | 1.409543 | KCNJ11 |
| 242702_at | 1.4095426 | MMAA |
| 218107_at | 1.4088186 | WDR26 |
| 235456_at | 1.4088056 |  |
| 214849_at | 1.4086002 | KCTD20 |
| 76897_s_at | 1.4085296 | FKBP15 |
| 204821_at | 1.4082935 | BTN3A3 |
| 222562_s_at | 1.4081677 | TNKS2 |
| 238067_at | 1.4079872 | TBC1D8B |
| 232296_s_at | 1.4078797 | GFM1 |
| 231270_at | 1.4076196 | CA13///LOC100507258 |
| 230738_at | 1.4074382 | OTTHUMG00000178927///RP11-196G18.23 |
| 212387_at | 1.4072586 | TCF4 |
| 215294_s_at | 1.4067713 | SMARCA1 |
| 1553112_s_at | 1.4062315 | CDK8 |
| 1552269_at | 1.4061646 | SPATA17 |
| 201208_s_at | 1.4057078 | TNFAIP1 |
| 202921_s_at | 1.4054123 | ANK2 |
| 1570125_at | 1.4052907 |  |
| 213268_at | 1.4052907 | CAMTA1 |
| 238861_at | 1.4050868 | SSBP2 |
| 231664_at | 1.4050863 | SLC25A29 |
| 238529_at | 1.4050139 | OTTHUMG00000178927///RP11-196G18.23 |
| 1561693_at | 1.4050101 | LOC400794 |
| 1559883_s_at | 1.4049025 | SAMHD1 |
| 239433_at | 1.4048402 | LRRC8E |
| 243707_at | 1.4048104 |  |
| 222156_x_at | 1.4047863 | CCPG1///DYX1C1-CCPG1 |
| 1554660_a_at | 1.4044623 | CNST |
| 236698_at | 1.4044623 | DYNC1I2 |
| 1553959_a_at | 1.4043406 | B3GALT6 |
| 228297_at | 1.4042312 |  |
| 234067_at | 1.4039581 |  |
| 244870_at | 1.4037902 | TES |
| 1552531_a_at | 1.4036186 | NLRP11 |
| 1552508_at | 1.4036182 | KCNE4 |
| 219872_at | 1.403608 | FAM198B |
| 218274_s_at | 1.4036047 | ANKZF1 |
| 1559324_at | 1.4035695 | USP32P2 |
| 206686_at | 1.4035695 | PDK1 |
| 208071_s_at | 1.4035695 | LAIR1 |
| 227206_at | 1.4035667 | NDUFA10 |
| 211911_x_at | 1.4033885 | HLA-B |
| 1556078_at | 1.4032339 |  |
| 236173_s_at | 1.4032339 | LRIG1 |
| 1556233_s_at | 1.4032334 | KIF6 |
| 240110_at | 1.4032334 | HMGCS2 |
| 222791_at | 1.403229 | RSBN1 |
| 1560485_at | 1.403101 | HIVEP1 |
| 228412_at | 1.403101 | LOC643072 |
| 224519_at | 1.4031005 | LOC100132167 |
| 202669_s_at | 1.4027709 | EFNB2 |
| 213679_at | 1.4027709 | TTC30A |
| 229744_at | 1.4027542 | SSFA2 |
| 227585_at | 1.4026517 | ATAD1 |
| 202364_at | 1.4024926 | MXI1 |
| 205548_s_at | 1.4022869 | BTG3 |
| 1553175_s_at | 1.4019388 | PDE5A |
| 220400_at | 1.4018373 | VPS13B |
| 242884_at | 1.4018373 | LOC440570 |
| 1561962_at | 1.4018371 |  |
| 230106_at | 1.4018369 | ZXDC |
| 233139_at | 1.4018369 |  |
| 235395_at | 1.4018366 | SEC63 |
| 236358_at | 1.4018115 |  |
| 1553037_a_at | 1.4017345 | SYN2 |
| 231856_at | 1.401502 | KIAA1244 |
| 243011_at | 1.401502 | NXPE3 |
| 1554766_s_at | 1.4013547 |  |
| 212764_at | 1.401326 | LOC100996668///ZEB1 |
| 212634_at | 1.4012741 | UFL1 |
| 220742_s_at | 1.4011046 | NGLY1 |
| 241453_at | 1.4007934 | PTK2 |
| 221177_at | 1.400487 | MIA2 |
| 228747_at | 1.4004388 | SEC61A2 |
| 231172_at | 1.4002999 | C9orf117 |
| 220800_s_at | 1.4002259 | TMOD3 |
| 235626_at | 1.4000343 | CAMK1D |
| 220011_at | 1.3995013 | AUNIP |
| 1566727_at | 1.3992105 |  |
| 204110_at | 1.3989836 | HNMT |
| 239252_at | 1.3989704 | COX7B |
| 205268_s_at | 1.3988276 | ADD2 |
| 1558906_a_at | 1.3987827 | OSER1-AS1 |
| 224453_s_at | 1.3987485 | ETNK1 |
| 243880_at | 1.398619 | GOSR2 |
| 207439_s_at | 1.3985617 | SLC35A2 |
| 229872_s_at | 1.3983177 | LOC100132999///LOC100996473///LOC100996720///LOC100996740///LOC101060404///LOC101060562///LOC101060645///LOC101060698///LOC642441///LOC730256 |
| 237271_at | 1.3980037 | LOC154872 |
| 236093_at | 1.3979508 | CTD-3138B18.5///OTTHUMG00000183460 |
| 227794_at | 1.3979087 | GLYATL1///LOC100287413 |
| 230680_at | 1.3979084 | SLITRK4 |
| 209160_at | 1.3979082 | AKR1C3 |
| 224099_at | 1.3977445 | KCNH7 |
| 223630_at | 1.3976251 | C7orf13 |
| 209086_x_at | 1.3975313 | MCAM |
| 235749_at | 1.3975091 | UGGT2 |
| 201037_at | 1.3973346 | PFKP |
| 201170_s_at | 1.3970556 | BHLHE40 |
| 206223_at | 1.3969716 | LMTK2 |
| 214960_at | 1.3968321 | API5 |
| 1553148_a_at | 1.3966899 | SNX13 |
| 230141_at | 1.3961952 | ARID4A |
| 201766_at | 1.396077 | ELAC2 |
| 230604_at | 1.3958191 |  |
| 226535_at | 1.395707 | ITGB6 |
| 235000_at | 1.3956097 | LPP |
| 208967_s_at | 1.3950641 | AK2 |
| 218724_s_at | 1.3949963 | TGIF2 |
| 240530_at | 1.3948336 |  |
| 217556_at | 1.3945565 | CLCN4 |
| 214277_at | 1.3941966 | COX11 |
| 207604_s_at | 1.3941137 | SLC4A7 |
| 210465_s_at | 1.3939805 | SNAPC3 |
| 223825_at | 1.3938695 | KIAA1432 |
| 218094_s_at | 1.3937663 | DBNDD2///SYS1///SYS1-DBNDD2 |
| 1555819_s_at | 1.3936917 | SAMD14 |
| 223976_at | 1.3936917 | FUT10 |
| 241820_at | 1.3936452 | RIF1 |
| 204037_at | 1.3934733 | LPAR1 |
| 50221_at | 1.3933425 | TFEB |
| 201445_at | 1.3927956 | CNN3 |
| 213292_s_at | 1.3922027 | SNX13 |
| 228820_at | 1.3921636 | XPNPEP3 |
| 231141_at | 1.3920541 | OTTHUMG00000172885///RP11-217B1.2 |
| 221088_s_at | 1.3920311 | PPP1R9A |
| 212646_at | 1.3920306 | RFTN1 |
| 211202_s_at | 1.3916948 | KDM5B |
| 226208_at | 1.3913214 | ZSWIM6 |
| 1567032_s_at | 1.3911705 | ZNF160 |
| 1553726_s_at | 1.3910638 | C6orf170 |
| 205778_at | 1.3903762 | KLK7 |
| 201275_at | 1.3903303 | FDPS |
| 230715_at | 1.3900528 | ZNF518B |
| 1568803_at | 1.3900523 |  |
| 209010_s_at | 1.3895036 | TRIO |
| 236655_at | 1.3894184 | TPD52 |
| 217097_s_at | 1.3893913 | PHTF2 |
| 218656_s_at | 1.3892614 | LHFP |
| 218723_s_at | 1.3891186 | RGCC |
| 212998_x_at | 1.3891158 | HLA-DQB1///LOC101060835 |
| 218789_s_at | 1.3889762 | C11orf71 |
| 233708_at | 1.3889464 |  |
| 227534_at | 1.3885833 | AAED1 |
| 238719_at | 1.388531 | PPP2CA |
| 202213_s_at | 1.3884714 | CUL4B |
| 207301_at | 1.3884557 | EFNA5 |
| 237278_x_at | 1.3884555 |  |
| 1554918_a_at | 1.3881377 | ABCC4 |
| 205921_s_at | 1.3881371 | SLC6A6 |
| 217841_s_at | 1.3881322 | PPME1 |
| 229071_at | 1.3880332 | C17orf100 |
| 222590_s_at | 1.3878455 | NLK |
| 1561320_at | 1.3877217 | OTTHUMG00000176890///RP11-133K1.8 |
| 225688_s_at | 1.3876524 | PHLDB2 |
| 228032_s_at | 1.3874639 | DENND1B |
| 230307_at | 1.3873897 | SLC25A21-AS1 |
| 217863_at | 1.3872951 | PIAS1 |
| 215090_x_at | 1.3872732 | LOC100510707///LOC101060303///LOC101060321///LOC101060351  ///LOC101060367///LOC101060376///LOC101060389///LOC101060403  ///LOC101060421///LOC101060440///LOC101060471///LOC101060489  ///LOC101060506///LOC101060522///LOC440434///NPEPPS///TBC1D3  ///TBC1D3F |
| 213115_at | 1.3872328 | ATG4A |
| 222651_s_at | 1.387119 | TRPS1 |
| 202195_s_at | 1.3870641 | TMED5 |
| 234219_at | 1.3870105 | OTTHUMG00000015137///RP11-30P6.6 |
| 205018_s_at | 1.3866955 | MBNL2 |
| 226591_at | 1.3866314 | LOC100506965 |
| 225189_s_at | 1.3865031 | RAPH1 |
| 222956_at | 1.3863702 | FIGN |
| 203843_at | 1.3862355 | RPS6KA3 |
| 217483_at | 1.3862089 | FOLH1 |
| 232751_at | 1.3862089 | RBBP9 |
| 1565949_x_at | 1.3861498 | CHML |
| 224905_at | 1.3860339 | WDR26 |
| 229598_at | 1.385759 | COBLL1 |
| 214358_at | 1.3857567 | ACACA |
| 208177_at | 1.3857435 | SLC34A1 |
| 233955_x_at | 1.3856134 | CXXC5 |
| 204554_at | 1.3853492 | PPP1R3D |
| 222879_s_at | 1.3853483 | POLH |
| 209565_at | 1.3852571 | RNF113A |
| 227056_at | 1.3850479 | KIAA0141 |
| 213652_at | 1.3850392 | PCSK5 |
| 203720_s_at | 1.3846182 | ERCC1 |
| 200761_s_at | 1.3844662 | ARL6IP5 |
| 210305_at | 1.3843466 | PDE4DIP |
| 1563392_at | 1.3843464 |  |
| 229212_at | 1.3843464 |  |
| 228223_at | 1.3843188 | ZSWIM3 |
| 222942_s_at | 1.3842635 | LOC100505519///TIAM2 |
| 1556357_s_at | 1.3838767 | ERICH1 |
| 203413_at | 1.3838226 | NELL2 |
| 227757_at | 1.3834096 | CUL4A |
| 1552734_at | 1.3833508 | RICTOR |
| 202679_at | 1.3832294 | NPC1 |
| 1560503_a_at | 1.383192 | LOC100130275 |
| 241442_at | 1.3827741 |  |
| 1554041_at | 1.3827739 | TMEM239 |
| 204417_at | 1.3827237 | GALC |
| 222853_at | 1.382723 | FLRT3 |
| 228712_at | 1.382723 | WNK1 |
| 211249_at | 1.3825183 | GPR68 |
| 229315_at | 1.3824122 |  |
| 216367_at | 1.3820696 | COL4A3 |
| 209532_at | 1.381358 | PLAA |
| 210862_s_at | 1.3812923 | SARDH |
| 204157_s_at | 1.380919 | SIK3 |
| 214117_s_at | 1.3808692 | BTD |
| 227669_at | 1.3807346 | MPC2 |
| 1563259_at | 1.3807218 |  |
| 238724_at | 1.3807218 |  |
| 201790_s_at | 1.3803636 | DHCR7 |
| 226795_at | 1.3803358 | LRCH1 |
| 212374_at | 1.3802085 | FEM1B |
| 1554717_a_at | 1.3800776 | PDE4D |
| 212511_at | 1.3795913 | PICALM |
| 1560318_at | 1.3794993 | ARHGAP29 |
| 1569482_at | 1.3794993 |  |
| 224013_s_at | 1.378968 | SOX7 |
| 241334_at | 1.3789678 |  |
| 241699_at | 1.3789675 |  |
| 1552902_a_at | 1.378955 | FOXP2 |
| 205510_s_at | 1.3789431 | FLJ10038 |
| 218938_at | 1.3789095 | FBXL15 |
| 1569086_at | 1.3788373 | LOC100287177 |
| 217613_at | 1.3786453 | TMEM144 |
| 220324_at | 1.3786451 | LINC00472 |
| 212288_at | 1.3785526 | FNBP1 |
| 209576_at | 1.3784428 | GNAI1 |
| 227006_at | 1.378348 | PPP1R14A |
| 229790_at | 1.3782091 | TERF2 |
| 222258_s_at | 1.3780738 | SH3BP4 |
| 216733_s_at | 1.3779125 | GATM |
| 1562934_at | 1.3778044 | OTTHUMG00000163260///RP11-756H20.1 |
| 223028_s_at | 1.3777851 | SNX9 |
| 229648_at | 1.3775487 | ARHGAP32 |
| 1561042_at | 1.3775201 | CTA-204B4.6///OTTHUMG00000172354 |
| 1557918_s_at | 1.3774176 | SLC16A1 |
| 216222_s_at | 1.3774172 | MYO10 |
| 201473_at | 1.3774135 | JUNB |
| 204421_s_at | 1.3772643 | FGF2 |
| 226047_at | 1.3772643 | MRVI1 |
| 229935_s_at | 1.3772638 | KMT2A |
| 207111_at | 1.3771993 | EMR1 |
| 222307_at | 1.3771987 | PDCD4-AS1 |
| 222295_x_at | 1.3771276 |  |
| 201137_s_at | 1.3770994 | HLA-DPB1 |
| 202030_at | 1.3767158 | BCKDK |
| 219945_at | 1.3766707 | DDX25 |
| 235453_at | 1.3766702 | TOR1AIP2 |
| 1554878_a_at | 1.3763468 | ABCD3 |
| 1554047_at | 1.3761417 | TXNDC9 |
| 230332_at | 1.3761089 | ZCCHC7 |
| 223734_at | 1.3761086 | MGARP |
| 1557118_a_at | 1.3761084 | INTS6-AS1 |
| 224337_s_at | 1.3761084 | FZD4 |
| 234613_at | 1.3761084 |  |
| 1559517_a_at | 1.3760939 | SPIRE1 |
| 1553300_a_at | 1.3758678 | DGKH |
| 206172_at | 1.3757706 | IL13RA2 |
| 238354_x_at | 1.3753196 |  |
| 205742_at | 1.3753191 | TNNI3 |
| 227435_at | 1.3752522 | KIAA2018 |
| 219950_s_at | 1.3752118 | TIAM2 |
| 240557_at | 1.3750772 | OTTHUMG00000175836///RP11-145F16.2 |
| 201250_s_at | 1.3750759 | SLC2A1 |
| 227015_at | 1.3748319 | ASPHD2 |
| 214968_at | 1.3747846 | DDX51 |
| 215441_at | 1.3747844 |  |
| 203313_s_at | 1.3747264 | TGIF1 |
| 219817_at | 1.3746283 | MAPKAPK5-AS1 |
| 244300_at | 1.374392 |  |
| 216518_at | 1.3743919 |  |
| 210156_s_at | 1.3738344 | PCMT1 |
| 227148_at | 1.3736764 | PLEKHH2 |
| 201691_s_at | 1.3735728 | TPD52 |
| 226606_s_at | 1.3734276 | MTG2 |
| 231233_at | 1.3734094 | PCAT6 |
| 215236_s_at | 1.373251 | PICALM |
| 228201_at | 1.3731171 | ARL13B |
| 200987_x_at | 1.3730935 | PSME3 |
| 214505_s_at | 1.37299 | FHL1 |
| 225123_at | 1.37209 | SESN3 |
| 220294_at | 1.3718932 | KCNV1 |
| 1553132_a_at | 1.3718723 | TC2N |
| 213225_at | 1.3717599 | PPM1B |
| 240983_s_at | 1.3717241 | CARS |
| 209197_at | 1.3716855 | SYT11 |
| 1556769_a_at | 1.3715364 | OTTHUMG00000178410///RP11-874J12.4 |
| 205030_at | 1.3713872 | FABP7 |
| 220565_at | 1.3713864 | CCR10 |
| 1560105_at | 1.3712302 | PTPRB |
| 206825_at | 1.3712295 | OXTR |
| 213822_s_at | 1.3711234 | UBE3B |
| 215523_at | 1.3707637 | ZNF391 |
| 228328_at | 1.3707347 | KLHL28 |
| 1562065_at | 1.3707345 | OTTHUMG00000181950///RP11-794M8.1 |
| 209685_s_at | 1.3706849 | PRKCB |
| 210836_x_at | 1.3706278 | PDE4D |
| 239423_at | 1.3705245 |  |
| 233174_at | 1.3703763 | LOC100287015 |
| 206853_s_at | 1.3703212 | MAP3K7 |
| 212762_s_at | 1.3701699 | TCF7L2 |
| 1554841_at | 1.3696034 | MTHFD2L |
| 202254_at | 1.369603 | SIPA1L1 |
| 1569040_s_at | 1.3695031 | ANKRD36BP2///LOC101060554 |
| 229537_at | 1.3695029 | LMO4 |
| 1561398_at | 1.3694568 | LOC100996590 |
| 1561448_at | 1.3694568 | OTTHUMG00000157317///RP4-630C24.3 |
| 219160_s_at | 1.3694565 | PAPOLG |
| 1553432_s_at | 1.3694563 | LOC101060424///LOC653786///OTOA |
| 1563051_at | 1.3694559 | OSBP |
| 210843_s_at | 1.3694559 | MFAP3L |
| 219513_s_at | 1.3692532 | SH2D3A |
| 206942_s_at | 1.3691146 | PMCH |
| 1569147_at | 1.3689024 | CTC-351M12.1///OTTHUMG00000163000 |
| 219326_s_at | 1.3688997 | B3GNT2 |
| 224874_at | 1.3688468 | POLR1D |
| 203275_at | 1.3681068 | IRF2 |
| 239402_at | 1.3681068 |  |
| 238856_s_at | 1.3680372 | PANK2 |
| 212508_at | 1.3680164 | MOAP1 |
| 232755_at | 1.3679603 | UBE2G2 |
| 208003_s_at | 1.3676728 | NFAT5 |
| 204875_s_at | 1.3676583 | GMDS |
| 213182_x_at | 1.3676565 | CDKN1C |
| 214094_at | 1.3675498 | FUBP1 |
| 49077_at | 1.367548 | PPME1 |
| 230050_at | 1.367515 | NACC2 |
| 231412_at | 1.3674612 | LOC100506342 |
| 239282_at | 1.3674567 | CCDC41 |
| 218191_s_at | 1.3670781 | LMBRD1 |
| 206290_s_at | 1.3670635 | RGS7 |
| 1562527_at | 1.367013 | LOC441666 |
| 204369_at | 1.366975 | PIK3CA |
| 224047_at | 1.3668824 |  |
| 235171_at | 1.366881 | LOC100505501 |
| 223360_at | 1.3668792 | SPATC1L |
| 1554101_a_at | 1.366703 | TMTC4 |
| 1558279_a_at | 1.3665513 | KDSR |
| 1563646_a_at | 1.3664474 | TMEM67 |
| 1558290_a_at | 1.3662993 | MIR1204///PVT1 |
| 1566851_at | 1.3662794 | TRIM42 |
| 223766_at | 1.3662794 | LOC100133130 |
| 227340_s_at | 1.3662794 | RGMB |
| 211478_s_at | 1.3659787 | DPP4 |
| 232229_at | 1.365855 | SETX |
| 236831_at | 1.365669 | CCDC50 |
| 228946_at | 1.365518 | INTU |
| 201539_s_at | 1.3654578 | FHL1 |
| 201105_at | 1.3654356 | LGALS1 |
| 212636_at | 1.3652809 | QKI |
| 240494_at | 1.3652809 |  |
| 242372_s_at | 1.3652809 | MFSD4 |
| 1555950_a_at | 1.3651978 | CD55 |
| 1553640_at | 1.3651762 | XKR6 |
| 1556649_at | 1.3650758 |  |
| 230003_at | 1.3650701 | SLC16A7 |
| 213810_s_at | 1.3650697 | AKIRIN2-AS1///AKIRIN2-AS1 |
| 215150_at | 1.3650697 | YOD1 |
| 242766_at | 1.3650697 | AP001347.6///OTTHUMG00000074260 |
| 223126_s_at | 1.3648865 | C1orf21 |
| 226390_at | 1.3648784 | STARD4 |
| 202766_s_at | 1.3648134 | FBN1 |
| 209911_x_at | 1.3644959 | HIST1H2BD |
| 228693_at | 1.3644418 | CCDC50 |
| 226352_at | 1.3643552 | JMY |
| 242307_at | 1.3642429 | ZNF789 |
| 1556761_at | 1.3642044 |  |
| 210105_s_at | 1.36415 | FYN |
| 229520_s_at | 1.3639945 | GPATCH2L |
| 209227_at | 1.3639337 | TUSC3 |
| 226263_at | 1.3639332 | SNRNP48 |
| 228783_at | 1.3638719 | BVES |
| 228399_at | 1.3636574 | OSR1 |
| 243336_at | 1.3636574 | LOC101059939 |
| 1553461_at | 1.3635349 | FAM9B |
| 1569030_s_at | 1.3633316 | NUB1 |
| 225283_at | 1.3633081 | ARRDC4 |
| 241297_at | 1.3630754 |  |
| 219742_at | 1.3626773 | PRR7 |
| 230579_at | 1.3624017 | LOC728705 |
| 217494_s_at | 1.362388 | PTENP1 |
| 236157_at | 1.362388 | LOC101059993///RBM14///RBM4 |
| 211093_at | 1.3623878 | PDE6C |
| 227557_at | 1.3623878 | SCARF2 |
| 210500_at | 1.3623875 |  |
| 209118_s_at | 1.3623041 | TUBA1A |
| 236772_s_at | 1.3617494 |  |
| 232497_at | 1.3615894 | ZNF3 |
| 201695_s_at | 1.361501 | PNP |
| 1554679_a_at | 1.3614254 | LAPTM4B |
| 236057_at | 1.3613381 | KCTD1 |
| 1558045_a_at | 1.3612806 | LOC100506453///LOC389906///LOC441528///LOC729162 |
| 242781_at | 1.3610457 | FAM199X |
| 231394_at | 1.3610383 |  |
| 226806_s_at | 1.3610313 | NFIA |
| 240955_at | 1.3606834 | PANX3 |
| 238270_x_at | 1.3606832 |  |
| 238476_at | 1.3606832 | CREBRF |
| 230146_s_at | 1.3605887 | NCS1 |
| 231995_at | 1.3605887 | CAAP1 |
| 210718_s_at | 1.3605883 | ARL17A///ARL17B///LOC100294341///LOC100996709 |
| 202022_at | 1.3605878 | ALDOC |
| 211913_s_at | 1.3605077 | MERTK |
| 203577_at | 1.3604844 | GTF2H4 |
| 214368_at | 1.3603653 | RASGRP2 |
| 1563808_at | 1.3603166 | MCF2L |
| 225668_at | 1.3601115 | FAM173B |
| 1552658_a_at | 1.3601112 | NAV3 |
| 227892_at | 1.3601112 | PRKAA2 |
| 1554216_at | 1.360111 | CCDC132 |
| 236311_at | 1.3600407 | LOH12CR2 |
| 221504_s_at | 1.3599683 | ATP6V1H |
| 1569925_at | 1.359953 | DNAH17-AS1 |
| 230019_s_at | 1.3597876 | PTCHD2 |
| 244177_at | 1.3597871 |  |
| 217872_at | 1.359713 | PIH1D1 |
| 236227_at | 1.3595243 | TMEM161B |
| 228421_s_at | 1.35935 | EFEMP1 |
| 205190_at | 1.3593247 | PLS1 |
| 234113_at | 1.359111 |  |
| 208647_at | 1.3589438 | FDFT1 |
| 1562067_at | 1.3588262 |  |
| 1569693_at | 1.3588262 | BTBD8 |
| 214078_at | 1.3588262 |  |
| 225842_at | 1.3588262 | PHLDA1 |
| 240389_at | 1.3588262 | TRPM6 |
| 244206_at | 1.3588262 | ZFAND4 |
| 232216_at | 1.3585962 | YME1L1 |
| 206189_at | 1.3584341 | UNC5C |
| 244582_at | 1.3584341 | AC064852.4///OTTHUMG00000153291 |
| 228496_s_at | 1.3582675 | CRIM1 |
| 243413_at | 1.3582648 | TTC30B |
| 231204_at | 1.3582274 | C4orf21 |
| 223089_at | 1.3580637 | VEZT |
| 218325_s_at | 1.3577307 | DIDO1 |
| 239988_at | 1.3577307 |  |
| 241620_at | 1.3577307 | SMCHD1 |
| 202301_s_at | 1.3576939 | RSRC2 |
| 229618_at | 1.3574189 | SNX16 |
| 1556362_at | 1.3574184 | OTTHUMG00000168466///OTTHUMG00000174864///RP4-809F18.1 |
| 1568876_a_at | 1.3574184 |  |
| 224798_s_at | 1.3571515 | FAM219B |
| 220200_s_at | 1.3571484 | SETD8 |
| 226298_at | 1.3571444 | RUNDC1 |
| 223614_at | 1.3571385 | MMP16 |
| 218498_s_at | 1.3570874 | ERO1L |
| 224631_at | 1.3569833 | ZFP91 |
| 212668_at | 1.3566784 | SMURF1 |
| 229016_s_at | 1.356516 | TRERF1 |
| 229549_at | 1.356352 | CALU |
| 224970_at | 1.3563076 | NFIA |
| 228037_at | 1.3562237 | CTD-2267D19.2///OTTHUMG00000179631 |
| 203367_at | 1.3561529 | DUSP14 |
| 223874_at | 1.3561525 | ACTR3C |
| 1553710_at | 1.356036 | FAM218A |
| 1556392_a_at | 1.3560357 | CTD-2373J6.1///OTTHUMG00000174459 |
| 1562341_at | 1.3560357 | OTTHUMG00000009930///RP11-475O6.1 |
| 243570_at | 1.3560357 | SPCS2 |
| 235439_at | 1.3560352 | RBMS2 |
| 223600_s_at | 1.3559974 | KIAA1683 |
| 1552717_s_at | 1.355997 | CEP170///CEP170P1 |
| 223527_s_at | 1.3559557 | CDADC1 |
| 214500_at | 1.3556564 | H2AFY |
| 222045_s_at | 1.3555264 | PCIF1 |
| 235948_at | 1.3552281 | RIMKLA |
| 236182_at | 1.3552281 | FAM185A |
| 1569253_at | 1.3549916 | INTS4 |
| 210607_at | 1.3549914 | FLT3LG |
| 232477_at | 1.3549914 |  |
| 1559535_s_at | 1.3549912 | CTC-241N9.1///OTTHUMG00000163262 |
| 235362_at | 1.3549912 | LOC729970 |
| 1553736_at | 1.354991 | ZFC3H1 |
| 237326_at | 1.3549907 | LINC00310 |
| 230564_at | 1.3549522 | SIPA1L3 |
| 202205_at | 1.3547391 | VASP |
| 222408_s_at | 1.3544606 | YPEL5 |
| 240317_at | 1.3544153 | PCDHB4 |
| 1555220_a_at | 1.3544145 | AKR1E2 |
| 214079_at | 1.3541877 | DHRS2 |
| 202912_at | 1.3541875 | ADM |
| 206757_at | 1.3540962 | PDE5A |
| 216979_at | 1.3540958 | NR4A3 |
| 223639_s_at | 1.3540944 | ZNRD1 |
| 226773_at | 1.3540102 | PPM1K |
| 218368_s_at | 1.3540049 | TNFRSF12A |
| 222493_s_at | 1.3538353 | ZFAND3 |
| 1560250_s_at | 1.3537292 | LOC284242 |
| 229461_x_at | 1.3537292 | NEGR1 |
| 218696_at | 1.3537257 | EIF2AK3 |
| 207254_at | 1.3537114 | SLC15A1 |
| 211828_s_at | 1.3536756 | TNIK |
| 238921_at | 1.3536756 | LOC644794 |
| 236480_at | 1.3536754 | MIR210HG |
| 231746_at | 1.3536747 | MIXL1 |
| 1560625_s_at | 1.3535368 | AC021016.6///OTTHUMG00000155067 |
| 213362_at | 1.3532711 | PTPRD |
| 206743_s_at | 1.3532703 | ASGR1 |
| 203099_s_at | 1.3531097 | CDYL |
| 243559_at | 1.3529116 |  |
| 209344_at | 1.3527024 | TPM4 |
| 206490_at | 1.3527014 | DLGAP1 |
| 207420_at | 1.3526436 | COLEC10 |
| 221348_at | 1.3526434 | NPPC |
| 207071_s_at | 1.3523304 | ACO1 |
| 230423_at | 1.3522428 | LOC100996301 |
| 224572_s_at | 1.3520104 | IRF2BP2 |
| 228981_at | 1.3518862 | TMEM169 |
| 236119_s_at | 1.3518835 | SPRR2G |
| 214130_s_at | 1.3516213 | LOC100996724///LOC100996761///LOC101060291///LOC101060344  ///LOC101060353///LOC101060463///LOC101060582///LOC728802///  PDE4DIP |
| 202164_s_at | 1.3516065 | CNOT8 |
| 201370_s_at | 1.3515645 | CUL3 |
| 213134_x_at | 1.3515351 | BTG3 |
| 230859_at | 1.3515136 |  |
| 206818_s_at | 1.3512653 | CNNM2 |
| 215195_at | 1.3509924 | PRKCA |
| 206113_s_at | 1.3509746 | RAB5A |
| 215193_x_at | 1.3509736 | HLA-DRB1///HLA-DRB3///HLA-DRB4///LOC100507709///  LOC100507714 |
| 223260_s_at | 1.3508807 | POLK |
| 1569748_at | 1.3508366 | PRKCZ |
| 217624_at | 1.3508366 | PDAP1 |
| 231385_at | 1.3508366 | DPPA3///DPPA3P2///LOC101060236 |
| 233468_at | 1.3508366 |  |
| 240938_at | 1.3508366 | OTTHUMG00000176267///RP11-18F14.1 |
| 242116_x_at | 1.3508366 |  |
| 210198_s_at | 1.3508362 | PLP1 |
| 203827_at | 1.350674 | WIPI1 |
| 226271_at | 1.3505901 | GDAP1 |
| 243727_at | 1.3504759 | CPNE8 |
| 202537_s_at | 1.3504491 | CHMP2B |
| 230788_at | 1.3503009 | GCNT2 |
| 222776_at | 1.3501041 | MAGOHB |
| 1559108_at | 1.3500575 | LOC100996816///VPS53 |
| 204298_s_at | 1.3500572 | LOX |
| 210670_at | 1.3500572 | PPY |
| 237860_at | 1.3500572 | RBMS3 |
| 233288_at | 1.3499298 | ATR |
| 1555573_at | 1.3499292 | TTC40 |
| 237810_at | 1.3498654 | CLDN6 |
| 230051_at | 1.3494653 | PROSER2 |
| 202619_s_at | 1.3493444 | PLOD2 |
| 235463_s_at | 1.3490893 | CERS6 |
| 238907_at | 1.3488468 | ZNF780A |
| 1558948_a_at | 1.3485906 |  |
| 225378_at | 1.348417 | VPS37A |
| 1554309_at | 1.3479842 | EIF4G3 |
| 238706_at | 1.347984 | PAPD4 |
| 1570480_s_at | 1.3479314 | ART1 |
| 235899_at | 1.3479314 | CA13///LOC100507258 |
| 1553286_at | 1.3476654 | ZNF555 |
| 243032_at | 1.3476493 |  |
| 1569374_at | 1.347649 | C3orf62 |
| 202133_at | 1.3476018 | WWTR1 |
| 1569699_at | 1.3473781 | AK7 |
| 204850_s_at | 1.3473779 | DCX |
| 1555174_at | 1.3473476 |  |
| 1557179_s_at | 1.3473476 | CARS2 |
| 215767_at | 1.3473471 | ZNF804A |
| 237548_at | 1.3473471 |  |
| 204637_at | 1.3472294 | CGA |
| 222814_s_at | 1.347228 | ZNHIT2 |
| 209146_at | 1.3472 | MSMO1 |
| 214888_at | 1.3471279 | CAPN2 |
| 225539_at | 1.3470486 | ZBTB21 |
| 214378_at | 1.3469845 | TFPI |
| 224127_at | 1.3467426 |  |
| 212619_at | 1.3467424 | TMEM194A |
| 235850_at | 1.346575 | FAM162A |
| 203928_x_at | 1.3463609 | MAPT |
| 211673_s_at | 1.3463378 | MOCS1 |
| 204113_at | 1.3456763 | CELF1 |
| 1556236_at | 1.3455607 |  |
| 211561_x_at | 1.345509 | MAPK14 |
| 1562894_at | 1.3453821 |  |
| 213756_s_at | 1.3453819 | HSF1 |
| 220545_s_at | 1.3453816 | TSKS |
| 207096_at | 1.3451204 | SAA2-SAA4///SAA4 |
| 233233_at | 1.3450502 | RASSF3 |
| 219768_at | 1.3449957 | VTCN1 |
| 243982_at | 1.3448368 |  |
| 1557174_a_at | 1.3448323 | IRAK1BP1 |
| 202499_s_at | 1.3448279 | SLC2A3 |
| 222409_at | 1.3447034 | CORO1C |
| 236081_at | 1.3446751 | SNCA |
| 219276_x_at | 1.344627 | CAAP1 |
| 223641_at | 1.344448 |  |
| 215053_at | 1.3442742 | SRCAP |
| 224245_at | 1.3442739 | INGX |
| 218562_s_at | 1.3440645 | TMEM57 |
| 242243_at | 1.3438735 | TMF1 |
| 214151_s_at | 1.3438699 | CCPG1///DYX1C1-CCPG1 |
| 232579_at | 1.3437673 | LOC100134229 |
| 1553133_at | 1.3434684 | C9orf72 |
| 217499_x_at | 1.3430796 | OR7E37P |
| 215997_s_at | 1.3430511 | CUL4B |
| 64432_at | 1.3429738 | MAPKAPK5-AS1 |
| 222067_x_at | 1.3429393 | HIST1H2BD |
| 227909_at | 1.3427546 | LINC00086///LINC00087 |
| 234040_at | 1.3427546 | HELLS |
| 213338_at | 1.3426126 | TMEM158 |
| 211000_s_at | 1.3424742 | IL6ST |
| 1565582_at | 1.3424147 |  |
| 241809_at | 1.3423277 | FAM212B |
| 220841_s_at | 1.3423042 | AHI1 |
| 213355_at | 1.3422558 | ST3GAL6 |
| 223312_at | 1.3421601 | PRADC1 |
| 221875_x_at | 1.3419862 | HLA-F |
| 1552978_a_at | 1.3418828 | SCAMP1 |
| 1563147_at | 1.3417112 |  |
| 207613_s_at | 1.3417112 | CAMK2A |
| 224805_s_at | 1.3416412 | FAM219B |
| 243917_at | 1.3414257 | CLIC5 |
| 206786_at | 1.3414252 | HTN3 |
| 1570432_at | 1.3413372 |  |
| 238785_at | 1.341337 |  |
| 220506_at | 1.3412572 | GUCY1B2 |
| 231833_at | 1.3412567 | RBM33 |
| 240419_at | 1.3409947 | SLC6A15 |
| 230774_at | 1.3407749 | PTGR2 |
| 236478_at | 1.3407333 | IFNAR1 |
| 202719_s_at | 1.3406845 | TES |
| 210583_at | 1.3403106 | POLDIP3 |
| 230935_at | 1.3403106 | AC009336.24///OTTHUMG00000154176 |
| 227368_at | 1.3402721 |  |
| 227542_at | 1.3400218 | SOCS6 |
| 224480_s_at | 1.3400178 | AGPAT9 |
| 225750_at | 1.3399705 | ERO1L |
| 206024_at | 1.3398845 | HPD |
| 1555202_a_at | 1.3398836 | RPRD1A |
| 205472_s_at | 1.3398836 | DACH1 |
| 232089_at | 1.3397402 | LINC00921///ZNF263 |
| 226454_at | 1.3396958 | 9-Mar |
| 229412_at | 1.3396782 | TAF8 |
| 213899_at | 1.3396214 | METAP2 |
| 211998_at | 1.339586 | H3F3A///H3F3B///MIR4738 |
| 230951_at | 1.3395338 | EPB41L5 |
| 208067_x_at | 1.3395312 | UTY |
| 236821_at | 1.3395312 |  |
| 242005_at | 1.3395312 | LOC100506377 |
| 1559705_s_at | 1.3393399 | PHKA2 |
| 231389_at | 1.3391633 | PIH1D3 |
| 1554053_at | 1.3389858 | SPTLC1 |
| 242317_at | 1.3388681 | HIGD1A |
| 203078_at | 1.3388584 | CUL2 |
| 229010_at | 1.3387327 | CBL |
| 1569133_x_at | 1.338615 | ARSK |
| 212874_at | 1.3384858 |  |
| 1569146_s_at | 1.3384522 | KIAA0408///SOGA3 |
| 226812_at | 1.3384522 |  |
| 231991_at | 1.3384522 | CCM2L |
| 201442_s_at | 1.3384517 | ATP6AP2 |
| 237515_at | 1.3383894 | TMEM56 |
| 1560305_x_at | 1.3383243 | FKBP4 |
| 211084_x_at | 1.337791 | PRKD3 |
| 230821_at | 1.3377746 | ZNF148 |
| 1553944_at | 1.3376287 | CTAGE5///MIA2 |
| 212746_s_at | 1.3375262 | CEP170 |
| 1558967_s_at | 1.3373387 | HCG18 |
| 237156_at | 1.3373387 |  |
| 221760_at | 1.3371663 | MAN1A1 |
| 243098_at | 1.3371663 | AP000696.2///OTTHUMG00000086644 |
| 237043_at | 1.3371305 |  |
| 1555691_a_at | 1.3369874 | KLRC4-KLRK1///KLRK1 |
| 221808_at | 1.3369322 | RAB9A |
| 212095_s_at | 1.3368632 | MTUS1 |
| 209846_s_at | 1.3365451 | BTN3A2 |
| 201242_s_at | 1.3363985 | ATP1B1 |
| 200737_at | 1.3363667 | PGK1 |
| 235162_at | 1.3362032 | MDM4 |
| 237096_at | 1.336203 |  |
| 204491_at | 1.335473 | PDE4D |
| 225028_at | 1.3353701 | LOC550643 |
| 225717_at | 1.3353472 | KIAA1715 |
| 239578_at | 1.335314 |  |
| 209961_s_at | 1.335259 | HGF |
| 216268_s_at | 1.3351538 | JAG1 |
| 220150_s_at | 1.3351058 | FAM184A |
| 238183_at | 1.3350607 |  |
| 235645_at | 1.3350127 | ESCO1 |
| 219188_s_at | 1.3349761 | MACROD1 |
| 236453_at | 1.3349751 |  |
| 210145_at | 1.3348587 | PLA2G4A |
| 221910_at | 1.3346884 | ETV1 |
| 222846_at | 1.3346786 | RAB8B |
| 231508_s_at | 1.334627 |  |
| 239703_at | 1.3346267 | CTD-2537I9.5///OTTHUMG00000180856 |
| 236200_at | 1.3345569 | FAF1 |
| 230104_s_at | 1.3345401 | TPPP |
| 206075_s_at | 1.3344498 | CSNK2A1 |
| 1555610_at | 1.3344474 | AGK |
| 232090_at | 1.3344405 | DNM3OS |
| 203574_at | 1.3344171 | NFIL3 |
| 235968_at | 1.3342936 | AGAP1 |
| 224507_s_at | 1.3342183 | MGC12916 |
| 203227_s_at | 1.3340154 | TSPAN31 |
| 208936_x_at | 1.3340154 | LGALS8 |
| 222909_s_at | 1.3340154 | BAG4 |
| 241490_s_at | 1.3340154 | PGBD2 |
| 1566538_at | 1.3340148 | MAGI1 |
| 219733_s_at | 1.3338381 | SLC27A5 |
| 216976_s_at | 1.3337425 | RYK |
| 237456_at | 1.3336649 |  |
| 231978_at | 1.3336285 | TPCN2 |
| 1552928_s_at | 1.3336283 | TAB3 |
| 231380_at | 1.3336283 | C8orf34 |
| 232893_at | 1.3336283 | LMBRD2 |
| 243550_at | 1.3336283 | ZDHHC21 |
| 200706_s_at | 1.3335904 | LITAF |
| 1558666_at | 1.3334507 | PHEX-AS1 |
| 207893_at | 1.3334507 | SRY |
| 232794_at | 1.3334504 | LOC153682 |
| 205461_at | 1.3334502 | RAB35 |
| 229976_at | 1.3333948 | MORN5 |
| 239177_at | 1.3333948 | IRGQ |
| 209590_at | 1.3329691 | BMP7 |
| 1570454_at | 1.3329668 | EIF4EBP2 |
| 1569132_s_at | 1.33294 | ARSK |
| 1558855_at | 1.3329241 | FARP2 |
| 236795_at | 1.3329241 |  |
| 244610_x_at | 1.3329239 |  |
| 1555560_at | 1.3329237 | UGGT2 |
| 208576_s_at | 1.3329237 | HIST1H3A///HIST1H3B///HIST1H3C///HIST1H3D///HIST1H3E///  HIST1H3F///HIST1H3G///HIST1H3H///HIST1H3I///HIST1H3J |
| 218692_at | 1.3329175 | SYBU |
| 235132_at | 1.3328391 | LOC254128 |
| 214633_at | 1.3327646 | SOX3 |
| 214509_at | 1.3327644 | HIST1H3A///HIST1H3B///HIST1H3C///HIST1H3D///HIST1H3E///  HIST1H3F///HIST1H3G///HIST1H3H///HIST1H3I///HIST1H3J |
| 1553811_at | 1.332747 | FAM222A-AS1 |
| 1570473_at | 1.3327464 | ANKRD30BP3 |
| 242618_at | 1.3326237 | HCG18 |
| 204173_at | 1.3325541 | MYL6B |
| 228889_at | 1.332533 | ARHGAP5-AS1 |
| 213526_s_at | 1.3325013 | LIN37 |
| 1558885_at | 1.3323727 |  |
| 229819_at | 1.3323722 | A1BG |
| 233705_at | 1.332311 | PACSIN2 |
| 207628_s_at | 1.3322247 | WBSCR22 |
| 220950_s_at | 1.3320398 | KANSL3 |
| 222636_at | 1.331809 | MED28 |
| 1555990_at | 1.3315848 | C22orf42 |
| 1562495_at | 1.3315215 | ANKFY1 |
| 228241_at | 1.3315213 | AGR3 |
| 239739_at | 1.3315213 | SNX24 |
| 235298_at | 1.3312132 | WDR27 |
| 220658_s_at | 1.3312128 | ARNTL2 |
| 244556_at | 1.3310839 | LCP2 |
| 225325_at | 1.3309351 | MFSD6 |
| 1560706_at | 1.330935 | NEDD9 |
| 1556259_at | 1.3309348 |  |
| 201207_at | 1.330908 | TNFAIP1 |
| 236600_at | 1.3307918 | SPG20 |
| 209806_at | 1.3307662 | HIST1H2BK |
| 1553020_at | 1.330748 | SMCR5 |
| 201849_at | 1.3306229 | BNIP3 |
| 219842_at | 1.3305837 | ARL15 |
| 1566908_at | 1.3305331 | C9orf173 |
| 206767_at | 1.330494 | RBMS3 |
| 1553218_a_at | 1.3304844 | ZNF512 |
| 222971_at | 1.3304285 | NDUFA2 |
| 204567_s_at | 1.3302654 | ABCG1 |
| 203642_s_at | 1.3302324 | COBLL1 |
| 202457_s_at | 1.330155 | PPP3CA |
| 205618_at | 1.3299 | PRRG1 |
| 238099_at | 1.3297726 |  |
| 208173_at | 1.3295133 | IFNB1 |
| 236915_at | 1.3295133 | C4orf47 |
| 1553212_at | 1.3293165 | KRT78 |
| 203483_at | 1.3293165 | SEMA4G |
| 1558447_at | 1.3292863 |  |
| 236916_at | 1.3292229 |  |
| 208621_s_at | 1.3291758 | EZR |
| 240102_at | 1.3290178 |  |
| 1569021_at | 1.3290173 | PIK3C2A |
| 1569372_at | 1.3290173 | LOC100507194 |
| 234808_x_at | 1.3290173 |  |
| 218645_at | 1.3288509 | ZNF277 |
| 210033_s_at | 1.3287438 | SPAG6 |
| 220092_s_at | 1.3287435 | ANTXR1 |
| 231420_at | 1.3286778 | GGN |
| 219662_at | 1.3282694 | C2orf49 |
| 1559980_at | 1.3282477 | ZNF7 |
| 1558449_at | 1.3282474 | OTTHUMG00000177541///RP11-421E14.2 |
| 232734_at | 1.3282474 | TTC23 |
| 1554923_at | 1.3282224 | ANKS6 |
| 219886_at | 1.328071 | CEP97 |
| 201505_at | 1.3280447 | LAMB1 |
| 243405_at | 1.3279516 |  |
| 221756_at | 1.3278186 | PIK3IP1 |
| 1554451_s_at | 1.3278116 | DNAJC14 |
| 235624_at | 1.3276075 | HDLBP |
| 223949_at | 1.3275185 | TMPRSS3 |
| 231059_x_at | 1.3275075 | SCAND1 |
| 218166_s_at | 1.3274391 | RSF1 |
| 202932_at | 1.3274215 | YES1 |
| 239046_at | 1.3273882 |  |
| 226583_at | 1.3272613 | C12orf76 |
| 206241_at | 1.327056 | KPNA5 |
| 235603_at | 1.3270082 | HNRNPU |
| 200704_at | 1.3269916 | LITAF |
| 221673_s_at | 1.3269618 | CSNK1G1 |
| 225898_at | 1.3269144 | WDR54 |
| 202600_s_at | 1.3265128 | NRIP1 |
| 205701_at | 1.3264755 | IPO8 |
| 1553713_a_at | 1.3264624 | RHEBL1 |
| 238971_at | 1.3263357 |  |
| 223284_at | 1.3263006 | NAT14 |
| 200841_s_at | 1.3262848 | EPRS |
| 203263_s_at | 1.326198 | ARHGEF9 |
| 222736_s_at | 1.326134 | TMEM38B |
| 1564510_at | 1.325923 |  |
| 228580_at | 1.3259227 | HTRA3 |
| 238248_at | 1.3259227 |  |
| 206433_s_at | 1.3259225 | SPOCK3 |
| 242730_at | 1.3259225 | MYRIP |
| 233506_at | 1.3257452 | OTTHUMG00000176282///RP11-469M7.1 |
| 217217_at | 1.3256791 | IGHA1///IGHA2///IGHG1///IGHG4///IGHM///IGHV4-31 |
| 202578_s_at | 1.3256291 | DDX19A |
| 222126_at | 1.3254907 | AGFG2 |
| 243460_at | 1.3254747 |  |
| 1555340_x_at | 1.3254745 | RAP1A |
| 238426_at | 1.3254745 | TMEM130 |
| 238483_at | 1.3254745 | SSBP2 |
| 242650_at | 1.3254745 |  |
| 1554742_at | 1.3254743 | PMS1 |
| 233902_at | 1.3254743 | GUCA1C |
| 1555809_at | 1.325474 | CRISPLD2 |
| 231932_at | 1.325474 |  |
| 226873_at | 1.3254172 | FAM63B |
| 202422_s_at | 1.3253769 | ACSL4 |
| 239202_at | 1.3253163 | RAB3B |
| 242587_at | 1.3247199 | SLC9A9 |
| 237375_at | 1.3247194 | AC012499.1///OTTHUMG00000154189 |
| 238081_at | 1.3247194 | WDFY3-AS2 |
| 223122_s_at | 1.3247192 | SFRP2 |
| 227252_at | 1.324628 | LRP10 |
| 211518_s_at | 1.3245833 | BMP4 |
| 213429_at | 1.3245828 | BICC1 |
| 216698_x_at | 1.3244537 | OR7E12P |
| 209201_x_at | 1.3243705 | CXCR4 |
| 207039_at | 1.3243556 | CDKN2A |
| 1552649_a_at | 1.324234 | RAD51L3-RFFL///RFFL |
| 214697_s_at | 1.3241683 | PTBP3 |
| 1561609_at | 1.3240913 |  |
| 207049_at | 1.3240913 | SCN8A |
| 230515_at | 1.32403 |  |
| 215627_at | 1.324018 | AC007405.4///OTTHUMG00000154055 |
| 221957_at | 1.3239973 | PDK3 |
| 241352_at | 1.323847 |  |
| 228569_at | 1.3238357 | PAPOLA |
| 1566152_a_at | 1.3236694 | AP000662.4///OTTHUMG00000167137 |
| 228548_at | 1.3236694 | RAP1A |
| 242871_at | 1.3236694 | PAQR5 |
| 229559_at | 1.3235937 | PPM1N |
| 225426_at | 1.323435 | PPP6C |
| 230123_at | 1.3233379 | NECAP2 |
| 238482_at | 1.3231682 | KLF7 |
| 206669_at | 1.322891 | GAD1 |
| 239203_at | 1.3228184 | LSMEM1 |
| 1561917_at | 1.322707 |  |
| 215064_at | 1.3227031 | SC5D |
| 1554310_a_at | 1.322703 | EIF4G3 |
| 1567862_at | 1.322703 | DNAH14 |
| 1555600_s_at | 1.3222737 | APOL4 |
| 243796_at | 1.3222733 |  |
| 224642_at | 1.3221859 | FYTTD1 |
| 244694_at | 1.3220937 | IGLON5 |
| 214759_at | 1.3220552 | WTAP |
| 225131_at | 1.3219084 | ZRANB1 |
| 217520_x_at | 1.321828 | LOC283683///LOC646278 |
| 225332_at | 1.3217441 | OIP5-AS1 |
| 202684_s_at | 1.3217193 | RNMT |
| 1564152_at | 1.3216655 | FLJ35816 |
| 240680_at | 1.3216655 |  |
| 205820_s_at | 1.3216393 | APOC3 |
| 217002_s_at | 1.3215513 | HTR3A |
| 204128_s_at | 1.3215414 | RFC3 |
| 204560_at | 1.3214288 | FKBP5 |
| 225352_at | 1.3213406 | SEC62 |
| 230091_at | 1.3212322 |  |
| 238682_at | 1.3212235 | CCDC96 |
| 227004_at | 1.321199 |  |
| 208678_at | 1.3211466 | ATP6V1E1 |
| 226268_at | 1.3211353 | RAB21 |
| 201915_at | 1.3211061 | SEC63 |
| 209475_at | 1.321089 | USP15 |
| 213694_at | 1.3210087 | RSBN1 |
| 204620_s_at | 1.3209711 | VCAN |
| 214503_x_at | 1.3209711 | GPR135 |
| 225818_s_at | 1.3209711 | TBRG1 |
| 238323_at | 1.3209711 | TEAD2 |
| 243943_x_at | 1.3209711 | C6orf52 |
| 216060_s_at | 1.3206612 | DAAM1 |
| 204969_s_at | 1.3206551 | RDX |
| 215682_at | 1.3204416 | LOC440792 |
| 1569974_x_at | 1.3204032 | SEPT7P2 |
| 204932_at | 1.3204027 | TNFRSF11B |
| 210587_at | 1.3204027 | INHBE |
| 210739_x_at | 1.3204027 | SLC4A4 |
| 211958_at | 1.3204027 | IGFBP5 |
| 243793_at | 1.3204027 | AHDC1 |
| 202314_at | 1.3203347 | CYP51A1///LRRD1 |
| 242196_at | 1.320203 | ARHGAP32 |
| 230652_at | 1.3202003 | ARAF |
| 228205_at | 1.3201252 | TKT |
| 203817_at | 1.3200598 | GUCY1B3 |
| 225333_at | 1.320059 | ZNF496 |
| 244683_at | 1.320059 |  |
| 232690_at | 1.3199838 | CTC-205M6.1///OTTHUMG00000162253 |
| 214764_at | 1.3199333 | RRP15 |
| 233069_at | 1.3196428 | PPP4R1L |
| 230591_at | 1.3195581 | LOC729887 |
| 238587_at | 1.3195573 | UBASH3B |
| 217154_s_at | 1.3192912 | EDN3 |
| 234967_at | 1.3192912 | IL6ST |
| 203725_at | 1.3191273 | GADD45A |
| 215489_x_at | 1.3187785 | HOMER3 |
| 230766_at | 1.3186303 | GART |
| 213065_at | 1.3185798 | ZFC3H1 |
| 203045_at | 1.3185658 | NINJ1 |
| 1561479_at | 1.3184608 | OTTHUMG00000176289///RP11-632P5.1 |
| 207957_s_at | 1.3184606 | PRKCB |
| 218176_at | 1.3183863 | MAGEF1 |
| 221260_s_at | 1.3182992 | CSRNP2 |
| 235130_at | 1.3182644 | PANK2 |
| 227912_s_at | 1.3181006 | EXOSC3 |
| 214437_s_at | 1.3180727 | SHMT2 |
| 209591_s_at | 1.3180604 | BMP7 |
| 1555910_at | 1.3179193 | PTCD2 |
| 206009_at | 1.31782 | ITGA9 |
| 1559648_at | 1.317592 | LINC00892 |
| 1562888_at | 1.3175918 | GLB1L3 |
| 223438_s_at | 1.3175918 | PPARA |
| 1556331_a_at | 1.3175478 |  |
| 222124_at | 1.3175478 | HIF3A |
| 210634_at | 1.3175473 | KLHL20 |
| 221648_s_at | 1.3175473 | AGMAT |
| 240412_s_at | 1.3175473 | MROH2B |
| 1555171_at | 1.3175471 | ST3GAL3 |
| 200670_at | 1.3175447 | XBP1 |
| 1552261_at | 1.3174968 | WFDC2 |
| 237384_x_at | 1.3174968 | CTB-85P21.2///OTTHUMG00000175439 |
| 1556472_s_at | 1.3174964 | SCML4 |
| 210068_s_at | 1.3174964 | AQP4 |
| 219354_at | 1.3170375 | KLHL26 |
| 201611_s_at | 1.3170371 | ICMT |
| 215891_s_at | 1.3169761 | GM2A |
| 1553449_at | 1.31652 | LINC00304 |
| 233903_s_at | 1.3163412 | ARHGEF26 |
| 1556801_at | 1.3163016 | LOC400794 |
| 218206_x_at | 1.3161188 | SCAND1 |
| 219596_at | 1.3161145 | THAP10 |
| 217965_s_at | 1.315938 | SAP30BP |
| 241771_at | 1.3159267 | RIMBP2 |
| 204672_s_at | 1.3158932 | ANKRD6 |
| 225317_at | 1.3158736 | ACBD6 |
| 222717_at | 1.3154896 | SDPR |
| 233731_at | 1.3154896 |  |
| 1562722_at | 1.3154894 | PRR20A///PRR20B///PRR20C///PRR20D///PRR20E |
| 227382_at | 1.3154235 | CYB5B |
| 204647_at | 1.3154092 | HOMER3 |
| 1566277_at | 1.3153635 | OR5E1P |
| 1565906_at | 1.3153633 | NADSYN1 |
| 1554339_a_at | 1.315363 | COG3 |
| 228828_at | 1.315363 | BZRAP1-AS1 |
| 237147_at | 1.315363 |  |
| 1567860_at | 1.3153627 |  |
| 227649_s_at | 1.3153627 | SRGAP2///SRGAP2D |
| 209228_x_at | 1.3152405 | TUSC3 |
| 234350_at | 1.315131 | IGLC1 |
| 1554509_a_at | 1.315104 | FAM188A |
| 223454_at | 1.3150309 | CXCL16 |
| 205196_s_at | 1.3149049 | AP1S1 |
| 202407_s_at | 1.3147433 | PRPF31 |
| 224115_at | 1.3146976 |  |
| 226713_at | 1.3146825 | CCDC50 |
| 234306_s_at | 1.3144984 | SLAMF7 |
| 222270_at | 1.3144683 | SMEK2 |
| 204913_s_at | 1.3144183 | SOX11 |
| 220382_s_at | 1.3144183 | ARHGAP28 |
| 227850_x_at | 1.3144181 | CDC42EP5 |
| 635_s_at | 1.3144035 | PPP2R5B |
| 223125_s_at | 1.3142667 | C1orf21 |
| 224064_s_at | 1.3142493 | DHDDS |
| 1553672_at | 1.314238 | ENAH |
| 38241_at | 1.3138919 | BTN3A3 |
| 243862_at | 1.3138788 | RASEF |
| 1554192_s_at | 1.3138666 | TRMT44 |
| 1553691_at | 1.3138467 | B3GALNT2 |
| 224359_s_at | 1.3138467 | HOOK3 |
| 1557192_at | 1.313815 | COX10-AS1///LOC100506974 |
| 206648_at | 1.3138137 | ZNF571 |
| 228730_s_at | 1.3138132 | SCRN2 |
| 228075_x_at | 1.3137598 | TFB1M |
| 236850_at | 1.3136879 | CAPRIN1 |
| 1555647_a_at | 1.3136878 | FOXP2 |
| 1559970_at | 1.3136878 | LOC100506731 |
| 1565778_at | 1.3136878 | ABCA8 |
| 235561_at | 1.3136878 | TXNL1 |
| 237776_at | 1.3136878 | ZADH2 |
| 207443_at | 1.3136876 | NR2E1 |
| 1560595_at | 1.3136873 | LOC100128993 |
| 232591_s_at | 1.3135848 | TMEM30A |
| 222777_s_at | 1.3135132 | WHSC1 |
| 240957_at | 1.3135123 |  |
| 242003_at | 1.3135123 | ERICH1 |
| 225108_at | 1.313508 | AGPS |
| 240834_at | 1.3134954 | FAM105B |
| 212803_at | 1.3132949 | NAB2 |
| 208721_s_at | 1.313241 | ANAPC5 |
| 1563687_a_at | 1.3131851 | FRYL |
| 1553327_a_at | 1.3131844 | BEND7 |
| 225527_at | 1.3130201 | CEBPG |
| 219207_at | 1.3129619 | EDC3 |
| 1554106_at | 1.3129138 | NBEAL1 |
| 224829_at | 1.3128786 | CPEB4 |
| 217614_at | 1.3128579 |  |
| 228440_at | 1.3128579 | OTTHUMG00000161674///OTTHUMG00000161676///RP11-164P12.4  ///RP11-164P12.5 |
| 221238_at | 1.3127172 | HMGN5 |
| 1560399_a_at | 1.3125081 | OTUD7A |
| 1565939_at | 1.3125081 | C5orf22 |
| 201835_s_at | 1.3125081 | PRKAB1 |
| 202565_s_at | 1.3125081 | SVIL |
| 1554453_at | 1.3125042 | HNRNPLL |
| 227044_at | 1.3122071 | CTA-29F11.1///OTTHUMG00000172744 |
| 236213_at | 1.3118784 | AC079305.10///OTTHUMG00000154178 |
| 210595_at | 1.3117996 | ZNF235 |
| 201668_x_at | 1.3117973 | MARCKS |
| 216137_s_at | 1.3117969 | MAPK8IP3 |
| 221955_at | 1.3117969 |  |
| 1561219_x_at | 1.3115342 |  |
| 232155_at | 1.3115342 | RNF213 |
| 210871_x_at | 1.3114154 | SSX2IP |
| 218861_at | 1.3113647 | RNF25 |
| 235527_at | 1.3113148 | DLGAP1 |
| 210984_x_at | 1.3112992 | EGFR |
| 223664_x_at | 1.3112403 | BCL2L13 |
| 235011_at | 1.3112065 | MAP3K2 |
| 232369_at | 1.3111675 |  |
| 240674_at | 1.3111675 | JARID2 |
| 1561514_at | 1.311167 | LOC400655 |
| 1554513_s_at | 1.3111666 | CEP89 |
| 1553697_at | 1.3111571 | CCSAP |
| 236187_s_at | 1.3111571 |  |
| 213836_s_at | 1.3110124 | WIPI1 |
| 208729_x_at | 1.3108433 | HLA-B |
| 1560255_at | 1.3107619 | CELF2-AS1 |
| 223772_s_at | 1.3107619 | TMEM87A |
| 229060_at | 1.3107619 | YPEL2 |
| 242750_at | 1.3107619 | MMAA |
| 1554364_at | 1.3107617 | PPP2R5C |
| 203914_x_at | 1.3107129 | HPGD |
| 211490_at | 1.3107127 | ADRA1A |
| 220030_at | 1.310618 | STYK1 |
| 222800_at | 1.3104652 | TRNAU1AP |
| 218816_at | 1.3102958 | LRRC1 |
| 201787_at | 1.310159 | FBLN1 |
| 209223_at | 1.3099408 | NDUFA2 |
| 203719_at | 1.3098785 | ERCC1 |
| 1560648_s_at | 1.309681 | TSPYL1 |
| 243770_at | 1.3096782 | ZNF483 |
| 1565859_at | 1.309678 | SNORA71A |
| 1553561_at | 1.3096589 | TAS2R50 |
| 1554988_at | 1.3096589 | SLC9C2 |
| 231426_at | 1.3096589 |  |
| 242268_at | 1.3096589 | CELF2 |
| 241075_at | 1.3096588 | RIMKLA |
| 1558295_a_at | 1.3096516 | PPFIA2 |
| 233110_s_at | 1.3093902 | BCL2L12 |
| 212213_x_at | 1.3093226 | OPA1 |
| 213744_at | 1.3092862 | ATRNL1 |
| 213183_s_at | 1.3092551 | CDKN1C |
| 1562022_s_at | 1.3092296 | LOC100130987///RAD9A |
| 226680_at | 1.309153 | IKZF5 |
| 204030_s_at | 1.3090804 | IQCJ-SCHIP1///SCHIP1 |
| 203223_at | 1.3089617 | RABEP1 |
| 214959_s_at | 1.308912 | API5 |
| 235723_at | 1.3088986 | BNC2 |
| 1555829_at | 1.3088584 | ESYT2 |
| 1561846_s_at | 1.3088584 | CCDC168 |
| 215591_at | 1.3088584 | SATB2 |
| 209995_s_at | 1.3088104 | TCL1A |
| 209173_at | 1.3087792 | AGR2 |
| 239028_at | 1.3086503 | LYPD6 |
| 204872_at | 1.3085625 | TLE4 |
| 244835_at | 1.3084722 | C16orf52 |
| 1559256_at | 1.30846 | MAGI1 |
| 1560794_at | 1.30846 |  |
| 1565424_at | 1.30846 | LINC00529 |
| 213720_s_at | 1.30846 | SMARCA4 |
| 226304_at | 1.30846 | HSPB6 |
| 241252_at | 1.30846 | ESCO2 |
| 1560774_at | 1.3084598 |  |
| 205837_s_at | 1.308405 | GYPA |
| 210009_s_at | 1.3083013 | GOSR2 |
| 201926_s_at | 1.3081789 | CD55 |
| 227787_s_at | 1.308115 | MED30 |
| 226225_at | 1.3080311 | MCC |
| 224149_x_at | 1.307903 | SLMAP |
| 203627_at | 1.30784 | IGF1R |
| 239302_s_at | 1.3078002 | LOC100506922 |
| 202258_s_at | 1.3077847 | N4BP2L2 |
| 218877_s_at | 1.3076723 | TRMT11 |
| 220167_s_at | 1.3075656 | TP53TG3///TP53TG3B///TP53TG3C///TP53TG3D |
| 240421_x_at | 1.307547 | SAV1 |
| 229930_at | 1.3074087 | LOC100134361 |
| 1556471_at | 1.307354 | SCML4 |
| 209925_at | 1.3073167 | OCLN |
| 204243_at | 1.307209 | RLF |
| 237988_at | 1.3071909 | EIF1B |
| 220253_s_at | 1.3071611 | LRP12 |
| 202671_s_at | 1.3071175 | PDXK |
| 229823_at | 1.3071076 | RIMS2 |
| 214483_s_at | 1.3070678 | ARFIP1 |
| 1556195_a_at | 1.3070651 | OTTHUMG00000175735///RP4-798A10.7 |
| 1553491_at | 1.3070164 | KSR2 |
| 1563612_at | 1.3070164 |  |
| 236585_at | 1.3069118 | OTTHUMG00000184086///RP5-894A10.6 |
| 201881_s_at | 1.306879 | ARIH1 |
| 225673_at | 1.3066769 | MYADM |
| 202132_at | 1.3062576 | WWTR1 |
| 241858_at | 1.3061035 | TNNI3K |
| 229399_at | 1.306075 | C10orf118 |
| 203435_s_at | 1.3060465 | MME |
| 230278_at | 1.3060465 |  |
| 228835_at | 1.3059523 |  |
| 1554648_a_at | 1.3059522 | DUOXA1 |
| 201465_s_at | 1.3057996 | JUN |
| 209034_at | 1.305765 | PNRC1 |
| 227370_at | 1.3055476 | FAM171B |
| 230238_at | 1.3055141 | SOWAHA |
| 227052_at | 1.3052986 |  |
| 243285_at | 1.3052982 | LOC283335 |
| 201181_at | 1.3052576 | GNAI3 |
| 209555_s_at | 1.3051696 | CD36 |
| 225439_at | 1.3051333 | NUDCD1 |
| 203109_at | 1.3050505 | UBE2M |
| 228009_x_at | 1.3050402 | ZNRD1 |
| 216392_s_at | 1.3049763 | SEC23IP |
| 232500_at | 1.304963 | RALGAPA2 |
| 235429_at | 1.3049612 | EIF3E |
| 233780_at | 1.3049332 | RIF1 |
| 242489_at | 1.304883 | LOC646778 |
| 237693_at | 1.3046961 |  |
| 210943_s_at | 1.3046951 | LYST |
| 229485_x_at | 1.3046468 | SHISA3 |
| 1552312_a_at | 1.3046124 | MFAP3 |
| 1566821_at | 1.3044218 |  |
| 210127_at | 1.3042537 | RAB6B |
| 229393_at | 1.3041868 | L3MBTL3 |
| 206668_s_at | 1.3041313 | SCAMP1 |
| 235307_at | 1.303801 | LMTK2 |
| 1558803_at | 1.3038002 |  |
| 202156_s_at | 1.3038002 | CELF2 |
| 232404_at | 1.3038002 | SHROOM4 |
| 1558965_at | 1.3038 | PHF21A |
| 1563743_at | 1.3038 | C1orf180 |
| 239765_at | 1.3038 | CPEB3 |
| 228667_at | 1.3037279 | AGPAT4 |
| 218025_s_at | 1.3036038 | ECI2 |
| 210671_x_at | 1.3034332 | MAPK8 |
| 201412_at | 1.3034159 | LRP10 |
| 1554113_a_at | 1.3033729 | SLC4A8 |
| 1555469_a_at | 1.3033729 | CLASP2 |
| 243890_at | 1.3033317 |  |
| 205706_s_at | 1.3032901 | ANKRD26 |
| 1558920_at | 1.3031842 | SLC8A1-AS1 |
| 205651_x_at | 1.3031837 | RAPGEF4 |
| 235846_at | 1.3031058 | FSBP///RAD54B |
| 222838_at | 1.303088 | SLAMF7 |
| 206647_at | 1.3030877 | HBZ |
| 223502_s_at | 1.3030877 | TNFSF13B |
| 234218_at | 1.3030877 |  |
| 236038_at | 1.3030872 |  |
| 229338_at | 1.3030335 | LOC100289361 |
| 228664_at | 1.3029748 | LOC100507602 |
| 1555400_at | 1.3028551 | LOC645261 |
| 1559144_x_at | 1.3028551 | LINC00910 |
| 1559344_at | 1.3028551 | OTTHUMG00000161054///RP11-778J15.1 |
| 1562683_a_at | 1.3028551 | LOC285547 |
| 1562895_at | 1.3028551 | OTTHUMG00000020083///RP11-154D17.1 |
| 215481_s_at | 1.3028551 | PEX5 |
| 216947_at | 1.3028551 | DES |
| 220767_at | 1.3028551 |  |
| 238668_at | 1.3028551 |  |
| 228581_at | 1.3028549 | KCNJ10 |
| 1563085_at | 1.3028383 |  |
| 206411_s_at | 1.3028138 | ABL2 |
| 238740_at | 1.3028134 | PTGES3L |
| 222793_at | 1.3027424 | DDX58 |
| 238528_at | 1.3027424 | UBR1 |
| 242647_at | 1.3027424 | USP34 |
| 1556437_at | 1.3027419 | LOC253805 |
| 237276_at | 1.3027414 |  |
| 223624_at | 1.3024513 | ZFAND4 |
| 210654_at | 1.3024133 | TNFRSF10D |
| 228595_at | 1.302212 | HSD17B1 |
| 210350_x_at | 1.3022021 | ING1 |
| 203127_s_at | 1.3022003 | SPTLC2 |
| 211529_x_at | 1.302181 | HLA-G |
| 211814_s_at | 1.3020613 | CCNE2 |
| 214121_x_at | 1.3020613 | PDLIM7 |
| 238851_at | 1.3020557 | ANKRD13A |
| 203153_at | 1.3019991 | IFIT1 |
| 231970_at | 1.3018478 | GPATCH2L |
| 224120_at | 1.3018475 |  |
| 244644_at | 1.3018475 | FAM9C |
| 234785_at | 1.3018472 |  |
| 243856_at | 1.3018472 | LANCL3 |
| 1569213_at | 1.301847 | LOC400891 |
| 211544_s_at | 1.301847 | GHRHR |
| 220059_at | 1.301847 | STAP1 |
| 243025_at | 1.301847 |  |
| 231098_at | 1.3015859 |  |
| 1569905_at | 1.3015403 | HSD11B1L |
| 1557455_s_at | 1.3015394 | MOSPD1 |
| 219793_at | 1.3014942 | SNX16 |
| 231157_at | 1.3014913 | TTLL11 |
| 1555964_at | 1.301421 | ARL17A///ARL17B///LOC100294341///LOC100996709 |
| 240146_at | 1.3013903 |  |
| 1560898_at | 1.3013902 |  |
| 233963_at | 1.3013902 | H2BFXP |
| 240426_at | 1.3013902 | FAM47B |
| 210109_at | 1.3013884 | SND1-IT1 |
| 221277_s_at | 1.3013875 | PUS3 |
| 215092_s_at | 1.301207 | NFAT5 |
| 1552309_a_at | 1.3011549 | NEXN |
| 1562619_at | 1.3010684 | NME9 |
| 212496_s_at | 1.3009614 | KDM4B |
| 1557756_a_at | 1.3009558 | CEP128 |
| 201243_s_at | 1.3009541 | ATP1B1 |
| 218502_s_at | 1.3008822 | TRPS1 |
| 218695_at | 1.3008784 | EXOSC4 |
| 1556144_at | 1.3007976 | DHX30 |
| 224082_at | 1.3007597 |  |
| 1555176_at | 1.3006951 |  |
| 224707_at | 1.3005964 | CYSTM1 |
| 241396_at | 1.3005929 | NEDD4L |
| 229883_at | 1.3004313 | GRIN2D |
| 220795_s_at | 1.3004124 | BEGAIN |
| 239711_at | 1.3003918 | ADAL |
| 239903_at | 1.3003573 | TPBG |
| 214434_at | 1.3002263 | HSPA12A |
| 217615_at | 1.30011 |  |
| 216862_s_at | -1.3001666 | CMC4 |
| 219432_at | -1.300194 | EVC |
| 219717_at | -1.3002211 | DCAF16 |
| 233702_x_at | -1.3003006 |  |
| 234578_at | -1.3003926 |  |
| 203065_s_at | -1.3004593 | CAV1 |
| 205055_at | -1.3006239 | ITGAE |
| 1555392_at | -1.3006402 |  |
| 235064_s_at | -1.300648 | C20orf196 |
| 235742_at | -1.3006505 | RHOC |
| 231009_at | -1.3006718 | PLA2G12B |
| 229490_s_at | -1.3006952 |  |
| 203519_s_at | -1.3007666 | UPF2 |
| 242035_at | -1.3008327 | AC005534.9///OTTHUMG00000151508 |
| 207133_x_at | -1.3009081 | ALPK1 |
| 201888_s_at | -1.3011588 | IL13RA1 |
| 207165_at | -1.301194 | HMMR |
| 243136_at | -1.3011966 | GINS2 |
| 232312_at | -1.3012981 | PPP6R3 |
| 209709_s_at | -1.3013428 | HMMR |
| 1557558_s_at | -1.3013899 | MATN1-AS1 |
| 1554771_at | -1.3013902 | OTTHUMG00000176280///RP11-690I21.2 |
| 1569652_at | -1.3013902 | MLLT3 |
| 211356_x_at | -1.3013902 | LEPR |
| 1567703_at | -1.3013903 |  |
| 226356_at | -1.3014349 | FAM73B |
| 234255_at | -1.3014934 |  |
| 235386_at | -1.3014942 | OTTHUMG00000160053///RP11-774O3.3 |
| 210134_x_at | -1.3015157 | SHOX2 |
| 234788_x_at | -1.3015398 |  |
| 1557081_at | -1.3016418 | RBM25 |
| 208392_x_at | -1.3018178 | SP110 |
| 1553002_at | -1.3018467 | DEFB105A///DEFB105B |
| 236823_at | -1.3018467 | IDS |
| 238361_s_at | -1.3018467 |  |
| 1552825_at | -1.301847 | ZNF396 |
| 1560163_at | -1.301847 | OTTHUMG00000177715///RP11-75C10.6 |
| 1562787_at | -1.301847 |  |
| 1570222_at | -1.301847 | NDST4 |
| 234085_at | -1.301847 | CCDC169 |
| 237087_at | -1.301847 |  |
| 239632_at | -1.301847 |  |
| 217292_at | -1.3018475 | MTMR7 |
| 235999_at | -1.3018475 |  |
| 212959_s_at | -1.3019245 | GNPTAB |
| 221218_s_at | -1.3019985 | TPK1 |
| 214768_x_at | -1.3020613 | IGKV2-28///IGKV2-28///IGKV2D-28///IGKV2D-28 |
| 1556667_at | -1.3022946 | FTCDNL1 |
| 243801_x_at | -1.3022946 | MRPL30 |
| 234028_at | -1.3027416 |  |
| 234892_at | -1.3027421 |  |
| 230987_at | -1.3027424 |  |
| 229253_at | -1.3028129 | THEM4 |
| 231502_at | -1.3028549 |  |
| 217050_at | -1.3028551 | EPAG |
| 227412_at | -1.3028551 | PPP1R3E |
| 241613_at | -1.3028551 |  |
| 243646_at | -1.3028551 |  |
| 238140_at | -1.3028562 | ARV1 |
| 207485_x_at | -1.3030877 | BTN3A1 |
| 238353_at | -1.3030877 | RASL11A |
| 207424_at | -1.3031836 | MYF5 |
| 217380_s_at | -1.3031837 | ADD3-AS1 |
| 230772_at | -1.3031842 | HNF4A |
| 240542_at | -1.3031842 |  |
| 241723_at | -1.3031842 | IQGAP2 |
| 225529_at | -1.303331 | ACAP3 |
| 211002_s_at | -1.3034332 | TRIM29 |
| 204275_at | -1.3036641 | SOLH |
| 1560181_at | -1.303746 | LDLRAD4-AS1 |
| 232822_x_at | -1.3038002 | LINC00629 |
| 1556090_at | -1.3038007 |  |
| 231964_at | -1.3039072 | BICD1 |
| 218585_s_at | -1.3039123 | DTL |
| 219880_at | -1.3039558 | CTC-338M12.4///OTTHUMG00000163003 |
| 206428_s_at | -1.3040192 |  |
| 230258_at | -1.304087 | GLIS3 |
| 243891_at | -1.304108 |  |
| 1007_s_at | -1.3041158 | DDR1///MIR4640 |
| 204129_at | -1.3041382 | BCL9///LOC101060700 |
| 218308_at | -1.3043674 | TACC3 |
| 238477_at | -1.3044399 | KIF1C |
| 215507_x_at | -1.3045218 | RAB22A |
| 239658_at | -1.304665 |  |
| 239243_at | -1.3046961 | ZNF638///ZNF638-IT1 |
| 224938_at | -1.3047556 | NUFIP2 |
| 228323_at | -1.3048348 | CASC5 |
| 224917_at | -1.3048366 | MIR21///VMP1 |
| 212651_at | -1.3048836 | RHOBTB1 |
| 238364_x_at | -1.305018 | GLI4 |
| 207186_s_at | -1.3051989 | BPTF |
| 223681_s_at | -1.305249 | INADL |
| 205806_at | -1.3052869 | ROM1 |
| 242030_at | -1.3052871 |  |
| 243907_at | -1.3052878 |  |
| 1566609_at | -1.305387 |  |
| 204214_s_at | -1.3053888 | RAB32 |
| 238628_s_at | -1.3055423 | TRAPPC2L |
| 242253_at | -1.3056273 | PPP5D1 |
| 219565_at | -1.3057983 | CYP20A1 |
| 242012_at | -1.3057996 | OTTHUMG00000022519///RP11-308D16.4 |
| 213727_x_at | -1.3058708 | MPPE1 |
| 238756_at | -1.3059757 | GAS2L3 |
| 221098_x_at | -1.3060465 | UTP14A |
| 224860_at | -1.3060465 | C9orf123 |
| 220509_at | -1.3061032 | RBM26 |
| 214229_at | -1.3061035 | DNAH17 |
| 243347_at | -1.3061298 |  |
| 219798_s_at | -1.3063279 | MEPCE |
| 204381_at | -1.3064057 | LRP3 |
| 217429_at | -1.306473 |  |
| 205406_s_at | -1.3069183 | SPA17 |
| 206100_at | -1.3070164 | CPM |
| 215270_at | -1.3070164 | LFNG |
| 1554595_at | -1.307098 | SYMPK |
| 223348_x_at | -1.3071097 | MUM1 |
| 1559523_at | -1.3071909 |  |
| 209603_at | -1.3072228 | GATA3 |
| 206335_at | -1.3072401 | GALNS |
| 1554887_at | -1.3073007 | OTTHUMG00000157105///RP11-132A1.4 |
| 208037_s_at | -1.307343 | MADCAM1 |
| 206359_at | -1.3073534 | SOCS3 |
| 237603_at | -1.3073534 | C1orf100 |
| 204276_at | -1.3074406 | TK2 |
| 206273_at | -1.3075842 | SLMO1 |
| 1552286_at | -1.3075962 | ATP6V1E2///FLJ41757 |
| 204233_s_at | -1.3077717 | CHKA |
| 226270_at | -1.307789 | EXOC2 |
| 209642_at | -1.3078105 | BUB1 |
| 238794_at | -1.3078119 | SFR1 |
| 211351_at | -1.3078719 | KIF25-AS1 |
| 242270_at | -1.3079044 |  |
| 225167_at | -1.3080138 | FRMD4A |
| 209540_at | -1.3081919 | IGF1 |
| 223177_at | -1.3082551 | NT5DC1 |
| 227335_at | -1.3082689 | DIDO1 |
| 228976_at | -1.3083736 | ICOSLG |
| 238823_at | -1.3084598 | FMNL3 |
| 244164_at | -1.3084598 | FAM223B |
| 1558518_at | -1.30846 | TAB3 |
| 201313_at | -1.30846 | ENO2 |
| 205669_at | -1.30846 | NCAM2 |
| 214347_s_at | -1.30846 | DDC |
| 220696_at | -1.30846 |  |
| 236809_at | -1.30846 |  |
| 242498_x_at | -1.30846 |  |
| 243592_at | -1.3084602 | REV1 |
| 215067_x_at | -1.3084608 | PRDX2 |
| 218875_s_at | -1.3084608 | FBXO5 |
| 236315_at | -1.3088588 |  |
| 210674_s_at | -1.3088865 | PCDHA1///PCDHA10///PCDHA11///PCDHA12///PCDHA13///  PCDHA2///PCDHA3///PCDHA4///PCDHA5///PCDHA6///PCDHA7///  PCDHA8///PCDHA9///PCDHAC1///PCDHAC2 |
| 202870_s_at | -1.3088892 | CDC20 |
| 1559039_at | -1.3092551 | DHX36 |
| 212979_s_at | -1.3092833 | FAM115A///LOC100294033 |
| 237249_at | -1.3094975 | KCNQ1OT1 |
| 216921_s_at | -1.309498 | KRT35 |
| 228484_s_at | -1.3096585 | FOXO1 |
| 230181_at | -1.3096585 | PQLC1 |
| 232994_s_at | -1.3096585 | ARHGEF28 |
| 242256_x_at | -1.3096585 |  |
| 1557570_a_at | -1.3096588 | LOC285084 |
| 231585_at | -1.3096588 | VPS13A |
| 222535_at | -1.3096589 | INF2 |
| 237076_at | -1.3096589 | NCSTN |
| 1569264_at | -1.3096591 | LOC400655 |
| 205368_at | -1.3096591 | FAM131B |
| 219174_at | -1.3096591 | IFT74 |
| 224712_x_at | -1.3096776 | SMIM7 |
| 215069_at | -1.309678 | NMT2 |
| 233395_at | -1.309678 |  |
| 219800_s_at | -1.3096784 | THNSL1 |
| 1558135_at | -1.309681 | TAF11 |
| 243514_at | -1.3096812 |  |
| 210911_at | -1.3096814 | ID2B |
| 214268_s_at | -1.3101139 | MTMR4 |
| 214681_at | -1.310159 | GK |
| 242673_at | -1.3102525 |  |
| 206944_at | -1.3104427 | HTR6 |
| 225927_at | -1.3105955 | MAP3K1 |
| 236419_at | -1.3106652 |  |
| 1569503_at | -1.3107129 | HEATR5B |
| 237484_at | -1.3107129 | SMCO3 |
| 218876_at | -1.3107134 | TPPP3 |
| 211973_at | -1.3107619 |  |
| 212775_at | -1.3107619 | OBSL1 |
| 234128_at | -1.3107619 |  |
| 237495_at | -1.3107619 | MPP7 |
| 211079_s_at | -1.3109032 | DYRK1A |
| 244638_at | -1.3109032 | SUCLG1 |
| 1563635_at | -1.3109033 | AC093415.2///OTTHUMG00000155924 |
| 235275_at | -1.3109045 | BMP8B |
| 204822_at | -1.3109057 | TTK |
| 238888_at | -1.3109924 |  |
| 225066_at | -1.3110543 | PPP2R2D |
| 215553_x_at | -1.3111137 |  |
| 239478_x_at | -1.3111571 | GPATCH2L |
| 232633_at | -1.3111675 | XRCC5 |
| 240950_s_at | -1.3112065 | CCDC155 |
| 214114_x_at | -1.3112229 | FASTK |
| 211235_s_at | -1.3112698 | ESR1 |
| 244347_at | -1.3112698 |  |
| 1560512_at | -1.3112992 |  |
| 227502_at | -1.3112992 | OTTHUMG00000157537///RP5-894A10.2 |
| 239512_at | -1.3112992 | SRSF4 |
| 242077_x_at | -1.3113964 | MB21D1 |
| 1559620_at | -1.311419 | LOC441167 |
| 219213_at | -1.311424 | JAM2 |
| 213505_s_at | -1.31145 | SUGP2 |
| 1553118_at | -1.311665 | THEM4 |
| 226850_at | -1.3116945 | SUMF1 |
| 229225_at | -1.3117638 | NRP2 |
| 206169_x_at | -1.3117999 | ZC3H7B |
| 222207_x_at | -1.3120605 |  |
| 1558943_x_at | -1.312123 | ZNF765 |
| 227793_at | -1.3121425 | MIRLET7D |
| 203308_x_at | -1.3122089 | HPS1 |
| 233321_x_at | -1.3122617 | LOC90834 |
| 234675_x_at | -1.3125007 |  |
| 203481_at | -1.3125012 | FAM178A |
| 229821_at | -1.3125077 |  |
| 240166_x_at | -1.3125658 | TRMT10B |
| 217250_s_at | -1.3127915 | CHD5 |
| 211909_x_at | -1.3128579 | PTGER3 |
| 219765_at | -1.3128748 | ZNF329 |
| 238831_at | -1.3129199 | TMEM33 |
| 218324_s_at | -1.3130097 | SPATS2 |
| 228717_at | -1.3130388 |  |
| 1558537_x_at | -1.313062 | ZNF844 |
| 220668_s_at | -1.3131555 | DNMT3B |
| 237868_x_at | -1.3132302 |  |
| 207192_at | -1.3133396 | DNASE1L2 |
| 206698_at | -1.313409 | XK |
| 206759_at | -1.3134351 | FCER2 |
| 1570048_at | -1.3135123 | DNAJC24 |
| 214858_at | -1.3136871 | PP14571 |
| 242167_at | -1.3136878 |  |
| 244502_at | -1.3136878 |  |
| 1561346_at | -1.3136882 | OTTHUMG00000178827///RP11-433M22.2 |
| 1553248_at | -1.3138467 | CCDC57 |
| 240868_at | -1.3138782 | LOC100129406 |
| 223433_at | -1.3138832 | YAE1D1 |
| 231046_at | -1.3139797 | HP07349 |
| 229123_at | -1.3142141 |  |
| 1564151_at | -1.3144183 |  |
| 222637_at | -1.3144321 | COMMD10 |
| 1553533_at | -1.3145299 | JPH1 |
| 201792_at | -1.3145394 | AEBP1 |
| 1559993_at | -1.3146025 | SFXN3 |
| 211880_x_at | -1.3146974 | PCDHGA1 |
| 202175_at | -1.3149171 | CHPF |
| 221575_at | -1.3149171 | SCLY |
| 242514_at | -1.3149176 |  |
| 219559_at | -1.3149276 | SLC17A9 |
| 229033_s_at | -1.3152653 | MUM1 |
| 1559971_at | -1.315363 | BSDC1 |
| 236900_x_at | -1.315363 | CCDC163P |
| 216058_s_at | -1.3154894 | CYP2C19 |
| 217317_s_at | -1.3155378 | HERC2P2///HERC2P9 |
| 218908_at | -1.3155544 | ASPSCR1 |
| 244376_at | -1.3160998 | METTL7A |
| 233058_at | -1.3161145 | GPSM2 |
| 1554947_at | -1.3163421 | ACAT1 |
| 239511_s_at | -1.31652 | SRSF4 |
| 240297_at | -1.31652 |  |
| 218957_s_at | -1.3165487 | PAAF1 |
| 217610_at | -1.3167646 | SPDYE2 |
| 210560_at | -1.3168542 | GBX2 |
| 239036_at | -1.3170379 | AGO1 |
| 229333_at | -1.3171041 |  |
| 227069_at | -1.3171766 | CUX1 |
| 217342_x_at | -1.3172277 | FLJ11292 |
| 228697_at | -1.3172604 | HINT3 |
| 216321_s_at | -1.3173584 | NR3C1 |
| 213472_at | -1.3174967 | HNRNPH1 |
| 232306_at | -1.3174968 | CDH26 |
| 239617_at | -1.3174968 | GHRLOS |
| 230957_at | -1.3175471 | PCDHB19P |
| 1552875_a_at | -1.3175473 | CD200R1 |
| 1560842_a_at | -1.3175473 | TEX26-AS1 |
| 1561211_at | -1.3175473 | OTTHUMG00000007844///RP1-18D14.7 |
| 206581_at | -1.3175473 | BNC1 |
| 207291_at | -1.3175473 | PRRG4 |
| 216871_at | -1.3175473 |  |
| 227886_at | -1.3175473 | IFITM10 |
| 236493_at | -1.3175473 | NKAPP1 |
| 241193_at | -1.3175473 | ETS2 |
| 242820_at | -1.3175473 |  |
| 240685_at | -1.3175476 | LOC100505545 |
| 221310_at | -1.3175478 | FGF14 |
| 237762_at | -1.3175478 |  |
| 235830_at | -1.3175852 |  |
| 1556182_x_at | -1.3175918 | ANKRD65 |
| 222326_at | -1.3175926 |  |
| 229137_at | -1.3177923 | FUCA1 |
| 1560537_at | -1.317954 | FGF13-AS1 |
| 225601_at | -1.317975 | HMGB3 |
| 1556402_at | -1.3180101 |  |
| 226528_at | -1.3181249 | MTX3 |
| 218093_s_at | -1.3181999 | ANKRD10 |
| 231958_at | -1.318253 | TAMM41 |
| 209711_at | -1.3183445 | SLC35D1 |
| 213216_at | -1.3184582 | OTUD3 |
| 210992_x_at | -1.3184605 | FCGR2C |
| 232372_at | -1.3184605 |  |
| 1562669_at | -1.3184608 |  |
| 207113_s_at | -1.3184608 | TNF |
| 232692_at | -1.3184608 | TDRD6 |
| 243378_at | -1.3184611 |  |
| 204450_x_at | -1.3184613 | APOA1 |
| 225356_at | -1.31862 | LOC100996595 |
| 227804_at | -1.3188674 | TLCD1 |
| 1570210_x_at | -1.3189874 | PPP6R2 |
| 240108_at | -1.3190318 |  |
| 223566_s_at | -1.3190506 | BCOR |
| 242438_at | -1.3191042 | ASXL1 |
| 242298_x_at | -1.3191613 |  |
| 218210_at | -1.3192093 | FN3KRP |
| 1555842_at | -1.319231 | CYTH2 |
| 217684_at | -1.3192765 | TYMS |
| 234643_x_at | -1.3192912 |  |
| 226141_at | -1.3192965 | CCDC149 |
| 219843_at | -1.3193257 | IPP |
| 203145_at | -1.3193314 | SPAG5 |
| 204324_s_at | -1.3193662 | GOLIM4 |
| 208797_s_at | -1.3195573 | GOLGA8A |
| 235654_at | -1.3195575 | TMEM218 |
| 220458_at | -1.3195581 |  |
| 238860_at | -1.3195773 | OARD1 |
| 229305_at | -1.3197727 | MLF1IP |
| 239238_at | -1.3198531 |  |
| 225337_at | -1.3200397 | ABHD2 |
| 230045_at | -1.320059 | CNTN2 |
| 234949_at | -1.320059 | FRG1B |
| 1555617_x_at | -1.3200594 | AC010524.4///OTTHUMG00000183178 |
| 233605_x_at | -1.3200598 |  |
| 230018_at | -1.3201144 | DPP9 |
| 241681_at | -1.3204026 |  |
| 1556827_at | -1.3204027 | LPP-AS2 |
| 201249_at | -1.3204027 | SLC2A1 |
| 217197_x_at | -1.3204027 | N4BP2L1 |
| 239196_at | -1.3204027 | ANKRD22 |
| 241395_at | -1.3204032 | NIT1 |
| 1569312_at | -1.3204412 |  |
| 228549_at | -1.3204412 | OTTHUMG00000041221///RP11-285F7.2 |
| 210609_s_at | -1.3205774 | TP53I3 |
| 233358_at | -1.3206707 |  |
| 233929_x_at | -1.3207079 | LOC100288778///WASH1///WASH2P///WASH3P |
| 219990_at | -1.3207939 | E2F8 |
| 229797_at | -1.3208811 | MCOLN3 |
| 228318_s_at | -1.3209126 | CRIPAK |
| 223347_at | -1.3209178 | MUM1 |
| 216083_s_at | -1.3209711 | NEU3 |
| 226687_at | -1.3209711 | PRPF40A |
| 212821_at | -1.3210148 | PLEKHG3 |
| 219424_at | -1.3210506 | EBI3 |
| 215588_x_at | -1.3210554 | RIOK3 |
| 208022_s_at | -1.3211064 | CDC14B |
| 230457_at | -1.321223 | TMEM242 |
| 237107_at | -1.321223 | PRKRA |
| 244677_at | -1.3212233 | PER1 |
| 1557270_at | -1.3212235 |  |
| 229261_at | -1.3212235 |  |
| 230712_at | -1.3215175 | LOC101060378 |
| 220071_x_at | -1.3215204 | HAUS2 |
| 218263_s_at | -1.3215816 | ZBED5 |
| 231936_at | -1.3216192 | HOXC9 |
| 235701_at | -1.3216653 |  |
| 228209_at | -1.321939 | LOC100527964 |
| 234223_at | -1.3221565 |  |
| 215758_x_at | -1.3222265 | ZNF93 |
| 1568822_at | -1.3222737 | MTG2 |
| 212403_at | -1.3224905 | UBE3B |
| 215942_s_at | -1.3226501 | GTSE1 |
| 1560662_s_at | -1.322703 | WHAMMP2///WHAMMP3 |
| 228147_at | -1.322703 | SEPT7P2 |
| 231371_at | -1.322703 | TDRD10 |
| 237662_at | -1.3227547 |  |
| 204149_s_at | -1.3227755 | GSTM4 |
| 236891_at | -1.3228536 |  |
| 1555379_at | -1.3229012 | FAM159A |
| 1557116_at | -1.3230405 | APOL6 |
| 231774_at | -1.323135 | KCNIP3 |
| 36566_at | -1.3231686 | CTNS |
| 213605_s_at | -1.32317 |  |
| 233787_at | -1.3233696 | C6orf163 |
| 235304_at | -1.3233746 | LOC100507486 |
| 215741_x_at | -1.3235627 | AKAP8L |
| 234936_s_at | -1.3236691 | CC2D2A |
| 236475_at | -1.3237482 | MICAL2 |
| 215465_at | -1.3238407 | ABCA12 |
| 212921_at | -1.323847 | SMYD2 |
| 204886_at | -1.323869 | PLK4 |
| 1561392_at | -1.324018 |  |
| 216428_x_at | -1.3240182 | KIR3DX1 |
| 218839_at | -1.324079 | HEY1 |
| 236177_s_at | -1.3240908 |  |
| 207333_at | -1.3240911 | NMBR |
| 1554638_at | -1.3240913 | ZFYVE16 |
| 220676_at | -1.3240913 | ADAMTS8 |
| 239125_at | -1.3240913 | SLC25A5-AS1 |
| 231875_at | -1.3243276 | KIF21A |
| 244679_at | -1.3243754 |  |
| 1563397_at | -1.3245946 | AC114752.3///OTTHUMG00000152672 |
| 208588_at | -1.3245946 | FKSG2 |
| 225777_at | -1.3246831 | SAPCD2 |
| 1566129_at | -1.3247194 | LIMS1 |
| 214677_x_at | -1.3247199 | IGLC1 |
| 1558385_at | -1.3249125 |  |
| 244304_at | -1.324951 | DIS3L2 |
| 237229_at | -1.3251525 | CTD-3203P2.1///OTTHUMG00000177038 |
| 206555_s_at | -1.3252665 | THUMPD1 |
| 1557862_at | -1.3254743 | LOC654841 |
| 210528_at | -1.3254745 | MR1 |
| 232288_at | -1.3254745 | LOC101060433///PDXDC1///PDXDC2P |
| 232726_at | -1.3254745 |  |
| 206130_s_at | -1.325475 | ASGR2 |
| 239643_at | -1.325475 | LOC100129516 |
| 234731_at | -1.3254753 |  |
| 223463_at | -1.3256361 | RAB23 |
| 236494_x_at | -1.3256756 |  |
| 230676_s_at | -1.3256791 | TMEM19 |
| 205772_s_at | -1.3259225 |  |
| 210089_s_at | -1.3259225 | LAMA4 |
| 215762_at | -1.3259225 |  |
| 237316_at | -1.3259227 | ANKDD1A |
| 232457_at | -1.325983 | LIMCH1 |
| 1567320_at | -1.3259912 |  |
| 213629_x_at | -1.3259915 | MT1F |
| 1555935_s_at | -1.3260902 | HUNK |
| 1552579_a_at | -1.3261104 | ADAM21 |
| 211690_at | -1.3263357 | RPS6 |
| 244365_at | -1.3263357 |  |
| 235408_x_at | -1.3263359 | ZNF117 |
| 49452_at | -1.326469 | ACACB |
| 213641_at | -1.3264873 | ZNF500 |
| 207724_s_at | -1.3265123 | SPAST |
| 1556573_s_at | -1.3265136 | LOC286178 |
| 231871_at | -1.326554 | GPR180 |
| 209638_x_at | -1.3266364 | RGS12 |
| 226589_at | -1.3269447 | TMEM192 |
| 226004_at | -1.3272508 | CABLES2 |
| 221513_s_at | -1.3272688 | UTP14A///UTP14C |
| 204859_s_at | -1.3272899 | APAF1 |
| 243653_at | -1.3274093 | SHROOM3 |
| 230637_at | -1.3274492 | SFXN4 |
| 236076_at | -1.3275176 | LOC257396 |
| 228752_at | -1.3275949 | EFCAB4B |
| 222283_at | -1.3276075 | ZNF480 |
| 233114_at | -1.327762 |  |
| 205481_at | -1.3278744 | ADORA1 |
| 221580_s_at | -1.3282027 | MIR1304///SNORA1///SNORA18///SNORA32///SNORA40///  SNORA8///SNORD5///TAF1D |
| 1552903_at | -1.3282474 | B4GALNT2 |
| 231840_x_at | -1.3282474 | LYRM7 |
| 1553564_at | -1.3285688 | MACROD2 |
| 1559616_x_at | -1.3286444 | ZNF626 |
| 202095_s_at | -1.3287383 | BIRC5 |
| 224192_at | -1.3287437 | FCRL2 |
| 241360_at | -1.3287647 | CCDC15 |
| 238271_x_at | -1.3290173 |  |
| 238311_at | -1.3290173 |  |
| 225249_at | -1.3290178 | SPPL2B |
| 229155_at | -1.3290178 |  |
| 216524_x_at | -1.329316 |  |
| 231225_at | -1.3293165 |  |
| 228119_at | -1.3294975 | LRCH3 |
| 232058_at | -1.3295133 |  |
| 232529_at | -1.3295133 | SP3 |
| 220295_x_at | -1.3295603 | DEPDC1 |
| 237292_at | -1.3299625 | DPYSL3 |
| 226532_at | -1.3302108 |  |
| 239680_at | -1.3303995 | WDR76 |
| 215907_at | -1.3305802 |  |
| 228134_at | -1.3306317 | MYH11 |
| 218303_x_at | -1.3306321 | KRCC1 |
| 1570505_at | -1.3307478 | ABCB4 |
| 236224_at | -1.3307478 | RIT1 |
| 217339_x_at | -1.3307674 | CTAG1A///CTAG1B |
| 208539_x_at | -1.3308815 | SPRR2B |
| 207632_at | -1.3309348 | MUSK |
| 238024_at | -1.3309351 | AC100830.4///OTTHUMG00000178466 |
| 218902_at | -1.3309835 | NOTCH1 |
| 236390_at | -1.3309835 | SLX4IP |
| 201446_s_at | -1.3310785 | TIA1 |
| 201974_s_at | -1.3310829 | CCZ1 |
| 221250_s_at | -1.3310839 | MXD3 |
| 241131_at | -1.3311617 |  |
| 214751_at | -1.3311653 | ZNF468 |
| 237761_at | -1.3312128 |  |
| 218735_s_at | -1.3312734 | ZNF544 |
| 203046_s_at | -1.3312863 | TIMELESS |
| 215871_at | -1.3313705 | PLA2G5 |
| 243498_at | -1.3313705 |  |
| 235822_at | -1.3313707 | COQ9 |
| 232084_at | -1.3314649 | SGTB |
| 209116_x_at | -1.3315213 | HBB |
| 224170_s_at | -1.3315213 | TULP4 |
| 1568983_a_at | -1.3315283 |  |
| 236250_at | -1.3315533 | AFG3L1P |
| 215191_at | -1.3316092 |  |
| 216459_x_at | -1.3316092 | DDR1-AS1 |
| 241742_at | -1.3316092 | PRAM1 |
| 243379_at | -1.3316092 |  |
| 206056_x_at | -1.3316243 | SPN |
| 219653_at | -1.3316665 | LSM14B |
| 244871_s_at | -1.3317819 | USP32 |
| 244486_at | -1.3318443 |  |
| 234762_x_at | -1.3319645 | NLN |
| 213358_at | -1.3319887 | SOGA2 |
| 243538_at | -1.3319993 |  |
| 227125_at | -1.3320336 | IFNAR2 |
| 226794_at | -1.3320684 | STXBP5 |
| 229120_s_at | -1.3321965 | CDC42SE1 |
| 1566427_at | -1.3322341 |  |
| 213700_s_at | -1.3322604 | PKM |
| 219685_at | -1.3323727 | TMEM35 |
| 221459_at | -1.3323727 | TAAR5 |
| 1561640_at | -1.3323982 |  |
| 231873_at | -1.3325188 | BMPR2 |
| 1555996_s_at | -1.3326237 | EIF4A2///MIR1248///SNORA4///SNORA63///SNORA81///SNORD2 |
| 233106_at | -1.3326237 | FRMD6-AS1 |
| 230768_at | -1.3326765 |  |
| 202238_s_at | -1.3327223 | NNMT |
| 234136_at | -1.332747 |  |
| 242858_at | -1.332747 |  |
| 1565762_at | -1.3327641 |  |
| 233455_at | -1.3327641 |  |
| 229510_at | -1.3327646 | MS4A14 |
| 224610_at | -1.3327743 | SNHG1///SNORD22///SNORD25///SNORD26///SNORD27///SNORD28///SNORD29///SNORD31 |
| 218650_at | -1.3328607 | DGCR8///MIR1306 |
| 202207_at | -1.3329234 | ARL4C |
| 223710_at | -1.3329237 | CCL26 |
| 223815_at | -1.3329237 | CEP95 |
| 236873_at | -1.3329237 |  |
| 227367_at | -1.3329241 | SLCO3A1 |
| 242731_x_at | -1.3329241 |  |
| 207925_at | -1.3329668 | CST5 |
| 224254_x_at | -1.3329673 |  |
| 210251_s_at | -1.3329836 | RUFY3 |
| 221507_at | -1.333058 | TNPO2 |
| 223767_at | -1.3333954 | GPR84 |
| 240221_at | -1.3336283 | CSNK1A1 |
| 210603_at | -1.3337094 | NAA11 |
| 241434_at | -1.333917 | OTTHUMG00000085067///RP11-767N6.7 |
| 1566256_s_at | -1.3340154 | GPR180 |
| 204589_at | -1.3340793 | NUAK1 |
| 228670_at | -1.3341142 | TEP1 |
| 204826_at | -1.3343999 | CCNF |
| 222673_x_at | -1.334534 | FAM122B |
| 208323_s_at | -1.3345404 | ANXA13 |
| 211796_s_at | -1.3347352 | TRBC1 |
| 204326_x_at | -1.3347598 | MT1X |
| 212177_at | -1.3348287 | PNISR |
| 232215_x_at | -1.3349733 | PRR11 |
| 217253_at | -1.3349751 | SH3BP2 |
| 213900_at | -1.3350607 | FAM189A2 |
| 205037_at | -1.3351058 | IFT27 |
| 235893_at | -1.3351058 |  |
| 241014_at | -1.3351234 | FLG-AS1 |
| 232235_at | -1.3351535 | DSEL |
| 231467_at | -1.3352567 |  |
| 207444_at | -1.3352575 | SLC22A13 |
| 222276_at | -1.3352575 | METTL2B |
| 228175_at | -1.3353723 | SLC4A8 |
| 223307_at | -1.3353812 | CDCA3 |
| 226372_at | -1.3354341 | CHST11 |
| 1554271_a_at | -1.3354429 | CENPL |
| 1562012_at | -1.3354933 | LOC100506730 |
| 224015_s_at | -1.3356566 | MRPS25 |
| 220452_x_at | -1.3359352 | CECR7 |
| 218252_at | -1.3361812 | CKAP2 |
| 242546_at | -1.3366653 | FLJ39632///LOC100506303///LOC100653149///LOC101060483///  LOC400879///LOC440157 |
| 242633_x_at | -1.3367598 | ZNF75A |
| 209241_x_at | -1.3369025 | MINK1 |
| 226733_at | -1.3370541 | PFKFB2 |
| 240258_at | -1.3371301 | ENO1 |
| 214148_at | -1.337131 | ITFG2///LOC100507424 |
| 242051_at | -1.337131 |  |
| 209989_at | -1.3371655 | ZNF268 |
| 215037_s_at | -1.3373387 | BCL2L1 |
| 240441_at | -1.3377923 | FAM99B |
| 228466_at | -1.3381169 | GABPB2 |
| 226529_at | -1.3381593 | TMEM106B |
| 244601_at | -1.3382275 |  |
| 229134_at | -1.3383093 | VANGL1 |
| 209508_x_at | -1.3384522 | CFLAR |
| 213155_at | -1.3384522 | LOC339166///WSCD1 |
| 213565_s_at | -1.3384522 | SMAD6 |
| 220961_s_at | -1.3384522 | TBRG4 |
| 216550_x_at | -1.3385593 | ANKRD12 |
| 228882_at | -1.3388636 | TUB |
| 1555758_a_at | -1.3388832 | CDKN3 |
| 234723_x_at | -1.3389858 |  |
| 205970_at | -1.3390912 | MT3 |
| 1557347_at | -1.3393399 | MCPH1 |
| 241805_at | -1.3395312 | GABRG1 |
| 243114_at | -1.3395314 |  |
| 233854_x_at | -1.3395338 | KLK4 |
| 229747_x_at | -1.3397402 | LOC146880 |
| 1553443_at | -1.3397609 | FER1L6-AS1 |
| 237150_at | -1.3397609 |  |
| 1559293_x_at | -1.3397611 | LINC00032 |
| 1558600_a_at | -1.3398836 | CTB-58E17.7///OTTHUMG00000178506 |
| 244651_at | -1.3400278 |  |
| 244548_at | -1.340039 |  |
| 1568448_at | -1.3400937 |  |
| 203688_at | -1.3401263 | PKD2 |
| 1556421_at | -1.3401512 | LOC286189 |
| 235987_at | -1.3401512 | PRKXP1 |
| 219274_at | -1.3401884 | TSPAN12 |
| 1554212_s_at | -1.3403106 | KCNS2 |
| 220779_at | -1.3403115 | PADI3 |
| 227273_at | -1.340362 |  |
| 216289_at | -1.3403815 | GPR144 |
| 203710_at | -1.3404117 | ITPR1 |
| 239804_at | -1.34054 |  |
| 1565807_at | -1.3409181 |  |
| 232580_x_at | -1.341095 |  |
| 239400_at | -1.3411455 | FLJ45513 |
| 207730_x_at | -1.3413264 |  |
| 213599_at | -1.3413264 | OIP5 |
| 1563287_at | -1.3414254 | OTTHUMG00000160074///RP11-168E17.1 |
| 1566486_at | -1.3414257 |  |
| 217291_at | -1.3414257 | CEACAM5 |
| 223540_at | -1.3414257 | PVRL4 |
| 1563189_at | -1.3414259 |  |
| 1558508_a_at | -1.3414522 | C1orf53 |
| 233088_at | -1.3416407 | OTTHUMG00000177274///RP11-1102P22.1 |
| 1560758_at | -1.3416412 |  |
| 215200_x_at | -1.3417112 |  |
| 234898_at | -1.3417112 | SNAI1P1///SNAI1P1 |
| 236035_at | -1.3417112 | FZD1 |
| 236925_at | -1.3420463 | LOC100507568 |
| 1559045_at | -1.3420744 | LOC100128288 |
| 231252_at | -1.3422331 | KANSL1L |
| 209811_at | -1.3422333 | CASP2 |
| 214506_at | -1.3424147 | GPR182 |
| 204453_at | -1.3424671 | ZNF84 |
| 235576_at | -1.3426135 | WDR27 |
| 1559979_at | -1.342754 | SYF2 |
| 233995_at | -1.3427546 |  |
| 228454_at | -1.3428674 | LCOR |
| 235286_at | -1.3428807 |  |
| 239071_at | -1.3428824 | RBBP4 |
| 1558698_at | -1.3429037 | ZNF264 |
| 206623_at | -1.3429037 | PDE6A |
| 216067_at | -1.342904 |  |
| 233445_at | -1.3430405 |  |
| 55872_at | -1.3430493 | ZNF512B |
| 222500_at | -1.3432926 | PPIL1 |
| 204686_at | -1.3435369 | IRS1 |
| 235218_x_at | -1.3435577 | THAP6 |
| 223781_x_at | -1.3435688 | ADH4 |
| 219749_at | -1.3435777 | SH2D4A |
| 243049_at | -1.3436521 |  |
| 225534_at | -1.3436798 | SMIM19 |
| 1557126_a_at | -1.3437269 | PLD1 |
| 209032_s_at | -1.3437269 | CADM1 |
| 220714_at | -1.3437269 | PRDM14 |
| 237963_x_at | -1.3437269 |  |
| 243985_at | -1.3437269 | GTF2A2 |
| 218244_at | -1.3441702 | NOL8 |
| 1557963_at | -1.3442742 | CDC42BPB |
| 225017_at | -1.3442769 | CCDC14 |
| 226510_at | -1.3443533 | HEATR5A |
| 235565_at | -1.344447 | ZNF425 |
| 214718_at | -1.3444897 | GATAD1 |
| 228797_at | -1.3445275 | NLN |
| 212168_at | -1.344931 | RBM12 |
| 228357_at | -1.3452066 | UNK |
| 238578_at | -1.3453223 | TMEM182 |
| 241181_x_at | -1.3453227 |  |
| 232731_x_at | -1.3453816 | RAMP2-AS1 |
| 233659_at | -1.3453816 |  |
| 1556553_at | -1.3453819 |  |
| 1564084_at | -1.3453819 | CAD |
| 222046_at | -1.3453819 | SRRT |
| 242660_at | -1.3453819 | C10orf112 |
| 226139_at | -1.3458115 | CCDC149 |
| 205588_s_at | -1.3458488 | FGFR1OP |
| 227772_at | -1.3461816 | LATS1 |
| 227682_at | -1.3462497 |  |
| 218017_s_at | -1.3462502 | HGSNAT |
| 244491_at | -1.3462929 |  |
| 1554830_a_at | -1.3463151 | STEAP3 |
| 63825_at | -1.3465003 | ABHD2 |
| 220553_s_at | -1.3465216 | PRPF39 |
| 235595_at | -1.3465416 | ARHGEF2 |
| 240383_at | -1.346543 | UBE2D3 |
| 232557_at | -1.3465741 | UBAP1L |
| 235596_at | -1.346575 |  |
| 208160_at | -1.3468282 |  |
| 203001_s_at | -1.3469845 | STMN2 |
| 243394_at | -1.3472971 |  |
| 229366_at | -1.3473434 |  |
| 1566862_at | -1.3473474 |  |
| 204816_s_at | -1.3473479 | DHX34 |
| 1570270_at | -1.3473781 | OTTHUMG00000018731///RP11-15K3.1 |
| 206542_s_at | -1.3473781 | SMARCA2 |
| 229580_at | -1.3473781 | OTTHUMG00000175814///RP11-13L2.4 |
| 230366_at | -1.3473781 | LOC100505683 |
| 236752_at | -1.3473781 |  |
| 237410_x_at | -1.3473786 |  |
| 232266_x_at | -1.3473835 | CDK13 |
| 1559436_x_at | -1.34753 |  |
| 1559265_at | -1.34755 | SKIDA1 |
| 224747_at | -1.3475598 | UBE2Q2 |
| 207213_s_at | -1.347754 | USP2 |
| 243416_at | -1.347754 |  |
| 243751_at | -1.347754 | CHD2///LOC100507217 |
| 226062_x_at | -1.3477737 | FAM63A |
| 244427_at | -1.3477978 | KIF23 |
| 203959_s_at | -1.3480463 | ZBTB40 |
| 234076_at | -1.3481101 |  |
| 223541_at | -1.3481109 | HAS3 |
| 232147_at | -1.348126 | SLX4 |
| 236251_at | -1.3483016 |  |
| 214678_x_at | -1.3484045 | ZFX |
| 1552631_a_at | -1.3484688 | MAP3K6 |
| 230952_at | -1.34859 | OTTHUMG00000176111///RP11-426C22.5 |
| 239721_at | -1.3485909 |  |
| 202771_at | -1.3486131 | PIEZO1 |
| 206792_x_at | -1.3486828 | LOC729966///PDE4C |
| 206858_s_at | -1.3488379 | HOXC6 |
| 204396_s_at | -1.348882 | GRK5 |
| 230562_at | -1.3493694 | LOC100507530 |
| 237105_at | -1.3495014 | AC009948.5///OTTHUMG00000154423 |
| 205225_at | -1.3495905 | ESR1 |
| 228925_at | -1.3497467 | ADAM1A///ADAM1A |
| 235526_at | -1.3499298 | SOX6 |
| 231187_at | -1.34993 | SLC28A1 |
| 242669_at | -1.3503634 | UFM1 |
| 1563081_at | -1.3506743 |  |
| 218755_at | -1.3507438 | KIF20A |
| 231892_at | -1.3507643 | ARHGEF39 |
| 236903_at | -1.3507643 |  |
| 239131_at | -1.3508364 |  |
| 239312_at | -1.3508364 |  |
| 1554871_at | -1.3508366 |  |
| 217616_at | -1.3508366 |  |
| 222316_at | -1.3508366 |  |
| 236582_at | -1.3508366 |  |
| 242889_x_at | -1.3508366 | FUT8-AS1 |
| 243393_at | -1.3508366 |  |
| 243216_x_at | -1.3510027 |  |
| 237472_at | -1.3510188 | SOX1 |
| 236679_x_at | -1.351058 |  |
| 218369_s_at | -1.3510965 | EXOSC1 |
| 227714_s_at | -1.3513296 |  |
| 232034_at | -1.3515512 | LINC00537 |
| 221501_x_at | -1.3516226 | NPIPA2///NPIPA3///NPIPA5///PKD1P1 |
| 221313_at | -1.3520283 | GPR52 |
| 1557817_a_at | -1.3520479 | OTTHUMG00000016921///RP11-432M24.4 |
| 242111_at | -1.3521369 | METTL3 |
| 205178_s_at | -1.3522142 | RBBP6 |
| 203513_at | -1.3522191 | SPG11 |
| 1569999_at | -1.3522435 |  |
| 217446_x_at | -1.3524475 |  |
| 244825_at | -1.3525012 | SHROOM4 |
| 205846_at | -1.3526436 | PTPRB |
| 231162_at | -1.3527336 | CLDND2 |
| 244054_at | -1.3527336 |  |
| 225129_at | -1.352743 | CPNE2 |
| 203901_at | -1.352789 | TAB1 |
| 226520_at | -1.3528503 | LCOR |
| 242710_at | -1.3529114 |  |
| 204084_s_at | -1.3529831 | CLN5 |
| 228242_at | -1.3530283 | N4BP2 |
| 228398_at | -1.3531281 | SENP8 |
| 226426_at | -1.3531835 | ADNP |
| 227866_at | -1.3534058 | LOC100505519///TIAM2 |
| 205074_at | -1.3534967 | SLC22A5 |
| 227013_at | -1.3535253 | LATS2 |
| 237039_at | -1.3536747 | LOC100506088 |
| 237468_at | -1.3537121 |  |
| 1554834_a_at | -1.3537292 | RASSF5 |
| 216445_at | -1.3540962 | PRDM2 |
| 231114_at | -1.3542097 | SPATA22 |
| 235237_at | -1.3543818 | VMA21 |
| 219906_at | -1.3543859 | EBLN2 |
| 225926_at | -1.354555 | VTI1B |
| 235393_at | -1.3546647 |  |
| 242424_at | -1.3546647 |  |
| 213995_at | -1.3546866 | ATP5S |
| 237750_at | -1.3547393 | XPNPEP3 |
| 231030_at | -1.354782 | ZRANB2-AS1 |
| 1556283_s_at | -1.3548179 | FGFR1OP2 |
| 1556212_x_at | -1.3549012 |  |
| 1556111_s_at | -1.3549517 |  |
| 207798_s_at | -1.3549522 | ATXN2L |
| 211353_at | -1.354991 | LRIT1 |
| 214645_at | -1.3549912 |  |
| 201610_at | -1.3549916 | ICMT |
| 241402_at | -1.3550136 | TSEN54 |
| 240839_at | -1.3550256 |  |
| 1558421_a_at | -1.3552281 | C14orf180 |
| 210505_at | -1.3552285 | ADH7 |
| 242182_x_at | -1.3552568 |  |
| 233428_at | -1.3553954 |  |
| 224443_at | -1.3554848 | LINC00467 |
| 227474_at | -1.3556976 | LOC654433 |
| 235716_at | -1.3557988 |  |
| 1561775_at | -1.3558276 |  |
| 1555906_s_at | -1.3559539 | TCAIM |
| 214935_at | -1.355997 | IL4I1///NUP62 |
| 230752_at | -1.355997 |  |
| 1559711_at | -1.3559974 | FAM120A |
| 236502_at | -1.3560355 |  |
| 205676_at | -1.3561529 | CYP27B1 |
| 205082_s_at | -1.3561538 | AOX1 |
| 40560_at | -1.3562121 | TBX2 |
| 228511_s_at | -1.3566308 | OTTHUMG00000171360///RP11-998D10.7 |
| 236241_at | -1.3566371 | MED31 |
| 204871_at | -1.3566774 | MTERF |
| 202326_at | -1.3571049 | EHMT2 |
| 1565621_at | -1.3571484 |  |
| 231449_at | -1.3571492 |  |
| 222820_at | -1.357152 | TNRC6C |
| 213455_at | -1.3574408 | FAM114A1 |
| 202701_at | -1.3574413 | BMP1 |
| 211198_s_at | -1.3575077 | ICOSLG |
| 222561_at | -1.3576514 | LANCL2 |
| 215063_x_at | -1.3579924 | LRRC40 |
| 204807_at | -1.3580099 | TMEM5 |
| 1555157_at | -1.3582281 |  |
| 220242_x_at | -1.3582281 | ZNF701 |
| 1557744_at | -1.3582283 |  |
| 241727_x_at | -1.3583771 | DHFRL1 |
| 1556800_a_at | -1.3584341 |  |
| 238130_at | -1.3585324 | NFATC2IP |
| 1555028_at | -1.3586879 | BRD3 |
| 232026_at | -1.3588262 | HERC4 |
| 215503_at | -1.3591107 | SPINT3 |
| 234429_at | -1.359111 | LINC00476 |
| 235845_at | -1.359111 | SP5 |
| 203737_s_at | -1.359172 | PPRC1 |
| 216983_s_at | -1.3592232 | ZNF224 |
| 242725_at | -1.3592232 |  |
| 201887_at | -1.3594488 | IL13RA1 |
| 1568815_a_at | -1.3594865 | DDX50 |
| 241865_at | -1.3597705 |  |
| 1554007_at | -1.3597885 |  |
| 1553984_s_at | -1.359828 | DTYMK |
| 1563130_a_at | -1.3599532 |  |
| 239991_at | -1.3600402 |  |
| 234399_at | -1.360111 | TRAV34///TRAV34 |
| 1552911_at | -1.3601112 | SIGLEC11 |
| 226811_at | -1.3601115 | FAM46C |
| 1555623_at | -1.3602417 |  |
| 242113_at | -1.3605886 |  |
| 225479_at | -1.3606571 | LRRC58 |
| 222922_at | -1.3606832 | KCNE3 |
| 239780_at | -1.3606832 |  |
| 211326_x_at | -1.3606868 | HFE |
| 226032_at | -1.360774 | CASP2 |
| 209271_at | -1.3609179 | BPTF |
| 213974_at | -1.3610379 | ADAMTSL3 |
| 228234_at | -1.3611038 | TICAM2///TMED7-TICAM2 |
| 229516_at | -1.3612446 | WDR31 |
| 222026_at | -1.3612796 | RBM3 |
| 238949_at | -1.36133 | RNF145 |
| 223906_s_at | -1.3615892 | TEX101 |
| 239333_x_at | -1.3616046 |  |
| 205046_at | -1.3617134 | CENPE |
| 212703_at | -1.3617332 | TLN2 |
| 224062_x_at | -1.3619655 | KLK4 |
| 228960_at | -1.3619655 | NARG2 |
| 233161_at | -1.3622392 |  |
| 229047_at | -1.3623873 | PLEKHB1 |
| 1557736_at | -1.3623878 | NKTR |
| 1558560_s_at | -1.3623878 | BLZF1 |
| 215109_at | -1.3623878 | RC3H1 |
| 217655_at | -1.362388 | LOC100127972 |
| 1559804_at | -1.3623883 | CTD-3046C4.1///OTTHUMG00000164394 |
| 206384_at | -1.3625371 | CACNG3 |
| 242398_x_at | -1.3625972 |  |
| 1559490_at | -1.3627381 | LRCH3 |
| 210221_at | -1.3627391 | CHRNA3 |
| 209527_at | -1.3629057 | EXOSC2 |
| 237511_at | -1.3632835 |  |
| 238574_at | -1.3632838 | SLC25A51 |
| 236220_at | -1.3633779 |  |
| 233835_at | -1.3635167 | LOC90246 |
| 222584_at | -1.3635225 | MSTO1 |
| 213965_s_at | -1.36377 | CHD5 |
| 236869_at | -1.3639332 |  |
| 242492_at | -1.363995 | CLNS1A |
| 211935_at | -1.3640486 | ARL6IP1 |
| 231236_at | -1.3642046 | ZFP57 |
| 215179_x_at | -1.3642722 | PGF |
| 201458_s_at | -1.3643228 | BUB3 |
| 233427_x_at | -1.3644495 |  |
| 223380_s_at | -1.3644832 | LATS2 |
| 228904_at | -1.3647376 | HOXB3 |
| 222762_x_at | -1.3647611 | LIMD1 |
| 242337_at | -1.3647783 |  |
| 203002_at | -1.3649181 | AMOTL2 |
| 212705_x_at | -1.3649709 | PNPLA2 |
| 238807_at | -1.365071 | ANKRD46///GAPDHP62 |
| 228868_x_at | -1.3651075 | CDT1 |
| 221645_s_at | -1.3652647 | ZNF83 |
| 226064_s_at | -1.3652664 | DGAT2 |
| 1558120_at | -1.3652804 | DDX3X |
| 242734_x_at | -1.3652804 | GALT |
| 230363_s_at | -1.3652809 | INPP5F |
| 226150_at | -1.3653314 | PPAPDC1B |
| 229692_at | -1.3654578 |  |
| 203678_at | -1.3655169 | FAN1 |
| 209714_s_at | -1.3656979 | CDKN3 |
| 1558021_at | -1.3656993 | RABEPK |
| 237065_s_at | -1.366106 | LAMTOR5-AS1 |
| 212981_s_at | -1.3661313 | FAM115A///LOC100294033 |
| 1569839_s_at | -1.3662794 | LRRC37A5P |
| 229454_at | -1.3663579 | BCLAF1 |
| 233236_at | -1.3663833 | TSPAN16 |
| 226685_at | -1.3664329 | SNTB2 |
| 207314_x_at | -1.3665422 | KIR3DL2///LOC727787 |
| 227839_at | -1.3665513 | MBD5 |
| 222805_at | -1.3667148 | MANEA |
| 236590_at | -1.3668259 |  |
| 243495_s_at | -1.366835 | ZNF652 |
| 216621_at | -1.3668824 |  |
| 229480_at | -1.3668824 | MAGI2-AS3 |
| 232468_at | -1.3668824 |  |
| 236879_at | -1.3668824 |  |
| 236027_at | -1.3669732 | SFR1 |
| 209386_at | -1.3671213 | TM4SF1 |
| 1552829_at | -1.367143 |  |
| 207383_s_at | -1.3672036 | RHBDL1 |
| 1556173_a_at | -1.367331 |  |
| 1566178_x_at | -1.3673315 | CLYBL-IT1 |
| 244784_at | -1.3674564 | DHX57 |
| 239616_at | -1.3674569 | REXO2 |
| 240078_at | -1.3674569 | SFSWAP |
| 209858_x_at | -1.3675684 | MPPE1 |
| 243682_at | -1.3675833 |  |
| 209119_x_at | -1.3675896 | NR2F2 |
| 1562288_at | -1.3677826 | AL592494.5///OTTHUMG00000013092 |
| 1566268_at | -1.3677828 |  |
| 234479_at | -1.3677828 | PCDHB18 |
| 219495_s_at | -1.3679934 | ZNF180 |
| 216713_at | -1.3685138 | KRIT1 |
| 235418_at | -1.3685635 | FAHD2A |
| 215600_x_at | -1.368602 | FBXW12 |
| 222816_s_at | -1.3686405 | ZCCHC2 |
| 242463_x_at | -1.368868 | ZNF600 |
| 206565_x_at | -1.3691952 | SMA4 |
| 235425_at | -1.3692558 | SGOL2 |
| 1554894_a_at | -1.3693373 | PCBD2 |
| 206134_at | -1.3693557 | ADAMDEC1 |
| 222073_at | -1.3693701 | COL4A3 |
| 217244_at | -1.3694255 | DNAJA1P4///DNAJA1P4 |
| 1565073_at | -1.3694563 | OTTHUMG00000179668///RP11-118G23.1 |
| 241488_at | -1.3694568 |  |
| 1553211_at | -1.3695029 | ANKFN1 |
| 1554659_at | -1.3695029 | NEK4 |
| 239442_at | -1.3695029 | CEP68 |
| 205359_at | -1.3695031 | AKAP6 |
| 1559437_at | -1.3695204 |  |
| 206064_s_at | -1.3695238 | PPIL2 |
| 232532_at | -1.369617 | QRICH2 |
| 1554177_a_at | -1.3698438 | ATP5S |
| 219502_at | -1.3702084 | NEIL3 |
| 228181_at | -1.370303 | SLC30A1 |
| 215455_at | -1.3705245 | TIMELESS |
| 205340_at | -1.3705454 | ZBTB24 |
| 228594_at | -1.3705666 | NADK2 |
| 219632_s_at | -1.3706654 | SHPK///TRPV1 |
| 213405_at | -1.3709023 | RAB22A |
| 210960_at | -1.3713086 | ADRA1D |
| 224477_s_at | -1.3713429 | NUDT16L1 |
| 225942_at | -1.3714026 | NLN |
| 234030_at | -1.3716298 |  |
| 214136_at | -1.3716583 | NUDT13 |
| 236120_at | -1.3716586 |  |
| 1560445_x_at | -1.3718252 | ARHGEF1 |
| 81811_at | -1.3720573 |  |
| 1556048_at | -1.372198 | LOC100507564 |
| 1556597_a_at | -1.3722423 | LOC284513 |
| 37577_at | -1.3724293 | ARHGAP19 |
| 217627_at | -1.3724637 | ZNF573 |
| 219579_at | -1.3725817 | RAB3IL1 |
| 239815_at | -1.3728303 |  |
| 235467_s_at | -1.3729792 | KCNC4 |
| 244659_at | -1.373324 | LOC100131015 |
| 204773_at | -1.3734021 | IL11RA |
| 233511_at | -1.3735728 | LOC100653233 |
| 223000_s_at | -1.3743358 | F11R |
| 1554356_at | -1.3744256 | GINS4 |
| 221436_s_at | -1.3746338 | CDCA3 |
| 215190_at | -1.3746393 | EIF3M |
| 1559987_at | -1.3747842 |  |
| 231886_at | -1.3750772 | LOC100134822 |
| 226942_at | -1.3752046 | PHF20L1 |
| 220749_at | -1.3752118 | C10orf68 |
| 205698_s_at | -1.3753196 | MAP2K6 |
| 210865_at | -1.3755671 | FASLG |
| 226829_at | -1.3755673 | AFAP1L2 |
| 208082_x_at | -1.3758247 | MKRN4P///MKRN4P |
| 226580_at | -1.3759229 | BRMS1L |
| 236486_at | -1.3759748 |  |
| 209459_s_at | -1.3761082 | ABAT |
| 229323_at | -1.3761082 | LINC00959 |
| 243849_at | -1.3761084 | TMEM37 |
| 218351_at | -1.3762221 | COMMD8 |
| 204403_x_at | -1.3766775 | FAM115A///LOC100294033 |
| 225944_at | -1.3768495 | NLN |
| 204828_at | -1.3768632 | RAD9A |
| 222280_at | -1.3769329 | LOC100506469 |
| 222152_at | -1.3770403 | PDCD6 |
| 231882_at | -1.377124 | FLJ39632 |
| 208538_at | -1.3771987 | ANP32C |
| 220546_at | -1.3771987 | KMT2A |
| 1559094_at | -1.3771989 | FBXO9 |
| 240215_at | -1.377199 |  |
| 239698_at | -1.3771993 |  |
| 222360_at | -1.3772233 | DPH5 |
| 241792_x_at | -1.3774896 | LUC7L3 |
| 1559429_a_at | -1.3776523 | C3orf43 |
| 239906_at | -1.3778042 |  |
| 211645_x_at | -1.378042 | IGKV1-17///IGKV1-17 |
| 233214_at | -1.3785185 |  |
| 233775_x_at | -1.3785563 | LOC100289333 |
| 220305_at | -1.3785626 | MAVS |
| 231825_x_at | -1.3786451 | ATF7IP |
| 1554816_at | -1.3787448 | ASTN2 |
| 216527_at | -1.3787448 | HCG18 |
| 211372_s_at | -1.3787451 | IL1R2 |
| 221155_x_at | -1.3787453 |  |
| 227157_at | -1.378914 | CCDC111 |
| 244419_at | -1.378967 | FRZB |
| 1560917_at | -1.3789673 |  |
| 229386_at | -1.3789678 | ID4 |
| 215018_at | -1.378968 | KIAA1731 |
| 242110_at | -1.378968 |  |
| 201110_s_at | -1.379038 | THBS1 |
| 231515_at | -1.3792467 | CTD-2286N8.2///OTTHUMG00000180261 |
| 220855_at | -1.3794993 | CLTC-IT1 |
| 234697_x_at | -1.3794993 | TAMM41 |
| 240447_at | -1.3794993 | HYI-AS1///HYI-AS1 |
| 1568598_at | -1.3796762 | KAZALD1 |
| 226370_at | -1.3797737 | KLHL15 |
| 241933_at | -1.3802335 | QRSL1 |
| 1552291_at | -1.380908 | PIGX |
| 1554250_s_at | -1.380908 | TRIM73 |
| 1566931_at | -1.3812402 | TFB2M |
| 240673_at | -1.3812476 |  |
| 227966_s_at | -1.3818074 | CCDC74A///CCDC74B |
| 1555488_at | -1.3818997 |  |
| 217032_at | -1.3818997 | FOXD4///FOXD4L1 |
| 217856_at | -1.3819339 | RBM8A |
| 242108_at | -1.3819736 |  |
| 202437_s_at | -1.3820696 | CYP1B1 |
| 234700_s_at | -1.3822523 | RNASE7 |
| 234756_at | -1.3822523 | CACNG8 |
| 1558280_s_at | -1.3823179 | ARHGAP29 |
| 213245_at | -1.3824899 | ADCY1 |
| 219915_s_at | -1.3824899 | SLC16A10 |
| 215290_at | -1.3825183 |  |
| 234981_x_at | -1.3825657 | CMBL |
| 230686_s_at | -1.382723 | SLC13A3 |
| 244695_at | -1.382723 | GHRLOS |
| 243857_at | -1.3827741 | MORF4L2 |
| 204451_at | -1.3829278 | FZD1 |
| 1555938_x_at | -1.3833508 | VIM |
| 218109_s_at | -1.3839968 | MFSD1 |
| 212023_s_at | -1.3841958 | MKI67 |
| 209446_s_at | -1.3842498 |  |
| 1570252_at | -1.3843461 |  |
| 1561015_at | -1.3843464 |  |
| 240105_at | -1.3843464 |  |
| 222039_at | -1.3845413 | KIF18B |
| 204136_at | -1.3845903 | COL7A1 |
| 210425_x_at | -1.3852242 | GOLGA8A///GOLGA8B |
| 225626_at | -1.3853487 | PAG1 |
| 216694_at | -1.3857435 |  |
| 210920_x_at | -1.3859235 |  |
| 231576_at | -1.385968 |  |
| 240230_s_at | -1.3859862 | AGAP9 |
| 234251_at | -1.3861865 |  |
| 239387_at | -1.3862091 |  |
| 206108_s_at | -1.3863161 | SRSF6 |
| 209788_s_at | -1.3863225 | ERAP1 |
| 229413_s_at | -1.3863714 |  |
| 216026_s_at | -1.386439 | POLE |
| 204318_s_at | -1.3867029 | GTSE1 |
| 236032_at | -1.3867836 |  |
| 233329_s_at | -1.3871558 | KRCC1 |
| 1552458_at | -1.3873899 | MBD3L1 |
| 205168_at | -1.387486 | DDR2 |
| 228877_at | -1.3877226 | RGL3 |
| 239045_at | -1.3877226 |  |
| 242369_x_at | -1.3877914 |  |
| 1553232_at | -1.3877916 | RMDN2 |
| 225928_at | -1.3879969 | VTI1B |
| 202532_s_at | -1.3881024 | DHFR |
| 221909_at | -1.3882071 | RNFT2 |
| 203052_at | -1.3882277 | C2 |
| 207189_s_at | -1.3884553 | ZZEF1 |
| 1570441_at | -1.3884555 | NAPB |
| 216788_at | -1.3884557 |  |
| 229687_s_at | -1.3884557 | PRDM11 |
| 237020_at | -1.3884557 | CATSPERD |
| 1559957_a_at | -1.3889377 | LOC642852 |
| 241779_at | -1.3889427 | MTX3 |
| 202330_s_at | -1.3889762 | UNG |
| 230213_at | -1.3889881 | C19orf43 |
| 206928_at | -1.3891002 | ZNF124 |
| 211864_s_at | -1.3893913 | MYOF |
| 1558687_a_at | -1.3893918 | FOXN1 |
| 236255_at | -1.3894124 | PLEKHG4B |
| 207090_x_at | -1.3895267 | ZFP30 |
| 220623_s_at | -1.3898791 | TSGA10 |
| 216958_s_at | -1.3900206 | IVD |
| 1561650_s_at | -1.3900523 | LOC285692 |
| 223596_at | -1.3900523 | SLC12A6 |
| 237383_at | -1.3900523 |  |
| 1566513_a_at | -1.3901219 | GNG4 |
| 202450_s_at | -1.3901258 | CTSK |
| 1563706_at | -1.3902565 |  |
| 218421_at | -1.3902925 | CERK |
| 244145_at | -1.3903155 |  |
| 1562297_at | -1.3907788 |  |
| 230360_at | -1.390779 | GLDN |
| 242374_at | -1.390779 |  |
| 206188_at | -1.3911628 | ZNF623 |
| 237003_at | -1.3911705 | BEST3 |
| 236678_at | -1.3911707 |  |
| 230256_at | -1.3913361 | RUSC1-AS1 |
| 215754_at | -1.3914418 | SCARB2 |
| 228616_at | -1.3914418 | POLRMT |
| 201289_at | -1.391647 | CYR61 |
| 232753_at | -1.391859 | ZNF346 |
| 225961_at | -1.3919083 | KLHL42 |
| 226340_x_at | -1.3919727 | LOC100288778///WASH1///WASH2P///WASH3P///WASH7P |
| 204808_s_at | -1.3926464 | TMEM5 |
| 240049_at | -1.3927304 | OTTHUMG00000018545///RP13-39P12.3 |
| 234958_at | -1.3927587 |  |
| 210115_at | -1.3930736 | RPL39L |
| 240592_at | -1.3933108 | LCORL |
| 1568639_a_at | -1.3934954 |  |
| 227500_at | -1.3936847 | FBXL18 |
| 1569219_at | -1.3936914 |  |
| 242239_at | -1.393876 | ARL5B-AS1 |
| 207228_at | -1.3939966 | PRKACG |
| 229795_at | -1.3941367 |  |
| 48808_at | -1.394146 | DHFR |
| 225760_at | -1.3944592 | MYSM1 |
| 229354_at | -1.3945787 | AHRR |
| 227195_at | -1.3946861 | ZNF503 |
| 214176_s_at | -1.3948336 | PBXIP1 |
| 228940_at | -1.3948336 | NDUFB4 |
| 240725_at | -1.3948336 |  |
| 243683_at | -1.3948336 | MORF4L2 |
| 243730_at | -1.3948336 |  |
| 212738_at | -1.3948963 | ARHGAP19 |
| 221176_x_at | -1.3949202 |  |
| 203660_s_at | -1.3950673 | PCNT |
| 242539_at | -1.3950784 | DIS3L2 |
| 243759_at | -1.3950849 | SCAF4 |
| 222773_s_at | -1.3955972 | GALNT12 |
| 1559360_at | -1.395707 |  |
| 215435_at | -1.3960768 |  |
| 216025_x_at | -1.3960773 | CYP2C9 |
| 243158_at | -1.3960773 |  |
| 1554203_at | -1.3962076 | GRIK1-AS1 |
| 209291_at | -1.396221 | ID4 |
| 1555702_a_at | -1.3963484 | ST3GAL3 |
| 1553372_at | -1.3963493 |  |
| 205251_at | -1.3964444 | PER2 |
| 231568_at | -1.396689 | CT47A1///CT47A10///CT47A11///CT47A12///CT47A2///CT47A3///  CT47A4///CT47A5///CT47A6///CT47A7///CT47A8///CT47A9 |
| 227822_at | -1.3967288 | ZNF605 |
| 222890_at | -1.39693 | CCDC113 |
| 236380_at | -1.3970025 |  |
| 1569487_at | -1.3973105 |  |
| 1562474_at | -1.3978163 | OTTHUMG00000012342///RP11-418J17.1 |
| 1558220_at | -1.3979092 |  |
| 243016_at | -1.3979498 |  |
| 242024_at | -1.3980042 |  |
| 215068_s_at | -1.3981768 | FBXL18 |
| 201542_at | -1.3982234 | SAR1A |
| 232164_s_at | -1.3983278 | EPPK1 |
| 209894_at | -1.3983574 | LEPR |
| 1554948_at | -1.398619 |  |
| 216201_at | -1.398619 |  |
| 201468_s_at | -1.3987087 | NQO1 |
| 236492_at | -1.3987827 | PPP2R2A |
| 232441_at | -1.3989834 | KRR1 |
| 241080_at | -1.3991584 |  |
| 214196_s_at | -1.399412 | TPP1 |
| 1554697_at | -1.3994292 | ADAMTS9 |
| 237908_at | -1.3994294 |  |
| 243236_at | -1.3994294 |  |
| 1558710_at | -1.3994299 |  |
| 209120_at | -1.3995328 | NR2F2 |
| 236223_s_at | -1.3998471 | RIT1 |
| 227203_at | -1.4001356 | FBXL17 |
| 227312_at | -1.4001408 | SNTB2 |
| 231055_at | -1.400174 |  |
| 1558830_at | -1.4004867 | OTTHUMG00000022014///RP3-341D10.4 |
| 239624_at | -1.400649 |  |
| 228723_at | -1.4009541 | NPTN-IT1 |
| 219757_s_at | -1.4013083 | TMEM260 |
| 243763_x_at | -1.4013547 |  |
| 214695_at | -1.4016511 | UBAP2L |
| 211049_at | -1.4018105 | TLX2 |
| 1552405_at | -1.4018373 | NLRP5 |
| 1559658_at | -1.4018373 | KATNBL1 |
| 207235_s_at | -1.4018373 | GRM5 |
| 215648_at | -1.4018373 |  |
| 230002_at | -1.4018373 | GPSM2 |
| 219587_at | -1.4020866 | TTC12 |
| 209007_s_at | -1.4029229 | C1orf63 |
| 233969_at | -1.403007 | IGLV9-49///IGLV9-49 |
| 220105_at | -1.4032336 | RTDR1 |
| 222496_s_at | -1.4032336 | RBM47 |
| 236597_at | -1.4032336 | UGT3A1 |
| 225732_at | -1.4033328 | KLHL42 |
| 1566903_at | -1.4034284 |  |
| 204832_s_at | -1.4035555 | BMPR1A |
| 1553500_at | -1.4035695 | FBXL21 |
| 229548_at | -1.4035695 |  |
| 218676_s_at | -1.4036354 | PCTP |
| 215213_at | -1.4039559 | NUP54 |
| 212781_at | -1.4042757 | RBBP6 |
| 215083_at | -1.4044623 |  |
| 239534_at | -1.4044623 |  |
| 1566032_at | -1.4044626 |  |
| 204159_at | -1.40457 | CDKN2C |
| 232637_at | -1.40457 | 2-Sep |
| 243249_at | -1.40457 |  |
| 1559746_a_at | -1.4048098 | ZNF718 |
| 1566984_at | -1.4049814 | LRRC37A16P///LRRC37A16P |
| 232879_at | -1.4050863 | CRTC3 |
| 241008_at | -1.4050872 |  |
| 231012_at | -1.4052902 | SLC35G1 |
| 214778_at | -1.4052907 | MEGF8 |
| 212847_at | -1.4053288 |  |
| 224784_at | -1.405338 | MLLT6 |
| 240410_at | -1.4054314 |  |
| 218953_s_at | -1.405891 | PCYOX1L |
| 235766_x_at | -1.4059049 | RAB27A |
| 244826_at | -1.4059155 |  |
| 226073_at | -1.405916 | TMEM218 |
| 207590_s_at | -1.4060034 | CENPI |
| 229211_at | -1.4064775 | DUSP28 |
| 229384_at | -1.4064775 | CTC-429P9.3///OTTHUMG00000182633 |
| 229607_at | -1.4066522 | AC005306.3///OTTHUMG00000180018 |
| 227627_at | -1.4067322 | C8orf44-SGK3///SGK3 |
| 240856_at | -1.4072586 | FFAR4 |
| 219649_at | -1.4074317 | ALG6 |
| 1565838_at | -1.4075115 |  |
| 225144_at | -1.4075321 | BMPR2 |
| 242487_at | -1.4077797 | CC2D1B |
| 212792_at | -1.4078411 | DPY19L1 |
| 1557623_at | -1.4082935 |  |
| 243565_at | -1.4083084 | CCDC150 |
| 239960_x_at | -1.4088507 | LYRM7 |
| 239635_at | -1.4089048 | RBM14 |
| 211721_s_at | -1.4093924 | ZNF551 |
| 228676_at | -1.4096235 | ORAOV1 |
| 203896_s_at | -1.4097469 | PLCB4 |
| 1553801_a_at | -1.4100344 | DTD2 |
| 1565830_at | -1.4100969 | OTTHUMG00000167487///RP11-178H8.7 |
| 237768_x_at | -1.4101589 |  |
| 217252_at | -1.4102064 |  |
| 242190_at | -1.4102069 |  |
| 222128_at | -1.4104007 | NSUN6 |
| 205395_s_at | -1.4105052 | MRE11A |
| 230165_at | -1.4106096 | SGOL2 |
| 215529_x_at | -1.410757 | DIP2A |
| 222344_at | -1.4107682 |  |
| 207601_at | -1.4109792 | SULT1B1 |
| 206295_at | -1.4110125 | IL18 |
| 228391_at | -1.4110209 | CYP4V2 |
| 214081_at | -1.4111325 | PLXDC1 |
| 232165_at | -1.411402 | EPPK1 |
| 221402_at | -1.4115779 | OR1F1 |
| 215281_x_at | -1.4117062 | POGZ |
| 1558628_at | -1.4118884 | RBFADN |
| 204076_at | -1.4120538 | ENTPD4 |
| 219760_at | -1.4126052 | LIN7B |
| 224327_s_at | -1.4133188 | DGAT2 |
| 209497_s_at | -1.4134322 | RBM4B |
| 1566139_at | -1.4139766 | HOPX |
| 1561886_a_at | -1.4140733 |  |
| 233417_at | -1.4140733 |  |
| 215743_at | -1.4143484 | NMT2 |
| 204284_at | -1.4143486 | PPP1R3C |
| 223385_at | -1.4143491 | CYP2S1 |
| 1556779_s_at | -1.4144794 | CTD-2547L24.4///OTTHUMG00000172988 |
| 208934_s_at | -1.4144794 | LGALS8 |
| 1561054_a_at | -1.4149238 | CCDC14 |
| 230404_at | -1.4154422 | C5orf63///LOC101060408 |
| 229774_at | -1.4154425 | CXXC4 |
| 244037_at | -1.4154425 | LOC100507520 |
| 232665_x_at | -1.415493 |  |
| 225185_at | -1.4156877 | MRAS |
| 1564773_x_at | -1.4161285 |  |
| 238666_at | -1.4162867 |  |
| 221222_s_at | -1.4163185 | C1orf56 |
| 227379_at | -1.4163349 | MBOAT1 |
| 238880_at | -1.4166734 | GTF3A |
| 243594_x_at | -1.4166734 | SPIRE2 |
| 212890_at | -1.4179611 | SLC38A10 |
| 242549_at | -1.4179945 | PRKD3 |
| 1569387_at | -1.4182391 | CSGALNACT1 |
| 225334_at | -1.4185598 | C10orf32 |
| 214433_s_at | -1.418586 | SELENBP1 |
| 229105_at | -1.4189556 | GPR39 |
| 235482_at | -1.419327 | PCBP1-AS1 |
| 1563715_at | -1.4196593 |  |
| 1561192_at | -1.4199328 |  |
| 219255_x_at | -1.4199338 | IL17RB |
| 241938_at | -1.4202052 | QKI |
| 236921_at | -1.4203416 |  |
| 204317_at | -1.420397 | TRMU |
| 223697_x_at | -1.4207971 | C9orf64 |
| 221684_s_at | -1.4214331 | NYX |
| 218212_s_at | -1.4216238 | MOCS2 |
| 238300_s_at | -1.4222606 | DAZAP2 |
| 223506_at | -1.4224623 | ZC3H8 |
| 233271_at | -1.4229015 |  |
| 241816_at | -1.423122 |  |
| 217164_at | -1.4231225 |  |
| 218892_at | -1.4231225 | DCHS1 |
| 241775_at | -1.4231225 |  |
| 230273_at | -1.423124 | C6orf165 |
| 1561345_at | -1.42319 | OTTHUMG00000013934///RP1-140J1.1 |
| 236314_at | -1.4232919 | EPM2AIP1 |
| 222294_s_at | -1.4233577 | RAB27A |
| 211350_s_at | -1.4233611 | KIF25-AS1 |
| 1561654_at | -1.4233613 |  |
| 237640_at | -1.4233615 | METTL21D |
| 1553905_at | -1.4233618 | CXorf22 |
| 213569_at | -1.423546 | LOC100506603 |
| 201681_s_at | -1.4238377 | DLG5 |
| 220576_at | -1.4239229 | PGAP1 |
| 202094_at | -1.423928 | BIRC5 |
| 225420_at | -1.424041 | GPAM |
| 217837_s_at | -1.424186 | CHMP3///RNF103-CHMP3 |
| 215339_at | -1.4246869 | NKTR |
| 223941_at | -1.4248301 | FBXO24 |
| 240467_at | -1.4248301 |  |
| 220232_at | -1.4248697 | SCD5 |
| 1566887_x_at | -1.4249068 |  |
| 214804_at | -1.4250929 | CENPI |
| 229334_at | -1.4251772 | RUFY3 |
| 212783_at | -1.4252728 | RBBP6 |
| 229562_at | -1.4260163 | RPL10A |
| 207930_at | -1.4260966 | LCN1 |
| 202534_x_at | -1.426238 | DHFR |
| 233897_at | -1.4265066 | FEZF2 |
| 242829_x_at | -1.4267886 |  |
| 208581_x_at | -1.4271998 | MT1X |
| 1553181_at | -1.4274169 | DDX31 |
| 205739_x_at | -1.4275216 | ZNF107 |
| 227578_at | -1.4278793 | TMPO-AS1 |
| 240013_at | -1.4280379 |  |
| 243718_at | -1.4285157 |  |
| 225313_at | -1.4288292 | FAM217B |
| 220173_at | -1.4294125 | CCDC176 |
| 224702_at | -1.4294168 | TMEM167A |
| 231618_s_at | -1.429859 | SUN3 |
| 207598_x_at | -1.42993 | XRCC2 |
| 203485_at | -1.4300538 | RTN1 |
| 236291_at | -1.4305348 | RDH5 |
| 241223_x_at | -1.4306792 |  |
| 202182_at | -1.4310467 | KAT2A |
| 205452_at | -1.4311734 | PIGB |
| 229145_at | -1.4318686 | ANAPC16 |
| 235803_at | -1.4321061 |  |
| 231979_at | -1.4324162 |  |
| 1564474_at | -1.4327401 | ZBED3-AS1 |
| 1567273_at | -1.4329548 | OR2K2 |
| 240208_at | -1.4333447 |  |
| 227501_at | -1.4335917 |  |
| 208937_s_at | -1.4336212 | ID1 |
| 232931_at | -1.4338026 | SNRNP200 |
| 1553588_at | -1.4338695 | ND3///SH3KBP1 |
| 204531_s_at | -1.434206 | BRCA1 |
| 222400_s_at | -1.4343159 | ADI1 |
| 1554670_at | -1.434325 | GGA1 |
| 225840_at | -1.4344236 | TEF |
| 221120_at | -1.4344677 | FLJ20306 |
| 219147_s_at | -1.4346948 | NMRK1 |
| 226877_at | -1.4347569 | RPL32P3 |
| 229470_at | -1.4347901 |  |
| 216944_s_at | -1.435783 | ITPR1 |
| 218553_s_at | -1.4358077 | KCTD15 |
| 207828_s_at | -1.4360691 | CENPF |
| 1570230_at | -1.4362286 | AC007099.1///OTTHUMG00000152941 |
| 1566191_at | -1.4363958 | SUZ12 |
| 214417_s_at | -1.4367719 | FETUB |
| 237504_at | -1.4367719 | INTS10 |
| 237213_at | -1.4368433 |  |
| 206186_at | -1.4370008 | MPP3 |
| 234276_at | -1.437047 |  |
| 229295_at | -1.4371052 | IL17RA |
| 226485_at | -1.4373232 | VSIG10 |
| 1561134_at | -1.4375098 |  |
| 227802_at | -1.4375888 | RUFY3 |
| 202379_s_at | -1.4376934 | NKTR |
| 205756_s_at | -1.4383817 | F8 |
| 216918_s_at | -1.4384387 | DST |
| 242886_at | -1.4386165 |  |
| 239841_at | -1.4396935 |  |
| 225176_at | -1.4399875 | LNPEP |
| 205355_at | -1.4402541 | ACADSB |
| 1567224_at | -1.4402579 | HMGA2 |
| 1553990_at | -1.4402589 | BRICD5 |
| 214059_at | -1.4402589 | IFI44 |
| 222145_at | -1.4402589 |  |
| 235053_at | -1.4402589 | OTTHUMG00000177839///RP11-257O5.2 |
| 236244_at | -1.4402589 |  |
| 233461_x_at | -1.4404817 | ZNF226 |
| 243690_at | -1.4411874 | TRIOBP |
| 239251_at | -1.4413084 |  |
| 240908_at | -1.4413084 |  |
| 1552279_a_at | -1.4413936 | SLC46A1 |
| 222745_s_at | -1.4417706 | KATNBL1 |
| 237062_at | -1.4418156 |  |
| 227314_at | -1.4425389 | ITGA2 |
| 208219_at | -1.4430492 | ACVR1B |
| 242389_at | -1.4432938 | LUC7L3 |
| 242853_at | -1.4435701 |  |
| 219325_s_at | -1.4439509 | ELAC1 |
| 200768_s_at | -1.4443738 | MAT2A |
| 218095_s_at | -1.4444606 | TMEM165 |
| 236201_at | -1.4444903 |  |
| 207394_at | -1.4444906 | ZNF137P |
| 243561_at | -1.4446459 |  |
| 1558703_at | -1.4452735 | SLC46A1 |
| 218639_s_at | -1.445715 | ZXDC |
| 1552729_at | -1.4457436 | SNHG7///SNORA17///SNORA43 |
| 242881_x_at | -1.4458325 | LOC100506303///LOC100653149///LOC101060483///LOC400879///LOC440157 |
| 225532_at | -1.4462053 | CABLES1 |
| 1558942_at | -1.4463893 | ZNF765 |
| 239909_at | -1.4465375 | ADAMTSL1 |
| 1557193_at | -1.4465377 |  |
| 232622_at | -1.4468613 |  |
| 237184_at | -1.4468622 |  |
| 241694_at | -1.4473075 | PKHD1 |
| 244804_at | -1.4475788 | SQSTM1 |
| 200661_at | -1.4476418 | CTSA |
| 225917_at | -1.44782 |  |
| 207218_at | -1.4479307 | F9 |
| 212253_x_at | -1.4479309 | DST |
| 232091_s_at | -1.4479309 | ZDHHC24 |
| 239619_at | -1.4479309 |  |
| 240128_at | -1.4479309 |  |
| 244776_at | -1.4479309 |  |
| 1559691_at | -1.4479314 |  |
| 232278_s_at | -1.4479806 | DEPDC1 |
| 227687_at | -1.4481167 | HYLS1 |
| 207034_s_at | -1.4482381 | GLI2 |
| 215129_at | -1.4482381 | PIK3C2G |
| 1557292_a_at | -1.4486624 | MCOLN3 |
| 207038_at | -1.4489219 | SLC16A6 |
| 210598_at | -1.4490435 |  |
| 235159_at | -1.4493808 |  |
| 212400_at | -1.4496812 | FAM102A |
| 1559780_at | -1.4496847 |  |
| 226885_at | -1.4496931 | RNF217 |
| 231829_at | -1.4502913 | MAVS |
| 1556486_at | -1.4507651 | OTTHUMG00000176169///RP11-292F22.7 |
| 1569380_a_at | -1.4507678 | HERPUD1 |
| 244460_at | -1.4512646 | TMEM225 |
| 231761_at | -1.4513443 | FFAR1 |
| 211552_s_at | -1.451932 | ALDH4A1 |
| 1554094_at | -1.4519324 | ENTPD5 |
| 231690_at | -1.4519324 |  |
| 222664_at | -1.45211 | KCTD15 |
| 214727_at | -1.45216 | BRCA2 |
| 225531_at | -1.453077 | CABLES1 |
| 237475_x_at | -1.453308 | CCDC152 |
| 233922_at | -1.4534853 |  |
| 1557759_at | -1.4539772 | ATP5SL |
| 238522_at | -1.4539772 | OTTHUMG00000018470///RP11-137L10.6 |
| 230312_at | -1.4540446 | LOC100507312 |
| 228582_x_at | -1.4541253 | MALAT1 |
| 242932_at | -1.4543945 |  |
| 1557828_a_at | -1.4544281 | C5orf28 |
| 242981_at | -1.4545479 |  |
| 212543_at | -1.4549373 | AIM1 |
| 237794_at | -1.4549373 | ANKRD30BL///ANKRD30BP2///LOC100132154 |
| 202091_at | -1.4549376 | OTTHUMG00000175518///RP11-407G23.3 |
| 225217_s_at | -1.4551556 | BRPF3 |
| 227037_at | -1.4553081 | PLD6 |
| 231863_at | -1.4553384 | ING3 |
| 206487_at | -1.4554889 | SUN1 |
| 1558331_at | -1.4556333 | SIRT2 |
| 217966_s_at | -1.4556333 | FAM129A |
| 1557360_at | -1.4556545 | LRPPRC |
| 230235_at | -1.4559615 | MCTS1 |
| 1555980_a_at | -1.4560928 | OTTHUMG00000040718///RP11-54O7.3 |
| 1555960_at | -1.4561241 | HINT1 |
| 203549_s_at | -1.456359 | LPL |
| 219392_x_at | -1.4564649 | PRR11 |
| 227139_s_at | -1.4565997 | HPS3 |
| 225784_s_at | -1.4572821 | ZC4H2 |
| 1557371_a_at | -1.4573687 | LINC00961 |
| 240984_at | -1.4573687 |  |
| 219332_at | -1.4575932 | MICALL2 |
| 238040_at | -1.4576402 |  |
| 222437_s_at | -1.4578481 | CHMP3///RNF103-CHMP3 |
| 224436_s_at | -1.4584881 | NIPSNAP3A |
| 229574_at | -1.4584963 | TRA2A |
| 232518_at | -1.4585258 | HELZ2 |
| 215029_at | -1.4589963 |  |
| 1555834_at | -1.4591198 | UCHL1 |
| 227770_at | -1.4591361 | COG8 |
| 226393_at | -1.4592205 | CYP2U1 |
| 1553269_at | -1.4596995 | ZNF718 |
| 241844_x_at | -1.4597082 | TMEM156 |
| 233063_s_at | -1.4604659 | OTTHUMG00000014120///RP1-80B9.2 |
| 226675_s_at | -1.4607913 | MALAT1 |
| 1552287_s_at | -1.4608077 | AFG3L1P |
| 230904_at | -1.4614046 | FSD1L |
| 243591_at | -1.461408 |  |
| 217643_x_at | -1.4617089 |  |
| 244756_at | -1.4618567 | ANKRD13D |
| 227505_at | -1.4619244 |  |
| 213326_at | -1.4620216 | VAMP1 |
| 227663_at | -1.4624256 |  |
| 1563063_at | -1.4624261 |  |
| 219118_at | -1.4624261 | FKBP11 |
| 226085_at | -1.4625218 | CBX5 |
| 201109_s_at | -1.4629182 | THBS1 |
| 231223_at | -1.4629424 | CSMD1 |
| 206210_s_at | -1.4631655 | CETP |
| 215338_s_at | -1.4631808 | NKTR |
| 232398_at | -1.4634434 | CCDC150 |
| 239620_at | -1.4637445 | TTC23L |
| 215882_at | -1.4639649 | CEP152 |
| 218641_at | -1.4646618 | C11orf95 |
| 213385_at | -1.4648502 | CHN2 |
| 218424_s_at | -1.4651285 | STEAP3 |
| 216081_at | -1.4655015 | LAMA4 |
| 239730_at | -1.4657971 | DGCR14///TSSK2 |
| 219405_at | -1.4662491 | TRIM68 |
| 1557419_a_at | -1.4664919 | ACSL4 |
| 242184_s_at | -1.4666986 | AC025442.3///OTTHUMG00000164088 |
| 225549_at | -1.4667488 | DDX6 |
| 1570143_at | -1.46676 |  |
| 238179_at | -1.467548 |  |
| 230063_at | -1.4675757 | ZNF264 |
| 227754_at | -1.4677862 |  |
| 1569723_a_at | -1.4681389 | SPIRE2 |
| 230067_at | -1.468966 | FAM124A |
| 231136_at | -1.468966 |  |
| 215843_s_at | -1.4692597 | TLL2 |
| 228847_at | -1.4693618 | EXOC3 |
| 242472_x_at | -1.4699188 | FNBP4 |
| 1556054_at | -1.4700423 |  |
| 242191_at | -1.4704864 | NBPF10 |
| 226848_at | -1.4707718 |  |
| 1559391_s_at | -1.4709094 |  |
| 243201_at | -1.4715925 | RPL36A |
| 242836_at | -1.471802 |  |
| 240140_s_at | -1.4718022 | LRIG1 |
| 1562013_a_at | -1.4719462 | LOC100506730 |
| 1558556_at | -1.4721487 | CAMK1 |
| 230406_at | -1.4721487 |  |
| 244515_at | -1.4726192 |  |
| 226019_at | -1.4727589 | OMA1 |
| 225473_at | -1.4729809 | SOGA1 |
| 225107_at | -1.4730918 | HNRNPA2B1 |
| 242364_x_at | -1.473142 | TNRC6C-AS1 |
| 225321_s_at | -1.4734113 | PILRB |
| 233249_at | -1.473498 | LOC100507073 |
| 238988_at | -1.4738594 |  |
| 217169_at | -1.474493 | IGHA1///IGHG1///IGHM |
| 1554001_at | -1.4745299 | TRIM37 |
| 219288_at | -1.4745474 | C3orf14 |
| 1558154_at | -1.4745635 |  |
| 206554_x_at | -1.4745938 | SETMAR |
| 205097_at | -1.4747555 | SLC26A2 |
| 207944_at | -1.4748698 | OCM2 |
| 1558346_at | -1.475781 | COX17 |
| 230384_at | -1.4757819 | ANKRD23 |
| 230435_at | -1.4757819 | FAM228B |
| 224893_at | -1.4761918 | ATL3 |
| 235521_at | -1.4761995 | HOXA3 |
| 242368_at | -1.4762825 |  |
| 225851_at | -1.4764142 | CHURC1-FNTB///FNTB |
| 227805_at | -1.4764674 | METAP1D |
| 220387_s_at | -1.4765799 | HHLA3 |
| 229015_at | -1.4766992 | LOC286367 |
| 232569_at | -1.476726 |  |
| 220227_at | -1.4768249 | CDH4 |
| 235317_at | -1.4779034 | LOC284454 |
| 236852_at | -1.4779434 | FBXO43 |
| 216115_at | -1.4781027 | NF1 |
| 236274_at | -1.4792472 | EIF3B |
| 1556654_at | -1.4796274 | CDK12 |
| 235621_at | -1.479771 | FAHD2A |
| 243869_at | -1.4800473 |  |
| 230341_x_at | -1.4800855 | ADAMTS10 |
| 226365_at | -1.4813275 | OTTHUMG00000176823///RP11-846E15.2 |
| 239413_at | -1.4815979 | CEP152 |
| 221485_at | -1.4816644 | B4GALT5 |
| 227401_at | -1.4820739 | IL17D |
| 241672_at | -1.4820739 | SERTM1 |
| 1557293_at | -1.482556 | LINC00969 |
| 1566480_x_at | -1.4829088 | OTTHUMG00000181824///RP11-1072C15.6 |
| 222380_s_at | -1.4831108 | PDCD6 |
| 202380_s_at | -1.4831295 | NKTR |
| 205914_s_at | -1.4831716 | GRIN1 |
| 221384_at | -1.4840769 | UCP1 |
| 235985_at | -1.4840769 |  |
| 226568_at | -1.4841543 | FAM102B |
| 228714_at | -1.4844742 |  |
| 215889_at | -1.4853194 | SKIL |
| 206746_at | -1.4854882 | BFSP1 |
| 211879_x_at | -1.4856576 | PCDHGA3 |
| 1559139_at | -1.4864681 | NOC2L |
| 235590_at | -1.4867152 | FAM178A |
| 243532_at | -1.4868518 | LNPEP |
| 230868_at | -1.4868786 |  |
| 1568615_a_at | -1.4869907 | SRD5A3-AS1 |
| 220033_at | -1.4873463 |  |
| 205051_s_at | -1.487537 | KIT |
| 228393_s_at | -1.488258 | ZNF302 |
| 221242_at | -1.4883307 |  |
| 232909_s_at | -1.488438 | BPTF |
| 221045_s_at | -1.488873 | PER3 |
| 238761_at | -1.4891092 | ELK4 |
| 214202_at | -1.4898777 | CTC-428G20.3///OTTHUMG00000162912 |
| 1569114_at | -1.4899924 | AC099850.1///OTTHUMG00000132079 |
| 212361_s_at | -1.4902012 | ATP2A2 |
| 214755_at | -1.4902033 | UAP1L1 |
| 228180_at | -1.4903244 |  |
| 222351_at | -1.4909146 | PPP2R1B |
| 213649_at | -1.4910635 | SRSF7 |
| 232169_x_at | -1.4913177 | MIR4691///NDUFS8 |
| 229531_at | -1.4915307 |  |
| 1552698_at | -1.491757 | TUBA3FP |
| 242121_at | -1.4919552 |  |
| 240168_at | -1.492573 | XPO7 |
| 215932_at | -1.4925733 | MAGEC2 |
| 229926_at | -1.4929675 | MIR3682 |
| 219151_s_at | -1.4930834 | RABL2A///RABL2B |
| 229126_at | -1.4934565 | TMEM19 |
| 221815_at | -1.4938357 | ABHD2 |
| 219544_at | -1.4946812 | BORA |
| 219185_at | -1.495423 | SIRT5 |
| 227792_at | -1.4954442 | ITPRIPL2 |
| 232458_at | -1.4957862 | COL3A1 |
| 1557215_at | -1.4961232 | LINC00648 |
| 220527_at | -1.4966645 | MRPL20 |
| 224096_at | -1.4966645 |  |
| 1558621_at | -1.497777 | CABLES1 |
| 242287_at | -1.4981334 | CLIP1 |
| 241341_at | -1.498586 |  |
| 232362_at | -1.498596 | CCDC18 |
| 236019_at | -1.498862 | RAB12 |
| 226490_at | -1.4989349 | NHSL1 |
| 224664_at | -1.4989953 | ANAPC16 |
| 238651_at | -1.499232 |  |
| 223324_s_at | -1.4994526 | TRPM7 |
| 230521_at | -1.4995086 | ARHGEF39 |
| 222750_s_at | -1.4995939 | SRD5A3 |
| 238002_at | -1.500401 | GOLIM4 |
| 214594_x_at | -1.500452 | ATP8B1 |
| 230885_at | -1.5010135 | SPG7 |
| 209729_at | -1.5012875 | GAS2L1 |
| 235658_at | -1.5014515 |  |
| 215114_at | -1.5014685 | SENP3 |
| 235008_at | -1.5016376 |  |
| 223494_at | -1.5019406 | MGEA5 |
| 242460_at | -1.5020181 |  |
| 237172_at | -1.50352 |  |
| 241692_at | -1.5035205 |  |
| 1555117_at | -1.5035851 |  |
| 236619_at | -1.5035851 |  |
| 218192_at | -1.5036577 | IP6K2 |
| 237318_at | -1.5037233 |  |
| 207963_at | -1.5038346 | KIF25-AS1 |
| 237251_at | -1.5042682 | LRRC71 |
| 206601_s_at | -1.5043 | HOXD3 |
| 211005_at | -1.5045451 | LAT |
| 1555847_a_at | -1.5047141 | LOC284454 |
| 229468_at | -1.5050275 | CDK3///TEN1-CDK3 |
| 1556818_at | -1.5060698 |  |
| 242378_at | -1.50607 | MYADML2 |
| 220572_at | -1.5063518 | LOC100506282 |
| 236907_at | -1.5063523 |  |
| 239771_at | -1.50677 | CAND1 |
| 236433_at | -1.5068866 | LOC100506713 |
| 244185_at | -1.5072607 |  |
| 235926_at | -1.5072742 | ANAPC5 |
| 215567_at | -1.5083067 | FCF1 |
| 225488_at | -1.5086563 | DERL1 |
| 230152_at | -1.5086572 | WDR52 |
| 240170_at | -1.508922 |  |
| 242759_at | -1.5100331 |  |
| 203744_at | -1.5108274 | HMGB3 |
| 216175_at | -1.5108423 |  |
| 228523_at | -1.5109746 | NANOS1 |
| 221213_s_at | -1.5118774 | ZNF280D |
| 229455_at | -1.5118784 | AC083843.1///OTTHUMG00000164324 |
| 213758_at | -1.5121198 | COX4I1 |
| 235788_at | -1.5129461 | HCG18 |
| 212289_at | -1.5132742 | ANKRD12 |
| 213578_at | -1.5136003 | BMPR1A |
| 1552621_at | -1.5139235 | POLR2J2///POLR2J3///UPK3BL |
| 232589_at | -1.5140316 | LOC100506029///LOC100506051 |
| 220702_at | -1.5143797 |  |
| 242578_x_at | -1.5145286 | SLC22A3 |
| 231950_at | -1.5145302 | LOC653501///ZNF658///ZNF658B |
| 220144_s_at | -1.5148104 | ANKEF1 |
| 208707_at | -1.5148705 | EIF5 |
| 203767_s_at | -1.5149637 | STS |
| 227514_at | -1.5154439 | ITPRIPL2 |
| 220654_at | -1.5155106 | PPY2 |
| 204995_at | -1.5155292 | CDK5R1 |
| 1556338_at | -1.5155767 |  |
| 1558444_at | -1.5160286 |  |
| 241425_at | -1.5171574 | NUPL1 |
| 221103_s_at | -1.5172538 | WDR52 |
| 244018_at | -1.5177242 |  |
| 1557071_s_at | -1.517725 | NUB1 |
| 206696_at | -1.5180603 | GPR143 |
| 213650_at | -1.5188522 | GOLGA8A///GOLGA8B |
| 228392_at | -1.5201898 | ZNF302 |
| 239793_at | -1.5207154 | OTTHUMG00000183943///RP11-73M18.6 |
| 239866_at | -1.5210296 |  |
| 243431_at | -1.5210296 |  |
| 230589_at | -1.5211811 | TRAF3IP2-AS1 |
| 1562674_at | -1.5221648 | OTTHUMG00000014089///RP11-495P10.5 |
| 242439_s_at | -1.5233302 | ASXL1 |
| 1563601_at | -1.5240703 |  |
| 202127_at | -1.524388 | PRPF4B |
| 1559054_a_at | -1.5250816 |  |
| 229193_at | -1.5250819 | LUC7L3 |
| 241702_at | -1.5250819 |  |
| 237347_at | -1.5251615 |  |
| 215409_at | -1.5255104 | LPCAT4 |
| 1560297_at | -1.525763 |  |
| 217152_at | -1.525817 |  |
| 236961_at | -1.5260863 |  |
| 210909_x_at | -1.5265507 | LPAL2 |
| 206262_at | -1.526551 | ADH1C |
| 225424_at | -1.5267947 | GPAM |
| 225594_at | -1.5268613 | CREBZF |
| 216263_s_at | -1.5276147 | NGDN |
| 243441_at | -1.527671 | HOXB7 |
| 232889_at | -1.5279734 |  |
| 203106_s_at | -1.5286771 | VPS41 |
| 232478_at | -1.5287923 | MIR181A2HG |
| 229551_x_at | -1.52895 | ZNF367 |
| 217497_at | -1.5307592 | TYMP |
| 228047_at | -1.5315071 | SNORA72 |
| 1565651_at | -1.5318569 |  |
| 217657_at | -1.5330082 |  |
| 233768_at | -1.5330994 |  |
| 209360_s_at | -1.5331095 | LOC100506403///RUNX1 |
| 206764_x_at | -1.5338293 | MPPE1 |
| 221860_at | -1.5340625 | HNRNPL |
| 211200_s_at | -1.5344357 | EFCAB2 |
| 213426_s_at | -1.5345037 | CAV2 |
| 242471_at | -1.5357392 |  |
| 1558922_at | -1.5364128 |  |
| 213430_at | -1.537124 | RUFY3 |
| 225767_at | -1.5374644 | RNA45S5 |
| 201502_s_at | -1.5380408 | NFKBIA |
| 223115_at | -1.5388544 | MED17 |
| 221989_at | -1.5395137 | RPL10///SNORA70 |
| 226164_x_at | -1.5405556 | RIMKLB |
| 244753_at | -1.5409716 |  |
| 236985_at | -1.5411686 |  |
| 1570130_at | -1.5425987 | SPATS2 |
| 236505_at | -1.5431447 | NUP62 |
| 229170_s_at | -1.5432403 | TTC18 |
| 1558275_at | -1.5445768 | OTTHUMG00000177665///RP11-295D4.3 |
| 239179_at | -1.5445778 |  |
| 206675_s_at | -1.5449474 | SKIL |
| 243664_at | -1.5465037 | TXNL1 |
| 238714_at | -1.5466375 |  |
| 235290_at | -1.5478485 | ZNF782 |
| 242751_at | -1.5487303 |  |
| 220370_s_at | -1.5492524 | USP36 |
| 1556837_a_at | -1.549383 |  |
| 236007_at | -1.5498425 | AKAP10 |
| 243537_at | -1.5498735 |  |
| 224156_x_at | -1.5498962 | IL17RB |
| 210034_s_at | -1.5507653 | RPL5///SNORD21 |
| 214917_at | -1.5512236 | PRKAA1 |
| 229689_s_at | -1.5519477 | OTTHUMG00000018545///RP13-39P12.3 |
| 1555533_at | -1.5521783 | QRFPR |
| 231332_at | -1.552512 |  |
| 235513_at | -1.5527055 |  |
| 213593_s_at | -1.5536464 | TRA2A |
| 1560652_at | -1.5567064 | OTTHUMG00000175497///RP6-24A23.7 |
| 204279_at | -1.5567733 | PSMB9 |
| 243444_at | -1.5568278 | SRD5A3 |
| 229656_s_at | -1.5568706 | EML6 |
| 225731_at | -1.5570167 | ANKRD50 |
| 232282_at | -1.5579963 | WNK3 |
| 1555982_at | -1.5584954 | ZFYVE16 |
| 232427_at | -1.5592927 | ZNF224 |
| 225579_at | -1.5612816 | PQLC3 |
| 217670_at | -1.5616634 | RPLP2 |
| 230187_s_at | -1.5622324 | OTTHUMG00000175906///RP11-457M11.5 |
| 218800_at | -1.5622603 | SRD5A3 |
| 226077_at | -1.5623015 | RNF145 |
| 212730_at | -1.5628666 | SYNM |
| 1556821_x_at | -1.5636127 | DLEU2 |
| 209604_s_at | -1.5637336 | GATA3 |
| 220277_at | -1.5639998 | CXXC4 |
| 230270_at | -1.5648426 | PRPF38B |
| 220843_s_at | -1.564987 | DCAF13 |
| 220720_x_at | -1.5651146 | MZT2B |
| 227223_at | -1.5654199 | RBM39 |
| 241798_at | -1.5662013 |  |
| 220590_at | -1.5665659 | ITFG2 |
| 229765_at | -1.5674583 | ZNF207 |
| 229415_at | -1.5675298 | CYCS |
| 242146_at | -1.5681982 | SNRPA1 |
| 233518_at | -1.5700222 |  |
| 228813_at | -1.5712593 | HDAC4 |
| 240998_at | -1.5734745 |  |
| 1556060_a_at | -1.5735928 | ZNF451 |
| 1553570_x_at | -1.574498 | COX2///OAF///TLE1 |
| 221024_s_at | -1.5752059 | SLC2A10 |
| 238299_at | -1.5762122 |  |
| 215599_at | -1.5762129 | GUSBP3///GUSBP9///LOC100653061///LOC101060519 |
| 206406_at | -1.5762131 | SMCP |
| 229467_at | -1.5766968 | PCBP2 |
| 210764_s_at | -1.5770147 | CYR61 |
| 209304_x_at | -1.5771397 | GADD45B |
| 229962_at | -1.5783079 | LRRC37A3 |
| 236330_at | -1.578565 |  |
| 201818_at | -1.578671 | LPCAT1 |
| 244778_x_at | -1.578793 |  |
| 230337_at | -1.5796454 | SOS1 |
| 1557543_at | -1.5807124 |  |
| 221963_x_at | -1.5811111 | ZNF587B |
| 227260_at | -1.5811968 |  |
| 1554287_at | -1.5814105 | TRIM4 |
| 222958_s_at | -1.5834284 | DEPDC1 |
| 241786_at | -1.5834593 |  |
| 1556049_at | -1.5846754 | RTN4 |
| 224959_at | -1.584775 | SLC26A2 |
| 237118_at | -1.5849518 |  |
| 223553_s_at | -1.5849521 | DOK3 |
| 239188_at | -1.5860198 | PPP2R3C |
| 239934_x_at | -1.5872052 |  |
| 239753_at | -1.5889494 | ZNF252P |
| 1555343_at | -1.5895561 | MEGF10 |
| 222662_at | -1.589574 | PPP1R3B |
| 207778_at | -1.5900921 | REG1P |
| 235927_at | -1.59045 | XPO1 |
| 233019_at | -1.5908451 | CNOT7 |
| 228841_at | -1.5911467 | LYRM7 |
| 224373_s_at | -1.5921915 | C10orf99///CCDC104///HNRNPM///ND4 |
| 219062_s_at | -1.592643 | ZCCHC2 |
| 232816_s_at | -1.593671 | DDX11 |
| 207306_at | -1.5950441 | TCF15 |
| 204894_s_at | -1.595117 | AOC3 |
| 236484_at | -1.5951174 |  |
| 232464_at | -1.5961994 | TRIM78P |
| 230355_at | -1.5974394 | SEPT7P2 |
| 220969_s_at | -1.5980514 |  |
| 210075_at | -1.5994407 | 2-Mar |
| 240482_at | -1.5997336 |  |
| 215545_at | -1.6013905 |  |
| 235919_at | -1.6016324 | CEP78 |
| 224559_at | -1.6039542 | MALAT1 |
| 207953_at | -1.6043525 |  |
| 214696_at | -1.6053916 | MIR22///MIR22HG |
| 228200_at | -1.6069475 | ZNF252P |
| 242645_at | -1.6071223 |  |
| 224963_at | -1.6079496 | SLC26A2 |
| 217761_at | -1.6083753 | ADI1 |
| 215011_at | -1.6096357 | SNHG3///SNORA73A |
| 232287_at | -1.6096597 | ERCC6-PGBD3///PGBD3 |
| 203068_at | -1.6102992 | KLHL21 |
| 218477_at | -1.6103221 | TMEM14A |
| 230424_at | -1.610978 | NREP |
| 242133_s_at | -1.6113667 |  |
| 221290_s_at | -1.611821 | MUM1 |
| 223608_at | -1.6120327 | EFCAB2 |
| 230748_at | -1.6120331 | SLC16A6 |
| 226318_at | -1.612169 | TBRG1 |
| 1553569_at | -1.6124631 | COX2///OAF///TLE1 |
| 223774_at | -1.6131157 | SNHG12///SNORA16A///SNORA44///SNORA61 |
| 228286_at | -1.6131653 | GEN1 |
| 218456_at | -1.6169654 | CAPRIN2 |
| 214705_at | -1.6180924 | INADL |
| 240052_at | -1.6180924 | ITPR1 |
| 240344_x_at | -1.6189398 | LYRM7 |
| 210172_at | -1.6198443 | SF1 |
| 214731_at | -1.6213859 |  |
| 225182_at | -1.6232176 | TMEM50B |
| 208278_s_at | -1.6248204 |  |
| 206546_at | -1.6250116 | SYCP2 |
| 228173_at | -1.6253955 | GNAS |
| 229966_at | -1.6274254 | EWSR1 |
| 202792_s_at | -1.6275814 | PPP6R2 |
| 223773_s_at | -1.6281996 | SNHG12///SNORA16A///SNORA44///SNORA61 |
| 230522_s_at | -1.6294949 | ARHGEF39 |
| 232424_at | -1.6294951 | PRDM16 |
| 218032_at | -1.6305455 | SNN |
| 219814_at | -1.6307458 | MBNL3 |
| 208741_at | -1.6307762 | SAP18 |
| 219848_s_at | -1.6312889 | ZNF432 |
| 242688_at | -1.6327825 |  |
| 241838_at | -1.6327922 |  |
| 205092_x_at | -1.6335015 | ZBTB1 |
| 229159_at | -1.633533 | THSD7A |
| 231598_x_at | -1.6339712 |  |
| 235273_at | -1.6381859 | DYX1C1 |
| 210002_at | -1.6391153 | GATA6 |
| 44783_s_at | -1.6392908 | HEY1 |
| 226020_s_at | -1.6393645 | DAB1///OMA1 |
| 215301_at | -1.6405299 | SYCE1L |
| 217967_s_at | -1.6407273 | FAM129A |
| 243318_at | -1.6416429 | DCAF8 |
| 231208_at | -1.6424022 |  |
| 228097_at | -1.6424028 | MYLIP |
| 240286_at | -1.642403 |  |
| 213761_at | -1.6425563 | MDM1 |
| 222585_x_at | -1.6426156 | KRCC1 |
| 228908_s_at | -1.6431656 | LOC642852 |
| 235511_at | -1.6442198 |  |
| 236976_at | -1.6442201 | FANCA |
| 217653_x_at | -1.6452094 |  |
| 240392_at | -1.645774 | OTTHUMG00000177568///RP11-334C17.5 |
| 214805_at | -1.6459491 | EIF4A1///SNORA48///SNORA67///SNORD10 |
| 230229_at | -1.6481473 | DLG1 |
| 239383_at | -1.6481594 |  |
| 244848_at | -1.6494488 | LOC100505794 |
| 241722_x_at | -1.6513022 |  |
| 228259_s_at | -1.6521817 | EPB41L4A-AS1 |
| 239944_at | -1.6521921 |  |
| 226663_at | -1.6529241 | ANKRD10-IT1 |
| 229511_at | -1.6529241 | SMARCE1 |
| 1555878_at | -1.6533016 | RPS24 |
| 1569958_at | -1.6534156 | OTTHUMG00000009207///RP4-694A7.4 |
| 236022_at | -1.6535645 | MYO19 |
| 232601_at | -1.654394 |  |
| 228745_at | -1.6549761 | SGTB |
| 238738_at | -1.657388 |  |
| 231229_at | -1.6591792 | HILS1 |
| 206497_at | -1.6598824 | COA1 |
| 202741_at | -1.66089 | PRKACB |
| 239797_at | -1.6620767 |  |
| 214982_at | -1.6623101 | SNRNP200 |
| 235656_s_at | -1.6630251 |  |
| 212097_at | -1.6647954 | CAV1 |
| 240570_at | -1.6658987 | INADL |
| 213653_at | -1.667911 | METTL3 |
| 232958_at | -1.6680053 |  |
| 232370_at | -1.6699514 | LOC254057 |
| 1567080_s_at | -1.6713264 | CLN6 |
| 204075_s_at | -1.6715457 | CEP104 |
| 203585_at | -1.6716833 | ZNF185 |
| 223080_at | -1.6722679 | GLS |
| 244139_s_at | -1.6726261 |  |
| 204202_at | -1.6747704 | IQCE |
| 219142_at | -1.6755587 | RASL11B |
| 228216_at | -1.6761215 |  |
| 228997_at | -1.6763763 | TRNAU1AP |
| 230388_s_at | -1.6786762 | KANSL1-AS1 |
| 211386_at | -1.6788019 | MGC12488 |
| 232417_x_at | -1.6792161 | ZDHHC11 |
| 232273_at | -1.6804271 |  |
| 226402_at | -1.6813415 | CYP2U1 |
| 207826_s_at | -1.6819518 | ID3 |
| 204085_s_at | -1.6873038 | CLN5 |
| 207147_at | -1.6888404 | DLX2 |
| 226444_at | -1.6899058 |  |
| 235581_at | -1.6910031 |  |
| 1560477_a_at | -1.6929172 | SAMD11 |
| 238982_at | -1.6933938 | DENR |
| 241617_x_at | -1.6935043 |  |
| 242260_at | -1.6961119 | MATR3 |
| 227074_at | -1.6973732 | LOC100131564 |
| 212660_at | -1.7007133 | PHF15 |
| 213517_at | -1.7012278 | PCBP2 |
| 215224_at | -1.7044386 | RPL23///SNORA21 |
| 237246_at | -1.7050415 |  |
| 244546_at | -1.7050872 | CYCS |
| 243463_s_at | -1.706702 | RIT1 |
| 229274_at | -1.707085 | GNAS |
| 238156_at | -1.7077695 |  |
| 230927_at | -1.7133571 |  |
| 209750_at | -1.7142992 | NR1D2 |
| 225227_at | -1.7143937 | SKIL |
| 219433_at | -1.7150557 | BCOR |
| 237577_at | -1.7151793 | PCNP |
| 242975_s_at | -1.7156862 |  |
| 214920_at | -1.7157356 | THSD7A |
| 213359_at | -1.7186244 | HNRNPD |
| 239469_at | -1.7193321 |  |
| 1554476_x_at | -1.7196511 | ZNF808 |
| 31874_at | -1.7209384 | GAS2L1 |
| 231880_at | -1.7232083 | STRIP2 |
| 242233_at | -1.7234088 |  |
| 239212_at | -1.724844 | LTV1 |
| 215008_at | -1.7255588 | TLL2 |
| 230387_at | -1.7271638 |  |
| 233011_at | -1.7300805 | ANXA1 |
| 234452_at | -1.7304523 | HIBADH |
| 222668_at | -1.7329592 | KCTD15 |
| 235469_at | -1.7341074 | FAM133B///FAM133DP |
| 1566482_at | -1.7350471 | OTTHUMG00000168867///RP11-305O6.3 |
| 207574_s_at | -1.7352289 | GADD45B |
| 213939_s_at | -1.7370726 | RUFY3 |
| 204379_s_at | -1.7430873 | FGFR3 |
| 238119_at | -1.74402 |  |
| 240307_at | -1.7446042 |  |
| 214703_s_at | -1.7452145 | MAN2B2 |
| 241864_x_at | -1.745297 |  |
| 1568619_s_at | -1.7502829 | ITPRIPL2 |
| 229111_at | -1.7539712 |  |
| 218750_at | -1.75606 | TAF1D |
| 222288_at | -1.7590028 |  |
| 227884_at | -1.7609271 | TAF15 |
| 229206_at | -1.7638075 |  |
| 232257_s_at | -1.7657499 |  |
| 216206_x_at | -1.7663045 | MAP2K7 |
| 204614_at | -1.771139 | SERPINB2 |
| 1559096_x_at | -1.7719553 | FBXO9 |
| 222217_s_at | -1.772377 | SLC27A3 |
| 231597_x_at | -1.773753 |  |
| 236473_at | -1.7738562 | CC2D2A |
| 229422_at | -1.7755513 | NRD1 |
| 214163_at | -1.7769122 | HSPB11 |
| 228238_at | -1.7794112 | GAS5///SNORD44///SNORD47///SNORD76///SNORD77///SNORD79  ///SNORD80///SNORD81 |
| 214918_at | -1.7801136 | HNRNPM |
| 241845_at | -1.784898 |  |
| 228879_at | -1.7859325 | SNORA76///SNORD104 |
| 216887_s_at | -1.787085 | LDB3 |
| 226492_at | -1.7893618 | SEMA6D |
| 244341_at | -1.7925394 |  |
| 238142_at | -1.7937757 | CTD-2574D22.3///OTTHUMG00000177146 |
| 238913_at | -1.7937757 |  |
| 1569142_at | -1.7964313 | TRIM13 |
| 233822_x_at | -1.7985915 |  |
| 230008_at | -1.7994632 | THSD7A |
| 210580_x_at | -1.799628 | LOC101060714///SLX1A-SULT1A3///SLX1B-SULT1A4///SULT1A3  ///SULT1A4 |
| 238070_at | -1.8029065 | CHD1L///LOC101060601 |
| 1552283_s_at | -1.8032284 | LOC100996904///ZDHHC11///ZDHHC11B |
| 205656_at | -1.803729 | PCDH17 |
| 242131_at | -1.8061547 | ATP6 |
| 213459_at | -1.8073443 | RPL37A |
| 240231_at | -1.8075271 |  |
| 212913_at | -1.8083494 | MSH5///MSH5-SAPCD1///SAPCD1 |
| 229569_at | -1.8109224 | OTTHUMG00000180314///RP1-193H18.2 |
| 241716_at | -1.8134636 | HSPD1 |
| 235367_at | -1.8155773 | MYPN |
| 239164_at | -1.8199966 |  |
| 227289_at | -1.8232547 | PCDH17 |
| 1560622_at | -1.830371 |  |
| 1556035_s_at | -1.8306179 | ZNF207 |
| 234432_at | -1.8346417 |  |
| 238620_at | -1.8424143 | OTTHUMG00000176821///RP11-846E15.4 |
| 230087_at | -1.8490907 | PRIMA1 |
| 229498_at | -1.8493958 | MBNL3 |
| 225721_at | -1.8493963 | SYNPO2 |
| 1558783_at | -1.8498145 |  |
| 213703_at | -1.8505379 | LINC00342 |
| 209602_s_at | -1.8543272 | GATA3 |
| 228287_at | -1.854695 | ING5 |
| 1555870_at | -1.8554112 | RNF207 |
| 227719_at | -1.856108 | SMAD9 |
| 221646_s_at | -1.8574703 | LOC100996904///ZDHHC11///ZDHHC11B |
| 227891_s_at | -1.8585886 | TAF15 |
| 239653_at | -1.8594894 |  |
| 224766_at | -1.85949 | LOC100506548///RPL37 |
| 214291_at | -1.8647597 | RPL17///RPL17-C18orf32///SNORD58A///SNORD58B///SNORD58C |
| 231848_x_at | -1.8656257 | ZNF207 |
| 240787_at | -1.8686775 |  |
| 243539_at | -1.8746243 | KIAA1841 |
| 229246_at | -1.8778496 | FLJ44342 |
| 241885_at | -1.8923055 |  |
| 230494_at | -1.8936795 | SLC20A1 |
| 206848_at | -1.8957648 | COX20 |
| 231199_at | -1.896013 | OTTHUMG00000159296///RP11-271C24.3 |
| 204759_at | -1.9003733 | RCBTB2 |
| 220356_at | -1.9005655 | CORIN |
| 1557384_at | -1.9024825 | LOC100506639///ZNF131 |
| 232395_x_at | -1.9065665 | AGBL3 |
| 224372_at | -1.9074653 | C10orf99///CCDC104///ND4 |
| 239493_at | -1.9089488 | RPL7 |
| 206498_at | -1.9125978 | OCA2 |
| 236841_at | -1.9138802 | LOC100134445 |
| 228506_at | -1.9147382 | NSMCE4A |
| 227591_at | -1.916359 | SH3BP5-AS1 |
| 242550_at | -1.9259394 | EIF3B |
| 231292_at | -1.9350587 | EID3 |
| 243367_at | -1.9356279 |  |
| 227517_s_at | -1.9454167 | GAS5///SNORD44///SNORD47///SNORD76///SNORD77///SNORD79  ///SNORD80///SNORD81 |
| 225060_at | -1.9475675 | LRP11 |
| 225768_at | -1.9639565 | NR1D2 |
| 228157_at | -1.9709907 | ZNF207 |
| 226912_at | -1.9715399 | ZDHHC23 |
| 241957_x_at | -1.9781748 | LIN7B |
| 225355_at | -1.9782703 | NEURL1B |
| 1555920_at | -1.9792454 | CBX3 |
| 220770_s_at | -1.9873457 | C5orf54 |
| 228990_at | -1.9877214 | SNHG12///SNORA16A///SNORA44///SNORA61 |
| 241755_at | -1.9969695 | UQCRC2 |
| 221919_at | -1.9992961 | HNRNPA1 |
| 232392_at | -2.0016196 | SRSF3 |
| 218518_at | -2.0154197 | FAM13B |
| 217591_at | -2.0199177 | SKIL |
| 207069_s_at | -2.02153 | SMAD6 |
| 218418_s_at | -2.0217037 | KANK2 |
| 244669_at | -2.0304797 | SNORD50A///SNORD50B |
| 233480_at | -2.0925317 | TMEM43 |
| 1553567_s_at | -2.0941494 | ATP6 |
| 239973_at | -2.0941942 |  |
| 230099_at | -2.0964568 |  |
| 238850_at | -2.1007268 | LINC00461///MIR9-2 |
| 212384_at | -2.1045315 | ATP6V1G2-DDX39B///DDX39B///SNORD84 |
| 212980_at | -2.1147559 | USP34 |
| 235190_at | -2.117191 |  |
| 235693_at | -2.1189482 |  |
| 212907_at | -2.1213012 | SLC30A1 |
| 242837_at | -2.1290684 | SRSF4 |
| 1557810_at | -2.1400392 |  |
| 229130_at | -2.144426 | OTTHUMG00000160053///RP11-774O3.3 |
| 231108_at | -2.1554809 | FUS |
| 230272_at | -2.1659126 | LINC00461///MIR9-2 |
| 205522_at | -2.168778 | HOXD4 |
| 228863_at | -2.1706998 | PCDH17 |
| 229434_at | -2.1857214 |  |
| 234623_x_at | -2.1888082 |  |
| 210358_x_at | -2.190336 | GATA2 |
| 223866_at | -2.1956112 | ARMC2 |
| 203304_at | -2.2062035 | BAMBI |
| 237212_at | -2.2140894 |  |
| 235231_at | -2.2214103 | ZNF789 |
| 213371_at | -2.2446976 | LDB3 |
| 1555653_at | -2.2621107 |  |
| 201565_s_at | -2.2669294 | ID2 |
| 226419_s_at | -2.2687786 | FLJ44342 |
| 224354_at | -2.2979865 |  |
| 230998_at | -2.2990143 | CBX3 |
| 1553575_at | -2.3252683 | ND6 |
| 221768_at | -2.341991 | LOC100996496///SFPQ |
| 222040_at | -2.362259 | HNRNPA1 |
| 242918_at | -2.3824024 | NASP |
| 238199_x_at | -2.3840652 | COX3 |
| 201566_x_at | -2.3896222 | ID2 |
| 204790_at | -2.4003656 | SMAD7 |
| 213894_at | -2.5339077 | THSD7A |
| 232291_at | -2.5390878 | MIR17///MIR17HG///MIR18A///MIR19A///MIR19B1///MIR20A///  MIR92A1 |
| 209710_at | -2.6245477 | GATA2 |
| 217602_at | -2.776002 | LOC101060363///PPIA |
| 211600_at | -2.8282561 | PTPRO |
| 1553538_s_at | -2.9244018 | COX1 |
| 1558048_x_at | -3.0070093 |  |
| 233847_x_at | -3.038725 |  |
| 1553551_s_at | -3.0856209 | ND2 |
| 213931_at | -3.1855135 | ID2///ID2B |
| 229899_s_at | -3.263133 | ZFAS1 |
